# The double homeodomain protein DUX4c is associated with regenerating muscle fibers and RNA-binding proteins

**DOI:** 10.1186/s13395-022-00310-y

**Published:** 2023-03-07

**Authors:** Clothilde Claus, Moriya Slavin, Eugénie Ansseau, Céline Lancelot, Karimatou Bah, Saskia Lassche, Manon Fiévet, Anna Greco, Sara Tomaiuolo, Alexandra Tassin, Virginie Dudome, Benno Kusters, Anne-Emilie Declèves, Dalila Laoudj-Chenivesse, Baziel G. M. van Engelen, Denis Nonclercq, Alexandra Belayew, Nir Kalisman, Frédérique Coppée

**Affiliations:** 1 Laboratory of Metabolic and Molecular Biochemistry, Research Institute for Health Sciences and Technology, University of Mons, 6, Avenue du Champs de Mars, B-7000 Mons, Belgium; 2 Department of Biological Chemistry, the Alexander Silberman Institute of Life Sciences, Hebrew University of Jerusalem, Jerusalem, Israel; 3 Department of Neurology, Donders Institute for Brain, Cognition and Behaviour, Radboud University Medical Center, 6525 GA Nijmegen, The Netherlands; 4 Department of Neurology, Zuyderland Medical Center, Heerlen, the Netherlands; 5 Laboratory of Respiratory Physiology and Rehabilitation, Research Institute for Health Sciences and Technology, University of Mons, 6, Avenue du Champs de Mars, B-7000 Mons, Belgium; 6 Department of Pathology, Donders Institute for Brain, Cognition and Behaviour, Radboud University Medical Center, 6525 GA Nijmegen, The Netherlands; 7 PhyMedExp, University of Montpellier, INSERM, CNRS, Montpellier, France; 8 Laboratory of Histology, Research Institute for Health Sciences and Technology, University of Mons, 6, Avenue du Champs de Mars, B-7000 Mons, Belgium

**Keywords:** *DUX* genes, RNA-binding interactors, HA-binding protein C1qBP, FUS, SFPQ, Differentiation, Fibrosis, M1/M2 macrophages, Muscle regeneration, Testis

## Abstract

**Background:**

We have previously demonstrated that double homeobox 4 centromeric (*DUX4C*) encoded for a functional DUX4c protein upregulated in dystrophic skeletal muscles. Based on gain- and loss-of-function studies we have proposed DUX4c involvement in muscle regeneration. Here, we provide further evidence for such a role in skeletal muscles from patients affected with facioscapulohumeral muscular dystrophy (FSHD).

**Methods:**

DUX4c was studied at RNA and protein levels in FSHD muscle cell cultures and biopsies. Its protein partners were co-purified and identified by mass spectrometry. Endogenous DUX4c was detected in FSHD muscle sections with either its partners or regeneration markers using co-immunofluorescence or in situ proximity ligation assay.

**Results:**

We identified new alternatively spliced *DUX4C* transcripts and confirmed DUX4c immunodetection in rare FSHD muscle cells in primary culture. DUX4c was detected in nuclei, cytoplasm or at cell–cell contacts between myocytes and interacted sporadically with specific RNA-binding proteins involved, a.o., in muscle differentiation, repair, and mass maintenance.

In FSHD muscle sections, DUX4c was found in fibers with unusual shape or central/delocalized nuclei (a regeneration feature) staining for developmental myosin heavy chain, MYOD or presenting intense desmin labeling. Some couples of myocytes/fibers locally exhibited peripheral DUX4c-positive areas that were very close to each other, but in distinct cells. MYOD or intense desmin staining at these locations suggested an imminent muscle cell fusion.

We further demonstrated DUX4c interaction with its major protein partner, C1qBP, inside myocytes/myofibers that presented features of regeneration. On adjacent muscle sections, we could unexpectedly detect DUX4 (the FSHD causal protein) and its interaction with C1qBP in fusing myocytes/fibers.

**Conclusions:**

DUX4c upregulation in FSHD muscles suggests it contributes not only to the pathology but also, based on its protein partners and specific markers, to attempts at muscle regeneration. The presence of both DUX4 and DUX4c in regenerating FSHD muscle cells suggests DUX4 could compete with normal DUX4c functions, thus explaining why skeletal muscle is particularly sensitive to DUX4 toxicity. Caution should be exerted with therapeutic agents aiming for DUX4 suppression because they might also repress the highly similar DUX4c and interfere with its physiological role.

**Graphical Abstract:**

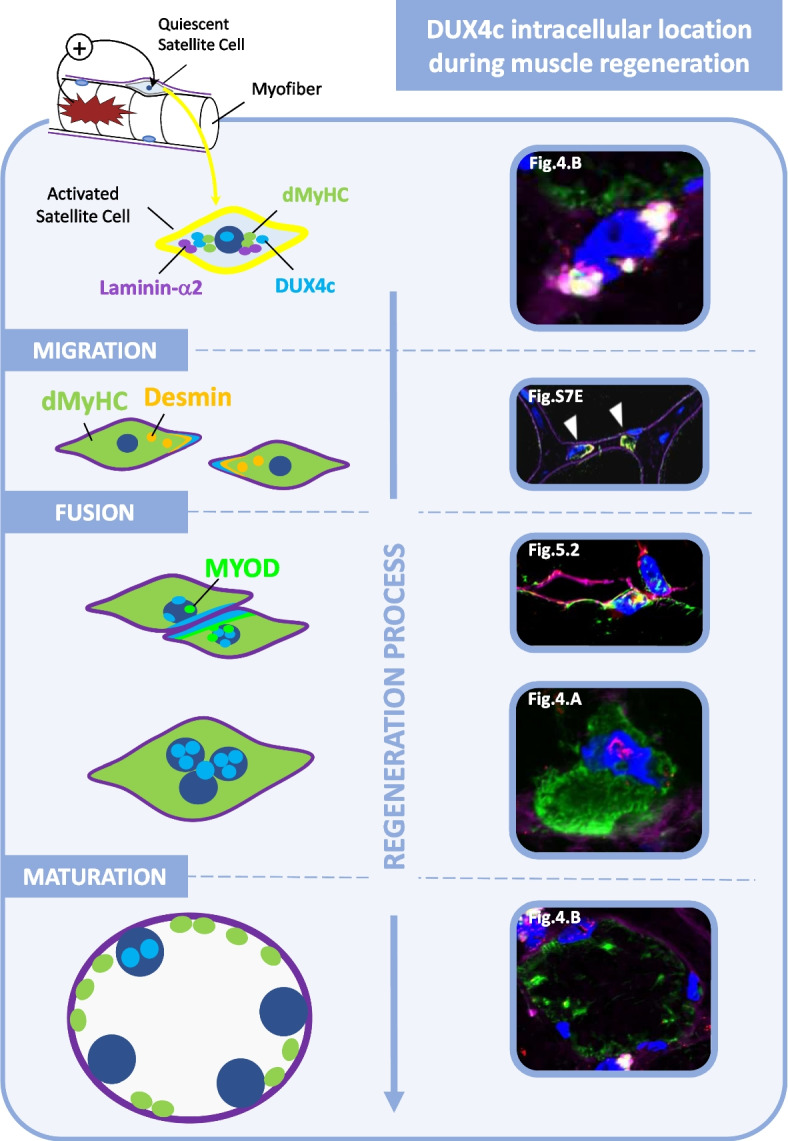

**Supplementary Information:**

The online version contains supplementary material available at 10.1186/s13395-022-00310-y.

## Introduction

**Fig. 1:**
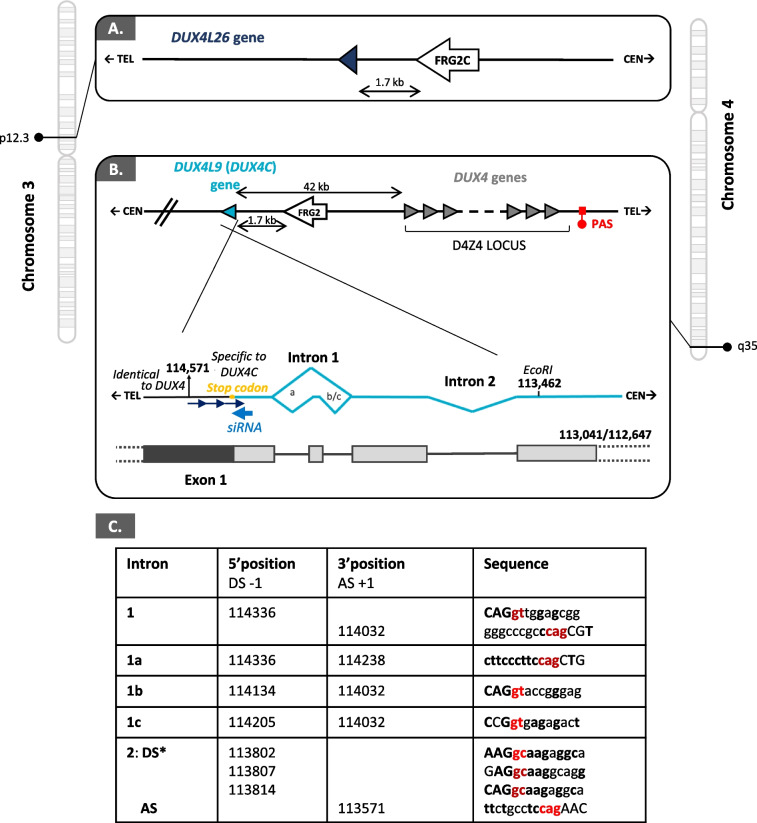
*DUX4C* and *DUX4L26* gene maps and *DUX4C* alternative intronic transcripts. Comparison of the gene locations on chromosome 3p12.3 and 4q35 regions presenting either *DUXL26* next to *FRG2C* (**A**) or *DUX4C* next to *FRG2* (**B**). (**B**, bottom) Schematic representation of the endogenous *DUX4C* 3’ UTR sequences found using RT-PCR in primary and immortalized human myoblasts (some amplified fragments are shown in Fig. S1). The intron 2 was previously identified in C2C12 cells transfected with a *DUX4C* genomic construct [3]. **C** The table summarizes the donor (DS) and acceptor (AS) splice sites sequences and their coordinates (respectively, last 3′ and first 5′ nucleotide position in exon) on Genbank #AF146191. All correspond to canonical (or reported atypical*) splice sites. The letters in bold correspond to the highest nucleotide frequency in the corresponding consensus position. In red, the 5′ and 3′ intron sequences used in splice site classifications [11]. The complete *DUX4C* sequences obtained from several cell cultures after its cDNA cloning are available in Table S2. PAS: polyadenylation signal

Double homeobox 4 centromeric (*DUX4C*), also named *DUX4L9* (DUX4-like 9), is located on chromosome 4q35 and is referenced as a pseudogene in the ENSEMBL genome database (GRCh38.p13, July 2021). The pseudogene-annotated regions are generally excluded from functional analyses and high throughput experiments may restrict the quantification of lowly expressed pseudogenes (reviewed in [1]). These authors [1] also propose that the pseudogene term should only be used in the context where such a genomic region demonstrably lacks biological activity. We have previously shown that *DUX4C* transcripts were expressed in primary human muscle cells leading to the synthesis of a 47-kDa DUX4c protein [2, 3]. DUX4c is highly similar to DUX4 whose misexpression in skeletal muscle causes facioscapulohumeral muscular dystrophy (FSHD) [4–9]. The DUX4/DUX4c sequence identity extends over the first 342 residues encompassing both homeodomains, while the remaining 22 carboxyl-terminal residues are 40% identical [2]. Nevertheless, the gene showing the highest identity with *DUX4C* is *DUXO* (also named *DUX4L26*) on chromosome 3p12.3. *DUX4C* and *DUX4L26* similarity extends to neighboring genomic sequences since both genes map at the same distance from an *FRG2* related gene (Fig. 1A, B) [10].

DUX4c loss- or gain-of-function studies in human muscle cells showed that excess DUX4c affected proliferation of human TE671 rhabdomyosarcoma or primary muscle cells and inhibited their differentiation [2, 3, 12]. DUX4c excess also interfered with mouse myoblast fusion in cell cultures [13, 14]. We further showed that DUX4c excess at a later stage of primary myoblast differentiation altered the organization of myofibrils and led to the formation of large clusters of nuclei [3]. Such myofibril and nuclear disorganizations are characteristic of primary FSHD disorganized myotubes [3, 15]. In addition, high endogenous DUX4c levels were detected in such FSHD myotubes and myofibers as well as in total protein extracts of FSHD muscle biopsies [2, 3, 12]. We also demonstrated that only an siRNA targeting DUX4c, not DUX4, could reverse the disorganized FSHD myotube phenotype [16]. In a transcriptomic study on primary mouse myoblasts transduced with retroviruses expressing DUX4 or DUX4c, Knopp et al. [14] showed that in contrast to DUX4 targets, genes deregulated by DUX4c were associated with muscle development. Previous studies had already suggested that DUX4c might be involved in muscle regeneration [2, 3, 12, 13]. In addition, myogenic miRNAs were induced by DUX4c overexpression in primary myoblasts [17]. Furthermore, during normal myogenic differentiation, the induction of the KLF15 transcription factor contributed to DUX4c but not DUX4 overexpression [18]. In agreement with these observations, the DUX4c protein was detected in primary healthy human myoblast extracts and induced upon differentiation [2, 3, 12]. In a previous study, our group has identified and validated several RNA-binding proteins (RBPs) as DUX4c partners in cells overexpressing DUX4c and suggested they could be part of mRNP (messenger ribonucleoprotein) granule complexes containing IGF2 mRNA-binding protein 1 (IGF2BP1/IMP1) and involved in mRNA fate [12].

In the present study, we want to confirm that *DUX4C* is not a pseudogene and to bring new evidence that it encodes a protein associated with muscle regeneration. We first characterize new *DUX4C* RNA splicing isoforms that differ from one muscle cell culture to another and even within a given culture, but present 2 main mRNA 3′ ends in primary muscle cells. We also analyze DUX4c protein expression in more FSHD muscle (several types including *tibialis anterior*) sections than previously published [3, 12], and in testis. By co-immunofluorescence, we confirm that endogenous DUX4c protein interacts with several RBPs both in human muscle cells and testis. Furthermore, by co-immunoprecipitation coupled to mass spectrometry analyses, we find the RBP Complement component 1 Q subcomponent-binding protein (C1qBP), previously validated as a DUX4 interactor [12, 19], is the major DUX4c protein partner. Finally, we specifically immunodetect DUX4c and DUX4 in FSHD muscle sections, in myofibers that express regeneration markers and where both proteins interact with C1qBP. These data further indicate a role for DUX4c in muscle regeneration and suggest DUX4 could compete with this function in FSHD.

## Methods

### Ethics statement

Primary human myoblasts were derived from muscle biopsies performed according to the ethical and legislative rules of France and approved by the ethical committee of CHU de Villeneuve (Montpellier, France) [15]. Immortalized cells were obtained from the Institute of Myology (Paris) and the Wellstone Center for FSHD (University of Massachusetts Medical School, Worcester) as published in [20, 21]. DMD biopsy sections were the ones described in [12], kindly provided by Dr. François Rivier (CHU de Villeneuve, Montpellier, France). For biopsy muscle sections, patients were recruited at the Radboud University Medical Center. The Medical Ethics Review Committee region Arnhem-Nijmegen approved associated studies (n° 2011/181 [22] and 2018/4246). Additional muscle and testis sections were provided by the Biobank of the Institute of Pathology and Genetics (Gosselies, Belgium). Informed consent was obtained from all subjects. The use of this material was approved by the ethics committee of the University of Mons (ref # A901) and the ethics committee of ULB-Erasme (Brussels ref #B2011/003 and #P2015/516).

### Cell cultures

Total muscle explant-derived cells were purified by Magnetic-activated cell sorting (Milteny Biotech) using anti-CD56 antibody (Table S1). Myoblast identity was determined by desmin immunostaining (> 98%). The primary and immortalized myoblasts were grown, respectively, in DMEM with high glucose and l-glutamine (Lonza), 10% fetal bovine serum (FBS) (Invitrogen), 1% Ultroser G (Pall BioSepra, Cergy-St-Christophe, France), and gentamicin (50 μg/ml, Sigma-Aldrich) or DMEM high glucose supplemented with 16.5% medium 199 (Lonza), 15% FBS, Ultroser G, HEPES 1 M (Sigma-Aldrich), zinc sulfate (Sigma ®-Aldrich, vitamin B12 (Sigma-Aldrich), and penicillin/streptomycin (pen/strep) at 37 °C under atmosphere with 5% CO_2_. For myogenic differentiation, cells were cultured on Matrigel-coated culture dishes and a differentiation medium was added after cells reached 100% confluence. This medium was composed of DMEM/gentamicin (50 μg/ml) with 2% FBS for primary cells and DMEM high glucose, medium 199 supplemented with 0.5% insulin, 1% apo-transferrin (Sigma-Aldrich), 2% HEPES 1 M and pen/strep for immortalized cells. HEK293 were grown in DMEM high glucose-10% FBS and pen/strep. Transfection of primary cells was previously reported [3]. The KLF15 expression vector was a generous gift of Prof. Yegor Vassetzky [18].

### 3′RACE

Total RNA was extracted, retro-transcribed with a procedure for high secondary structure [5] and 3′RACE experiments were performed as previously described [2] except that 500 ng DNase-treated RNA and 4 μl of SuperScript III were used for RT. For the *DUX4C* 3′RACE, 2.5 μl cDNA were used for PCR with primer 5′-*AGATGCCAGCCATCCAGGCG*-3′ and the 3′ outer RLM-RACE primer (Ambion) and the conditions were 3 min at 98 °C, followed by 10 s at 98 °C, 10 s at 60 °C, and 5 s at 72 °C for 25 cycles, followed by 5 min at 72 °C. For the inner PCR with primer 5′-*ACAGTCACCTCCAGCCTGTTAT*-3′ and the 3′ inner RLM-RACE primer (Ambion), 1.5 μl of outer PCR product were used and the conditions were 3 min at 98 °C, 20 cycles of 10 s at 98 °C, 10 s at 62 °C, 7 s at 72 °C followed by 5 min at 72 °C. For the second inner PCR with primer 5′-*GAGCTCCTGTAGACACCAGAG*-3′ and the 3′ inner RLM-RACE primer, 1 μl of the first inner PCR product was used and the conditions were 3 min at 98 °C and 20 cycles of 10 s at 98 °C, 5 s at 62 °C, 10 s at 72 °C followed by 5 min at 72 °C. Only one couple of outer and inner primers was used for primary cells. The PCR products were cloned in a *pJET1.2* plasmid and sequenced. Positive controls correspond to myoblasts transfected with *p7.5-kb-DUX4c* [2] or *pHalo-DUX4c* [12], using Lipofectamine 2000 (Invitrogen) according to the manufacturer’s instructions.

### Co-purification of protein partners and mass spectrometry

HEK293 cells were transfected with the *pHaloTag-DUX4c* or *-GFP* expression vector using Lipofectamine 2000 (Invitrogen). Twenty-four hours later, cells were lysed and the protein extract was used directly for purification on Halo-link resin. Covalent capture purifications were performed as described in [12] with an incubation time of 3 days at 4 °C. Proteins were eluted with TEV protease treatment and were prepared for mass spectrometry using the Filter-Aided Sample Preparation (FASP) [23]. We followed the established procedure with protein alkylation by iodoacetamide and digestion by trypsin. The digest was acidified by adding TFA to 0.5% and then desalted by filtration on C18 stage-tips. The digest was eluted, dried in a Speed-Vac, dissolved in reconstitution solution (97% water, 3% acetonitrile, 0.1% formic acid), and immediately analyzed in a Q-Exactive Plus mass-spectrometer with the following settings: Buffer A–water with 0.1% formic acid, Buffer B–acetonitrile with 0.1% formic acid. Gradient was rising linearly from 0 to 45% buffer B over 90 min, then rising to 80% buffer B over 5 min. Overall, we analyzed two biological replicates, each with two technical replicates, for each condition (EGFP or DUX4c).

The RAW files were analyzed in a single computational run using MaxQuant software version 1.5 [24]. Default MaxQuant settings were used, and the sequence database comprised all human proteins (downloaded from UniProt) augmented with the sequences of DUX4c, GFP, HALO tag, and the TEV protease. Next, the ‘proteinGroups.txt’ output file was loaded to Perseus [25]. We filtered out the reverse proteins and contaminations, transformed the data to logarithmic scale, and grouped the samples according to replicates. For LFQ intensities that were missing, we imputed values from a normal distribution. We used a two-sample test, with a permutation-based FDR of 1% and ‘s0’ (minimal fold change) value of 2. We generated a volcano plot presenting the proteins in the “*t*-test Difference” vs. “-Log *t*-test *p*-value” coordinate system. Any point that is over the significance curves is likely a significant hit.

### Rat antisera against DUX4c

Two antigenic DUX4c-specific peptides were designed, synthesized, and co-injected to rats allowing to produce specific antisera (Eurogentec, Seraing, Belgium) (Fig. S2A, B). The immunogenicity of the rat antisera that gave the best signal/noise ratio (on fixed muscle cells transfected with the *pHaloTag-DUX4c* expression vector, data not shown) was confirmed by ELISA against each DUX4c peptide (Eurogentec). The antiserum was purified by affinity chromatography against the 860 antigenic peptide.

### Immunohistochemistry, immunofluorescence, and proximity ligation assay

Muscle sections or cells were fixed in 4% paraformaldehyde or for 10 min at 4 °C in acetone and treated as described in [26] or [12], except for the use of Tyramide Signal Amplification (TSA) technology (Perkin Elmer) to detect low abundance protein in muscle sections. Briefly, for immunohistochemistry, the sections were pretreated for antigen unmasking via heating and sequential incubations with H_2_O_2_, avidin and biotin. Then, the sections were rinsed and blocked in 0.05% casein. The slides were subsequently incubated for 1 h at RT or at 4 °C O/N with rabbit anti-DUX4c purified serum (1/20 or 1/50), followed by a 30-min incubation with a secondary antibody coupled to biotin. The TSA technology was used as described by the manufacturer, followed by incubation with 0.02% 3, 3'-diaminobenzidine-0.01% H_2_O_2_ in PBS. Counterstaining was performed with either hematoxilin alone or combined with luxol fast blue-periodic-acid Schiff.

For immunofluorescence, cells or sections were permeabilized with 0.5% Triton X-100 in PBS and blocked with 20% FBS in PBS. Appropriate primary antibodies were diluted in PBS containing 0.5% BSA (Table S1) and incubated O/N at 4 °C. After washing, the appropriate secondary antibodies coupled to Alexa Fluor (Invitrogen) diluted in PBS containing 0.5% BSA were incubated for 1 h at RT. In situ proximity ligation assay (PLA; Duolink, Sigma-Aldrich) was performed following the manufacturer’s instructions as previously described in [12]. Slides were finally mounted with or without the 4,6-diamidino-2-phenylindole (DAPI) (either from the duolink kit or in SlowFade Gold antifade reagent).

On the testis sections, a co-immunostaining method using two antisera raised in the same species was applied (as detailed in [27]). Briefly, after antigen unmasking and blocking (0.05% casein) treatment, sections were incubated O/N with the first primary antibody, followed by the corresponding biotinylated secondary antibody (1/50) and Texas Red-conjugated streptavidin (1/50). Next, the sections were rinsed and exposed to microwave irradiation to denature proteins, then rinsed again and incubated overnight at 4 °C with the second primary antibody, followed by the fluorescein isothiocyanate (FITC)-conjugated secondary antibody. Slides were finally mounted with DAPI Slowfade reagent.

For the negative controls, the primary antiserum was replaced by either the pre-immune serum, or a non-immune serum. In addition, a competition with the DUX4c- or DUX4-immunogenic peptide/domain was performed for the antiserum/body (overnight incubation at 4 °C with the immunogenic peptide/domain in a fivefold molar excess [2] and Additional information in [28]).

### Image acquisition

Images were acquired with either a Leitz Orthoplan microcope and a Leica DC 300F camera (immunohistochemistry), a Nikon Eclipse 80i (equipped with filters allowing the detection of weak fluorescence and with a DS-U3 DS Camera control Unit at room temperature) or Confocal Ti2 (equipped with A1 FLOV Camera control Unit) microscope allowing Z-stacking captures. Plan Fluor 20 X, Plan Fluor 409, and 609 Apo-VC high-resolution oil immersion or Plan Apo Lambda S 40XC Sil objectives were used, with 350, 480, and 540 nm excitation for DAPI, FITC, and tetramethyl rhodamine isothiocyanate (TRITC) channels, respectively. The acquisition software was NIS element-BR analysis software including 3D reconstruction. ImageJ was used for image merging and analyses. Fields were not randomly chosen but selected on the basis of a clear DUX4c, DUX4, or PLA signal detection, apart for the one involving dMyHC immunodetection where all the sections were analyzed.

### Western blot

Twenty micrograms of total protein extracts were separated by electrophoresis (4–12% PAGE-SDS) in MOPS buffer at 100 V for 3h30 and transferred to a nitrocellulose membrane at 260 mA for 1h45 in a blotting buffer (PBS, 25 mM Tris, 192 mM Glycine, 20% methanol). Protein transfer was confirmed by Ponceau red staining of the membrane. After rinsing in PBS and blocking with PBS-milk 5% for 1 h at RT, the membrane was incubated overnight with either primary antibodies: MAb 9A12 mouse anti-DUX4 (1/1000), rat anti-DUX4c 860 serum (1/1000), or rabbit anti-DUX4c serum (1/1000) diluted in PBS-2% BSA followed by rinsing in PBS and incubation 1 h at RT with secondary antibodies coupled to HRP at 1/5000 dilution. Revelation was performed with either the Super Signal West Femto Maximum Sensitivity Substrate (Thermo Scientific) for endogenous protein detection or Lumi-Light Western Blotting Substrate (Roche) for overexpressed protein detection on Hyperfilm ECL (Amersham).

### Statistical analyses

Results are presented as mean values ± SD. The level for statistical significance was defined as *p* < 0.05. Analyses were carried out using Sigma Plot 11.0. Differences between data groups were evaluated for significance using unpaired *t* test.

## Results

### *DUX4C* transcripts in primary muscle cells

Because databases still classified *DUX4C* among pseudogenes, we first wanted to further confirm it is a functional gene. In our initial *DUX4C* characterization [2], we had identified a functional promoter leading to the transcription of an mRNA encompassing the full ORF (contained in a single exon) followed by a 3′UTR, both in muscle cells transfected with a genomic *DUX4C* fragment and in human primary myoblasts/myotubes. A single spliced-out intron (in 3′UTR) was identified in transfected cells but not in a few primary muscle cells analyzed in parallel [3].

Using 3′RACE, we have now detected several *DUX4C* spliced transcripts in additional human primary or immortalized muscle cell ‘line’ cultures (Fig. 1B, C, Fig. S1). We confirmed the alternative spliced forms by sequencing of individually cloned RT-PCR products derived from either proliferating or differentiating muscle cells. However, some sequence variability occurred among primary or immortalized cell ‘lines’ or independent cultures of the same cell ‘line’. Indeed, several bands ranging from about 0.7 to 2.0 kb were detected by electrophoresis on 1% agarose gel among the RT-PCR products of the 9 primary muscle cultures or the 8 immortalized cell lines analyzed (some examples of the amplicon diversity are given in Fig. S1A, B**)**. Altogether, DNA was sequenced from three bands at ~ 1.0, 1.2 (arrows in Fig. S1), and 1.4 kb sporadically found in both immortalized and primary cell cultures and corresponded to distinct splicing forms (illustrated in Fig. 1B, C and available in Table S2). In addition, a preliminary experiment in which KLF15 was overexpressed in an FSHD primary cell culture showed a change in the *DUX4C* RT-PCR products (Fig. S1C).

The UCSC Genome Assembly (December 2013; GRCh38/hg38) reported several DUX4-like genes on several chromosomes most of which were expressed at low levels in brain and testis (Table S3). The sequences determined above were 900- to 1450-bp long with 100% identities to coordinates 190,021,552 to 190,020,100 on chromosome 4, corresponding to the *DUX4C* gene. Moreover, the ENCODE Registry of candidate *cis*-Regulatory Elements (cCREs) in the human genome (representative DNase hypersensitive sites across ENCODE and Roadmap Epigenomics samples supported by either H3K4me3 or H3K27ac histone marks of open chromatin) has identified three proximal enhancer-like signatures (pELS) within 2 kb of the *DUX4C* transcription start site (TSS) (ENCODE Accession #: EH38E2351642; EH38E2351641; EH38E2351640). One of these (chr4:190,022,729–190,023,078) maps in the *DUX4C* 5′ region and corresponds to the functional promoter we have experimentally determined [2]. In ENSEMBL, the larger overlapping chr4:190,019,400–190,023,600 region is also classified as a promoter with several transcription factor binding sites including a.o. PITX1, that is specifically increased in FSHD muscles [5] (regulatory feature: ENSR00000746270). Furthermore, we had previously demonstrated that a specific siRNA targeting the *DUX4C 3′UTR*, i.e., an mRNA transcribed from this genomic region (Fig. 1B) abolished synthesis of a DUX4c protein [2, 3]. All together, these new and earlier data support the concept that *DUX4C* is functional and actively transcribed in healthy and FSHD muscle cells.

### DUX4c protein detection in primary FSHD muscle cells

After characterization of new *DUX4C* transcripts, we wanted to immunodetect the encoded protein. Because of DUX4c low abundance, we first selected immortalized FSHD cell lines derived from biceps or deltoid in which we had observed a clear *DUX4C* RT-PCR product upon differentiation (Fig. S1A). We performed DUX4c immunodetection by western blot (WB) in these cell lines with the rabbit antiserum we had previously described [2, 3, 12] and that had already been validated by (i) peptide competition in primary cells (Figure 3B in [2]) and (ii) in muscle cells treated with a specific siRNA (Figure S6 in [3]). However, of the 8 cell lines tested (4 healthy and 4 FSHD), we could only detect DUX4c in one FSHD cell line, in which we observed a weak band at the expected 47-kDa size in the total and nuclear extracts, but not in the cytoplasmic extract. In previous studies of such cells and in FSHD primary myotubes, we could only stain DUX4c by immunofluorescence in the nuclei and in the cytoplasm of scarce myotubes [3, 12]. This low abundance could explain the difficulty we found here to immunodetect DUX4c by WB.

In order to define when DUX4c was mostly expressed, we performed a differentiation time-course of primary cultures derived from two distinct muscles of two patients (Table S4) and detected DUX4c by immunofluorescence. We used two specific antisera raised and purified against different peptides found in DUX4c but not in DUX4 (Fig. S2A). The first antiserum was raised in rabbit and used in the WB above and in our previous studies [2, 3, 12]. The second one was a rat serum we have developed in the present study to confirm DUX4c detection and to allow triple co-immunodetection (see below). Both purified antisera were validated on extracts of cells transfected with either a DUX4c- or DUX4-expression vector or an empty vector, and WB demonstrated their specificity (Fig. S2B).

**Fig. 2:**
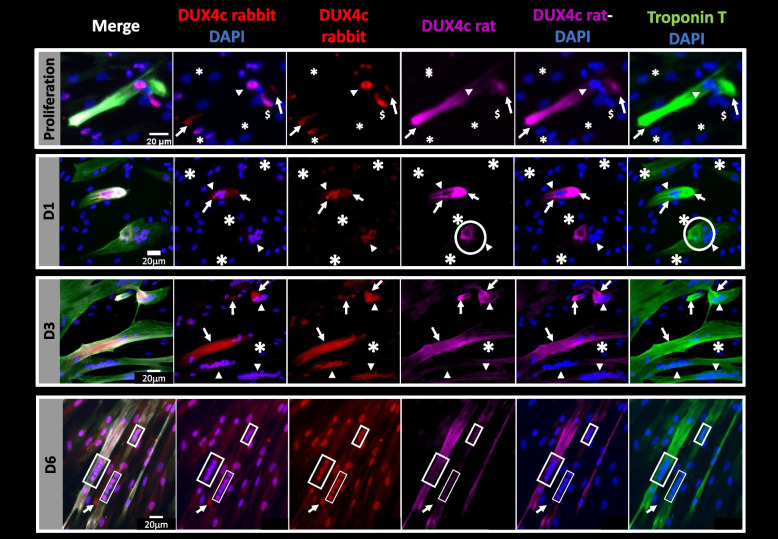
(Upper panels) Time-course of DUX4c expression in primary FSHD muscle cells. FSHD primary muscle cells were grown and fixed with PAF either in proliferation (P) or after incubation in a differentiation medium at days 1 (D1), 3 (D3), or 6 (D6). DUX4c and Troponin T (TnT) were co-immunodetected using both rabbit and rat anti-DUX4c sera and the mouse anti-TnT antibody, followed by the appropriate secondary antibodies coupled to either Alexa Fluor 555 (red, rabbit anti-DUX4c), 647 (shown in purple, rat anti-DUX4c), or 488 (green, TnT). Arrows point to DUX4c cytoplasmic labeling and asterisks to nuclei which do not present DUX4c labeling. The strongest DUX4c nuclear staining is detected either in TnT-expressing cells (arrowheads) or in the nuclei of cells close to the ones expressing TnT ($). In proliferating cells (P), nuclei inside clusters are rounder and smaller (< 10 µm) compared to single cell nuclei of the same culture (also see Fig. S3) Circles highlight cytoplasmic accumulation of TnT that co-localizes with DUX4c either using the rat (D1) or the rabbit (D3 in Fig. S3) antiserum. Rectangles in D6 images indicate DUX4c detection using both DUX4c antisera in aligned nuclei of myotubes. These very close nuclei suggest that fusion has occurred recently [30]. The selected images correspond to magnification of rare regions boxed in Fig. S3A. Three to ten fields were analyzed per time in two cultures derived from two different muscles (Table S4)

In the FSHD primary myoblast cultures, we observed about 1% of cells (22 in a total of 1700 analyzed cells from both cultures) that already expressed the myotube marker Troponin T (TnT), despite a cell confluency of only ~ 80%. These TnT-positive cells contained one to several (up to 23) nuclei and a few of these cells presented a cluster with more than 9 nuclei (Fig. 2, Fig. S3), suggesting either TnT misexpression in unfused myocytes or the result of an abnormal early fusion leading to TnT expression. Using the rabbit antiserum, we noticed that DUX4c staining intensity was highly variable from one cell to another, with the strongest signal detected either in TnT-expressing cells (arrowheads) or in the nuclei of cells close to the ones expressing TnT (Fig. 2). Some nuclei were unlabeled (asterisks). As already reported in differentiating immortalized cells [12], a cytoplasmic DUX4c staining could also be observed, more specifically in these ‘early differentiating’ primary cells (Fig. 2, Fig. S3). The rat antiserum only detected the cytoplasmic DUX4c fraction and showed a partial overlap with the rabbit antiserum staining (arrows). Altogether, cytoplasmic DUX4c detection with two antisera targeting distinct epitopes strongly supported their specificity. The strongest cytoplasmic signals observed with the rat antiserum suggested either it was more sensitive and could detect lower amounts of DUX4c or the epitopes recognized by the rat or rabbit antiserum had different accessibility according to specific post-translational modifications. Such modifications were indeed reported for the homologous and highly similar DUX4 protein (Fig. S2A) [29].

During the differentiation time-course (Fig. 2, Fig. S3), we observed nuclear (rabbit antiserum) or cytoplasmic (both antisera) DUX4c staining (arrows) similar to the ones described above in proliferation. In one muscle cell culture (derived from the *Serratus posterior inferior* muscle, with a myotube fusion index (MFI) of 70%, Table S4), we observed a sharp intensity drop of DUX4c nuclear staining at day 6 compared to days 1 and 3. For a second culture (derived from another muscle type: *Serratus posterior superior*), the intensity drop was already observed at day 3 but nuclear staining was again observed at day 6 and could be associated with a very low MFI (14.2%) (Fig. S4). The variation in DUX4c staining intensity (nuclear and cytoplasmic) during the differentiation time-course (as previously shown for immortalized cells in [12]) and in each culture at specific times, as well as the fact that most of the strongest cytoplasmic signals observed with the rat anti-DUX4c serum overlapped with the rabbit antiserum staining further confirmed the antibody specificities (number of analyzed nuclei at D1: 4269, at D3: 10,635 and at D6: 3689 with less than 1% presenting a high DUX4c staining). DUX4c immunostaining mainly co-localized with intense TnT staining in rare cells or specific areas (Fig. 2 and Fig. S3), in accordance with similar observations in healthy primary myotubes following exogenous DUX4c expression (Figure 2 in [3]). During the whole differentiation process we found cytoplasmic and nuclear DUX4c staining in small TnT-expressing cells containing one to four nuclei. Some of these cells presented a strong DUX4c nuclear staining (arrowheads in Fig. 2 and Fig. S3B, # in Fig. S3A) and appeared like comets with a DUX4c cytoplasmic staining at one side (as reported in immortalized cells, [12]). We also observed DUX4c in larger myotubes, specifically in TnT intense areas found either next to clusters of nuclei (circle), at a tip of the cell, next to the membrane, at cell–cell contacts or next to a very thin extension (characteristic of DUX4-expressing muscle cells, [14]). At day 6, we unexpectedly observed in only two myotubes close to each other (of all the analyzed fields) a DUX4c nuclear labeling with the rat antiserum (Fig. 2 and box 1 in Fig. S3A). These two myotubes presented clusters of aligned and very close nuclei (2 groups of 5 and one of 2). Between these two myotubes, a cell with a single large nucleus and no TnT detection presented an intense cytoplasmic DUX4c staining, with the rabbit antiserum only (arrows). This observation supported the idea that DUX4c moved among different intracellular locations for specific limited times and might adopt several conformations following post-translational modification (as stated above).

All these intracellular localizations for most of which DUX4c was detected with both antisera or co-detected in the same rare cells (or close cells) suggest a subtle and temporal DUX4c regulation (see discussion).

### DUX4c detection in healthy and FSHD skeletal muscles

After studying DUX4c expression in cell cultures, we wanted to detect the protein in muscle biopsies. We first performed immunohistochemistry on muscle sections with the rabbit anti-DUX4c serum. Because of its very low expression in healthy skeletal muscle, a highly sensitive amplification technique was required to detect DUX4c in peripheral nuclei of muscle fibers (Fig. S5A). In contrast, in some FSHD muscle sections, standard immunostaining procedures allowed DUX4c detection as expected from its reported upregulation in FSHD [3, 12]. In FSHD muscles, DUX4c staining was detected in peripheral nuclei and also in central/delocalized ones (Fig. S5B). Out of the four muscles (from omopexia surgery, Table S4), we analyzed by immunohistochemistry, the strongest DUX4c staining was detected in a group of about five fibers showing an angular morphology (Fig. S5B). DUX4c was apparently in either peripheral (arrows) or delocalized nuclei (DN) with a surrounding sarcoplasm staining (star) (as previously observed by immunofluorescence in two adjacent fibers, Figure S9 in [12]). In addition, DUX4c staining extended to just under the basement or sarcoplasmic membrane (arrowheads) at the fiber periphery. On multiple biopsies obtained from individual patients, either 2/2 (same muscle, F9, see below) or 3/4 (distinct muscles, F-P1), respectively, presented a DUX4c staining. For the latter, heterogeneous staining intensity levels occurred among different muscles (treated in parallel) (Fig. S5C).

**Fig. 3:**
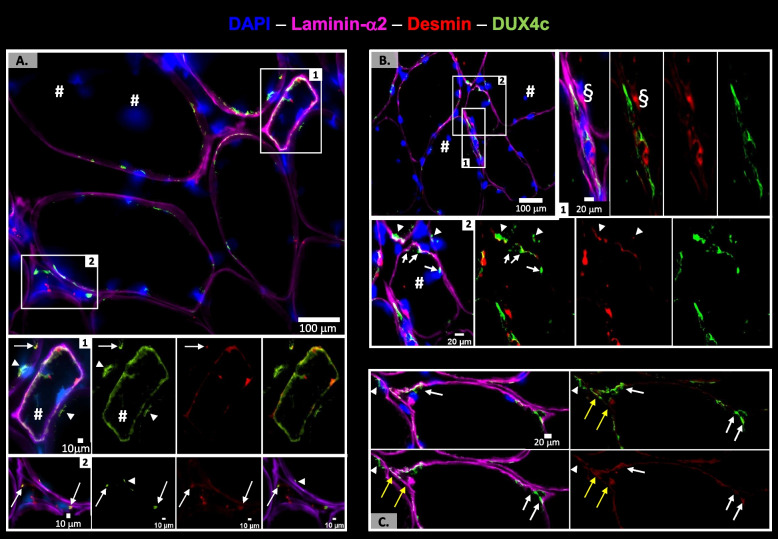
DUX4c is immunodetected in myofibers either hypotrophic or with an unusual shape. DUX4c, desmin, and laminin-α2 were detected using the rabbit anti-DUX4c serum, mouse anti-desmin and rat anti-laminin-α2 sera followed by appropriate secondary antibodies coupled to AlexaFluor 488 (green), 555 (red), or 647 (purple), respectively. Nuclei were stained with DAPI (blue). Staining was observed by epifluorescence microscopy. **A** DUX4c immunostaining in hypotrophic fibers with either a rectangular (box 1) or a flat (box 2) morphology next to other myofibers with either peripheral or central (#) nuclei. DUX4c was also observed as short lines at the periphery of these adjacent fibers. Magnified box 1 shows DUX4c detection around the whole myofiber periphery, around one peripheral nucleus and next to a central nucleus (#). We also observed next to the peripheral nuclei, a DUX4c staining in the adjacent fibers (arrowhead) and a partial co-detection with desmin (arrow). Magnified box 2 shows DUX4c staining in dots, one of which is inside the myofiber and co-localizes with desmin at its two tips (arrows), and DUX4c again appears as a line in an adjacent fiber (arrowhead). As previously published [12], desmin is also detected without DUX4c staining. **B** DUX4c detection as in (**A**, box 2) (box 1) and in a normal-size fiber presenting an unusual shape (box 2) next to adjacent fibers with central nuclei (#). Magnified box 1 shows a very flat myofiber. DUX4c is detected around the two close nuclei and at the fiber tips although intense desmin is present. Next to this fiber, another nucleus (§) presents an intense desmin staining, mainly on one side, co-detected with an intense laminin-α2 signal. Magnified box 2 shows the myofiber with an abnormal shape at one round tip presenting cytoplasmic DUX4c (arrows) either at this tip or next to a large peripheral nucleus. The close adjacent fibers also present cytoplasmic DUX4c either at one tip or next to a large peripheral nucleus (arrowheads). **C** Another myofiber showing unusual shape with two triangular tips containing DUX4c labeling (white arrows). One tip also presents an intense internal desmin staining that is co-detected with intense laminin-α2 signal (yellow arrows)

**Fig. 4:**
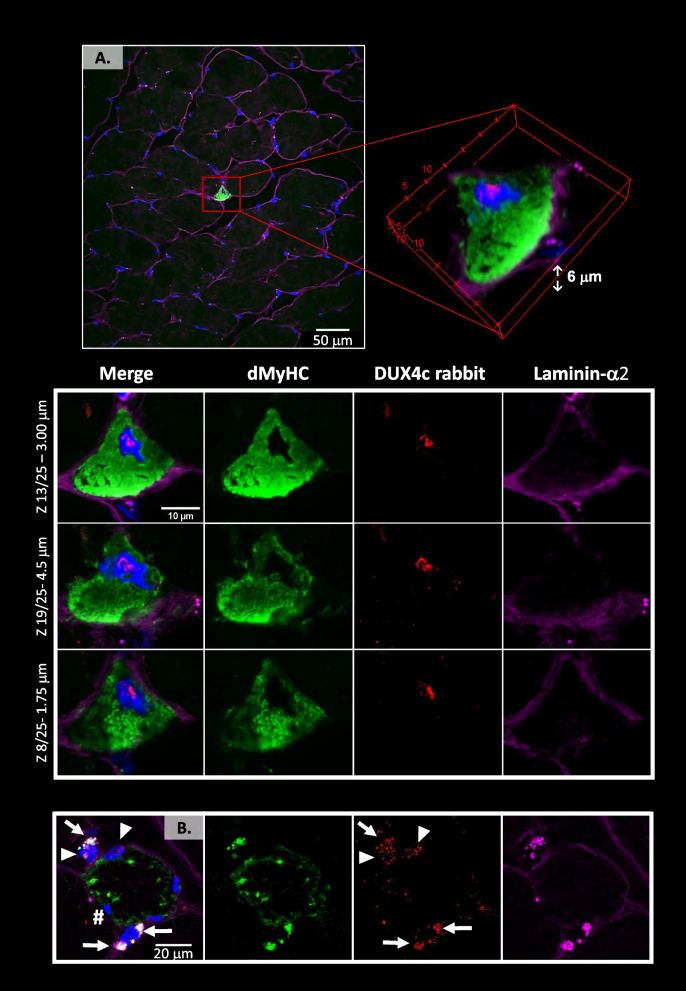
DUX4c is immunodetected in hypotrophic regenerating myofibers. The FSHD muscle sections were treated as in Fig. 3 with immunodetection of developmental myosin heavy chain (dMyHC, green), a regeneration marker, DUX4c (red) and laminin-α2 (purple), observed by confocal microscopy. dMyHC was detected in either (**A**) angular or (**B**) round hypotrophic fibers. **(A)** Upper panel: a muscle section area with one regenerating myofiber and its magnified 3D reconstruction. Bottom panels: three different focal depths of **A** using a 0.25 μm step in the *Z* axis (25 images in total). **B** Close to the round myofiber with punctuated dMyHC staining, two large nuclei present next to their periphery (at one or both sides) intense dots of dMyHC and of laminin-α2, suggesting they are included in activated satellite cells (SCs). DUX4c is detected in two close nuclei present in distinct cells (arrowhead), and also in the SC cytoplasm (arrows). An enlarged field of this cluster of regenerating cells is presented at Fig. S8A where the indicated nucleus (#) can be observed

As DUX4c staining was only sporadically found, we performed immunofluorescence in FSHD muscle sections from 16 additional patients including well-characterized patients and biopsies [31]. We combined DUX4c immunostaining with laminin-α2 detection to delimit the myofibers and with several regeneration markers (see next data section). In parallel, we performed histological coloration showing a higher connective tissue surface area (Fig. S5D) compared to healthy controls. We detected DUX4c in all patient muscles (Table S4) and observed an abnormal laminin-α2 staining, besides its classical location around muscle fibers. However, it is difficult to assert whether this was due to muscle cutting artefacts or to a real location. Indeed, myofibers presented either disruptions in the surrounding lamina with punctate laminin-α2 staining (yellow arrowheads) or its total absence in a large fiber part (yellow arrows) (Fig. S6). Nevertheless, we mostly found such defects either in areas presenting fibers (generally in clusters) with delocalized nuclei (DN, #) or that were hypotrophic (Fig. S6). We also observed locally an intense laminin staining (Fig. S6E (box)) and or ‘extra’ lamina inside fibers (Fig. 3B (box1)), C (yellow arrows)). Furthermore, in these areas, we noticed normal size myofibers with an unusual shape which presented one to several abnormal ‘extensions’ at their periphery like round or angular tips. These tips could include one or two myonuclei (Fig. 3B (box 2), C and Figures S6D and S7A). The DUX4c-positive myofibers had a small to normal size with either an angular, rectangular or flat morphology (Figs. 3 and 4, Figures S6, S7, S8 and S9). Some of these myofibers, containing one to several nuclei (dispersed or grouped in a cluster), presented a DUX4c staining either within the nuclei or at their periphery (Fig. S6B). Such a DUX4c staining in or near delocalized nuclei (DN, #) was found in sections from four biopsies (presenting 0.4 to 10% of fibers with DN, Table S4). We also sometimes observed a DUX4c signal inside the sarcoplasm either with a granular aspect (Fig. S6B) or as a line (Fig. 3 and Fig. S6C, D). Even though the rabbit anti-DUX4c serum gave a high background and stained many myofibers, a stronger signal was locally seen underneath the basal lamina of either hypotrophic fibers (Fig. 3A, B, Fig. S6E), fibers with DN or next to such fiber types (Fig. 3, Figures S6C–F, S7, and see below). Ten percent of the hypotrophic fibers presented one or several DN. Moreover, intense DUX4c staining was observed in areas that seemed at the periphery of a fiber missing a large part of laminin-α2 staining (Fig. S6E, F, white arrow). Peptide competition was performed as a negative control in parallel and never allowed the detection of such a staining (Fig. S6G). The DUX4c staining was mostly found in clusters of 4–5 myofibers (Table S4), more specifically in nearby regions inside 2 distinct fibers. For example, in Fig. 3 (each panel), the arrowheads point to a DUX4c staining near the membrane (that could be around a peripheral nucleus) that is very close to another DUX4c staining at the periphery of an adjacent fiber (either hypotrophic or presenting an unusual shape, white arrows). We observed a stronger DUX4c staining within or near the ‘abnormal’ tips. Some of these tips presented an intense laminin-α2 signal that appeared inside the myofiber (yellow arrows in Fig. 3C) suggesting an ongoing synthesis of laminin-α2.

In conclusion, DUX4c immunostaining was detected in rare myofibers of FSHD muscle sections. DUX4c appeared in nuclei as expected for a transcription factor, but also in the sarcoplasm or next to the sarcolemma, especially in myofibers that were either hypotrophic, of unusual shape or with delocalized nuclei. Myofibers with such features might result from incomplete regeneration processes in FSHD muscles.

### DUX4c co-detection with regeneration markers in skeletal muscle

The above immunostaining results underlined our earlier hypothesis [2, 3, 12] that DUX4c was expressed by regenerating myofibers. To specifically demonstrate this point here we immunodetected DUX4c and specific regeneration markers such as developmental myosin heavy chain (dMyHC), MYOD and CD56, using a confocal microscope. We also looked at desmin, a specific marker for myogenic differentiation, as we have previously reported its partial co-localization with DUX4c in FSHD and DMD muscle sections [12].

In the new biopsies analyzed in the present study, we found DUX4c-desmin co-detection in myofibers of at least five muscles (Table S4) either in very small muscle fibers (round or flat) (Fig. 3A, B, Fig. S7B) or around aligned and close nuclei at the periphery of a single fiber (Fig. S7C). We also observed fibers with delocalized nuclei (DN, #) presenting desmin staining at each tip that co-localized with DUX4c on one or both tips (Fig. 3A (box 2), Fig. S7D, E). Inside the fibers with an unusual shape, DUX4c partially co-localized with intense desmin staining (sometimes in co-detection with intense laminin-α2, yellow arrows in Fig. 3C). Globally, out of 600 myofibers delimited by laminin-α2 staining (from 5 patients), we detected DUX4c in ~ 10% of them (overestimation due to the non-arbitrary field selection), and half of them also presented intense desmin staining (in dots or larger area). Of all the myofibers, ~ 10% were hypotrophic and we detected DUX4c in ~ 60% of them. In addition, we observed a few myofibers that were very small, i.e., limited to a nucleus with a small sarcoplasmic area (<15 µm), that all presented DUX4c and desmin co-staining. A polarity in DUX4c staining such as the one found for desmin (Fig. S7E) could be observed, as previously reported in immortalized cells [12] or in primary muscle cells (see above).

Furthermore, we found DUX4c staining in all the dMyHC-positive fibers (that represented ~ 1.2% of the ~ 3000 analyzed myofibers in agreement with the percentage found by [32] in other FSHD muscles) either in nuclei (Fig. 4A, B) or next to them (Fig. 4B). Cytoplasmic DUX4c was observed at one or both fiber sides in partial co-localization with intense spots of both dMyHC and cytoplasmic laminin-α2 in very small cells (5 to 15 µm diameter) (Fig. 4B). Cytoplasmic laminin detection suggested these cells were activated satellite cells (SCs) or myogenic progenitors (MPs) that synthesize laminin before its deposition into the basal lamina [33]. Nuclear DUX4c staining was detected in these small cells but was mainly present in larger dMyHC-positive myofibers (25 to 85 µm diameter) (Fig. 4B). Apparently, ‘lobulated’ myofibers were probably fusing myofibers since dMyHC labeling was only present in one ‘lobule’ (Fig. S8A). Using a non-immune serum in place of the rabbit anti-DUX4c serum, we only observed a weak staining mainly outside myofibers or between clusters of regenerating myofibers (Fig. S8B, C).

**Fig. 5:**
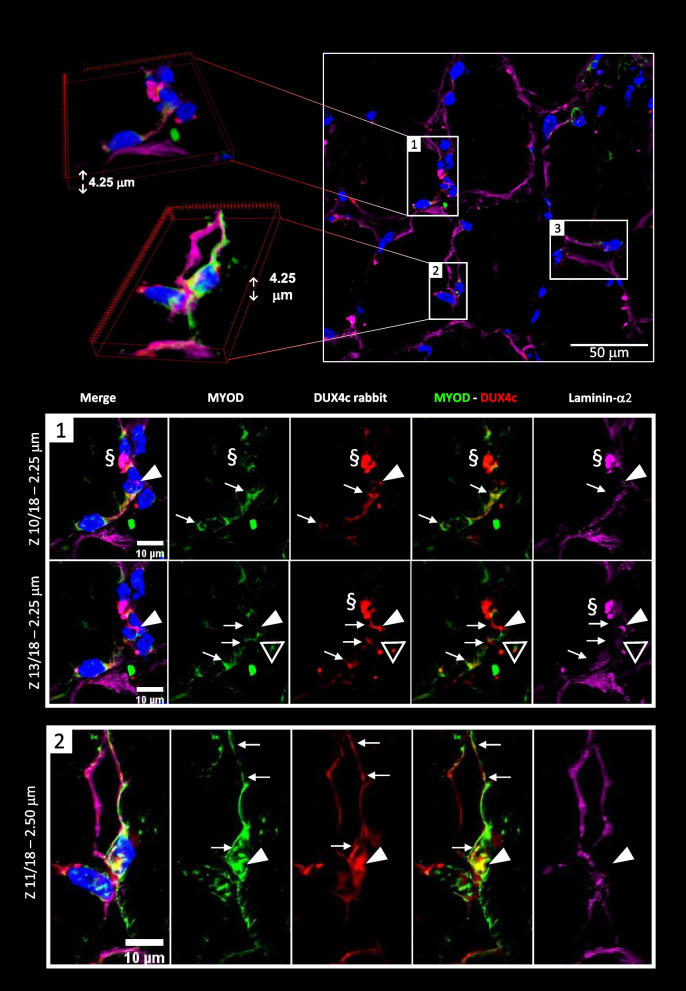
DUX4c is co-detected with MYOD, a myogenic regeneration marker. The FSHD muscle sections were treated and analyzed as in Fig. 3 with immunodetection of MYOD (green), DUX4c (red) and laminin-α2 (purple), observed by confocal microscopy. (Upper panel) A muscle section area with 3D reconstruction of two magnified regions. (Bottom panels) (Box 1) A muscle fiber tip with large nuclei surrounded by a MYOD staining with a partial DUX4c co-detection. Nuclear MYOD dots were also observed. Arrows point to the DUX4c labeling that is not co-localized with laminin-α2, supporting a cytoplasmic location. The triangle points to two dots, a DUX4c signal next to a MYOD one, at a nucleus periphery, without intense laminin-α2 staining. In contrast the area pointed with § shows co-detection of strong DUX4c and laminin-α2 signals between two nuclei. The arrowhead points to a DUX4c staining between two close nuclei (2 different focal depths using a 0.25-μm step in the *Z* axis, 18 images in total). (Box 2) MYOD detection in two close nuclei that belong to two distinct myofibers (separated by their respective laminin-α2 staining). The right one also presents a clear DUX4c nuclear signal. The arrowhead points to a partial nuclear MYOD/DUX4c co-detection. In addition, MYOD staining is observed around the nuclei and as a line just under the lamina of the right myofiber. DUX4c also shows similar staining in line with partial co-detection with MYOD. The image corresponds to a 0.25-µm section of the total 4.25 µm section depth

We also co-detected DUX4c with MYOD and found partial co-localization in both nuclei and cytoplasm. The MYOD cytoplasmic staining could extend as a long line under the myofiber periphery (Fig. 5, Fig. S9). The MYOD-positive cells were seldomly observed and generally involved several close nuclei at the periphery of adjacent myofibers with either an unusual shape, intense laminin-α2 staining or a double lamina (arrows in Fig. S9B), and a DUX4c staining, as described above. We commonly observed intense spots of laminin-α2 staining (as previously shown in Fig. 4B) that generally co-localized with an intense DUX4c staining next to MYOD detection (Fig. 5 (box 1)). It was not artefactual since we found identical intense DUX4c staining without laminin-α2 staining (Fig. 5 triangle, Fig. S9A, B). We also observed two nuclei that were very close but belonged to two distinct fibers at a cell–cell contact: one of them was positive for both DUX4c and MYOD, the other one was positive for MYOD with a DUX4c staining next to it (Fig. 5(box 2)). MYOD was also detected around two nearby nuclei surrounded by an incomplete and fuzzy laminin-α2 staining at a fiber periphery. Several dots of intense DUX4c staining were observed at one side of these nuclei (Fig. S9B).

Finally, we found DUX4c staining in CD56-positive cells around adjacent fibers close to myofibers with DN (Fig. S9C). Of note, the co-immunodetection was performed on several muscles with an inflammation score of zero (Table S4). We also noticed that beside its classical immunostaining in SCs, CD56 was detected in large homogeneous (Fig. S9D) or heterogeneous (Fig. S9E) cell clusters located between fibers presenting unusual shapes: these cells could be activated SCs as they were surrounded by a lamina (Fig. 9D). In addition, we co-immunolabeled the Ki67 proliferation marker with DUX4c in parallel in 7 FSHD and 3 DMD muscle sections. In DMD muscles, we co-detected Ki67 and DUX4c in the cytoplasm of grouped small cells. In contrast, we did not find such a co-staining in FSHD sections in the rare DUX4c-positive cells we detected (Fig. S10). Although Ki67 is nuclear in proliferating cancer cells, its cytoplasmic location was reported during muscle remodeling [34].

Altogether, these data suggested that DUX4c was expressed in activated SCs or MPs that could accumulate into clusters in FSHD muscles.

### Detection of DUX4, the causal FSHD protein, in regenerating myofibers

**Fig. 6:**
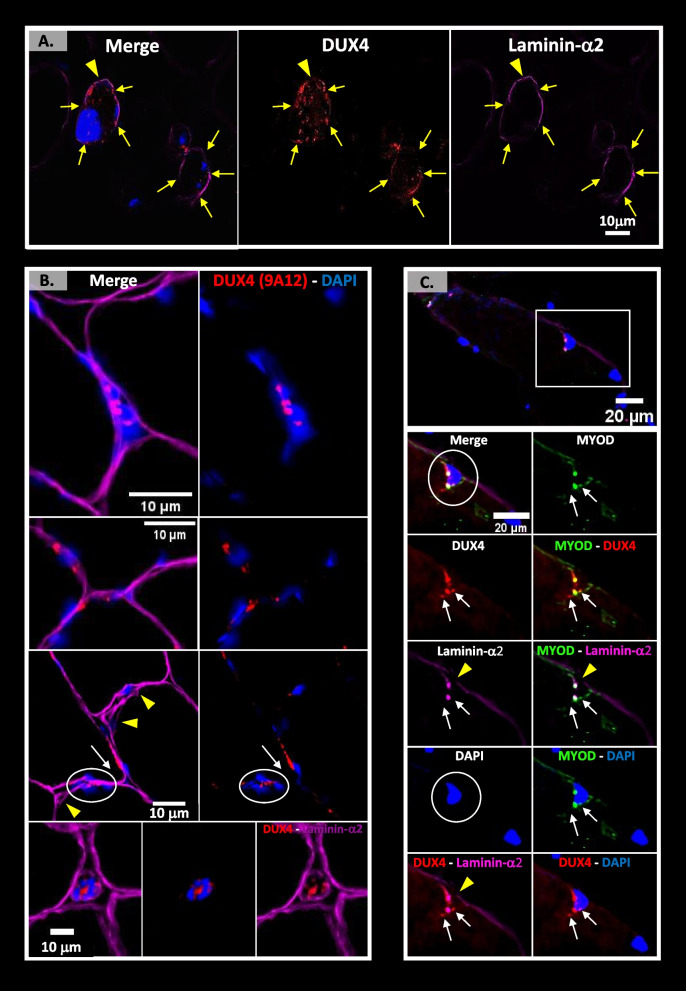
DUX4 and MYOD partially co-localize in hypotrophic FSHD fibers. The FSHD muscle sections were treated as in Fig. 3 with anti-DUX4 MAbs 9A12 (**A**, **B**) or E5-5 (**C**) (red) and anti-laminin-α2 serum (purple), observed by epifluorescence (**A**, **B**) or confocal microscopy (C). **A**, **B** 9A12 immunostaining reveals several dots either in the nuclei, sarcoplasm or at the fiber periphery of either hypotrophic fibers, some < 15 µm), that can be found in cluster (**A** and **B**: top and bottom panels), or normal-size fibers (**B**: middle panels). At the fiber periphery, 9A12 staining can be observed near/inside two close nuclei (or a cluster of them, circle) that are in 2 adjacent fibers. The arrow indicates an abnormal tip next to 9A12 staining. Large or punctuated laminin defects are pointed by yellow arrows or arrowhead, respectively, and some of their ‘ends’ correspond to a stronger DUX4 signal. **A** corresponds to magnification of a region indicated by a star in Fig. S11A. **C** Co-immunodetection of DUX4 (E5-5 MAb) and MYOD (5.8A MAb). Some rare DUX4 staining was found at the periphery of myofibers (confocal microscopy). DUX4 is partially co-detected in dots with MYOD and laminin-α2 around a large peripheral nucleus. However, two DUX4 dots (arrows) do not show laminin-α2 staining and the right dot partially co-localizes with MYOD

We have previously suggested that DUX4c could facilitate DUX4 toxicity by favoring clustering of myonuclei among which DUX4 could easily diffuse [3]. DUX4 only being expressed in rare cells, we took advantage of the large number of FSHD muscle biopsies available here (7 patients, see Table S4) and performed immunofluorescence with the mouse MAb 9A12 antibody we had raised against DUX4 [5] on sections adjacent to the ones used above for DUX4c. Globally, out of 400 myofibers delimited by laminin-α2 immunostaining, we observed 5% of DN and ~ 10% of hypotrophic fibers. We detected 9A12 staining (‘DUX4’) in ~ 20% of the hypotrophic fibers (overestimation due to the non-arbitrary field selection), half of which were very small (≤ 15 µm diameter), mostly in the sarcoplasm or at the fiber periphery (Table S4, Fig. 6A, B and Fig. S11A, B). Using the same epifluorescence microscope, we always observed 9A12 staining as small to large dots in contrast to the DUX4c staining that generally appeared as a line next to nuclei, in the sarcoplasm or under the lamina/sarcolemma. We also observed ~ 6% of normal size fibers with a ‘DUX4’ staining at their periphery, either near a nucleus and sometimes inside a large nucleus, or at the fiber periphery near laminin-α2 defects or at abnormal tips (Fig. 6B). Once, a ‘DUX4’ staining showed as two dots close to a central nucleus (Fig. S11C) (Table S4). ‘DUX4’-positive myofibers were generally grouped by 2–5 and either presented DN or an unusual shape, as described above, or were next to such fibers (Fig. 6 and Fig. S11A–C). We sometimes observed a fuzzy or larger laminin-α2 staining close to its ‘disruption’ point in such fibers that co-localized with a DUX4 intense staining (circle in Fig. S11C).

One could argue that because MAb 9A12 was raised against a peptide common to DUX4 and DUX4c (Fig. S2A), the similarity of MAb 9A12 and anti-DUX4c serum labeling could be due to DUX4c, not DUX4 detection. However, we have previously demonstrated by WB the specific DUX4 detection using MAb 9A12 on extracts of FSHD muscle biopsies [28]. In addition, the signals generated either by MAb 9A12 or the specific anti-DUX4c serum appeared with a distinct location in the same group of hypotrophic fibers observed in two adjacent sections (Fig. S11A and S7B, respectively) suggesting the epitopes targeted by these different antibodies were distinct. Yet, it might still be possible that MAb 9A12 recognized a DUX4c domain that would adopt another conformation or present different post-translational modifications than the one targeted by the rabbit anti-DUX4c serum. We therefore used the DUX4-specific rabbit MAb E5-5 (described in [35]) on some FSHD muscle sections and found a sarcoplasmic labeling around a large peripheral nucleus and around five close aligned nuclei (Fig. S11D). Even if the signal to noise ratio was low with MAb E5-5, the staining corresponded to the one we had observed with MAb 9A12, such as the one found around 3 close aligned nuclei (boxed in Fig. S11B).

Finally, to determine whether DUX4 was expressed in activated SCs/MPs, we performed MYOD-DUX4 (using MAb E5-5) co-immunofluorescence (~ 100 myofibers analyzed). At a confocal microscope, we saw a unique cell cluster with MYOD cytoplasmic labeling in which intense dots of MYOD, laminin-α2 and DUX4 staining partially co-localized (Fig. S11E), indicating these cells were activated SCs/MPs. This staining was not artefactual since some of these areas presented different intensities from one labeling to another. At the periphery of another myofiber, we observed co-immunofluorescence around two close nuclei partially surrounded by laminin-α2 stained as intense dots inside the fiber. These dots co-localized with intense DUX4 and MYOD staining, but in addition we could see faint DUX4 signals in the vicinity without laminin-α2 detection and one DUX4 signal without MYOD staining (arrows in Fig. 6C).

In summary, we could specifically detect the elusive DUX4 protein in FSHD muscle sections, in a cell cluster of activated SCs/MPs and at the periphery of myofibers, in partial co-immunolocalization with MYOD. Just like DUX4c, DUX4 was detected in regenerating myofibers.

### C1qBP is the major DUX4c protein partner

In order to start investigating a DUX4c role in muscle regeneration, we then wanted to study its protein partners. We had previously identified many protein partners shared between DUX4c and DUX4 [12]. As HEK293 cells expressed most of these partners and could be grown in large amounts we transfected these cells with expression vectors for DUX4c or EGFP proteins fused to a Halo-Tag. We then performed Halo-Tag-affinity purification (as described in [12], Fig. S12A), cleaved to peptides and analyzed them by mass spectrometry to identify and quantify the co-purified proteins [24]. The abundances of the proteins co-purified with DUX4c or EGFP were compared with Perseus [25] on six biological replicates, each with two technical replicates, for each condition (Fig. S12B and data not shown). This analysis pointed to C1qBP as the most significant DUX4c interactor while it was never found in any EGFP sample. In addition to C1qBP and other RNA-binding proteins (RBPs) we had previously identified as putative DUX4c partners [12], IMP1 was also more frequent in the DUX4c co-purification products, but its level was highly variable from one experiment to another not reaching statistical significance (Fig. S12B).

Using in situ proximity ligation assay (PLA), we found endogenous DUX4c-C1qBP interactions (red dots) in a few healthy and FSHD muscle cells, mainly in the cytoplasm, but not in the negative controls used in parallel (Fig. S12C).

### DUX4c-C1qBP interactions occurred in activated satellite cells/myogenic progenitors

**Fig. 7:**
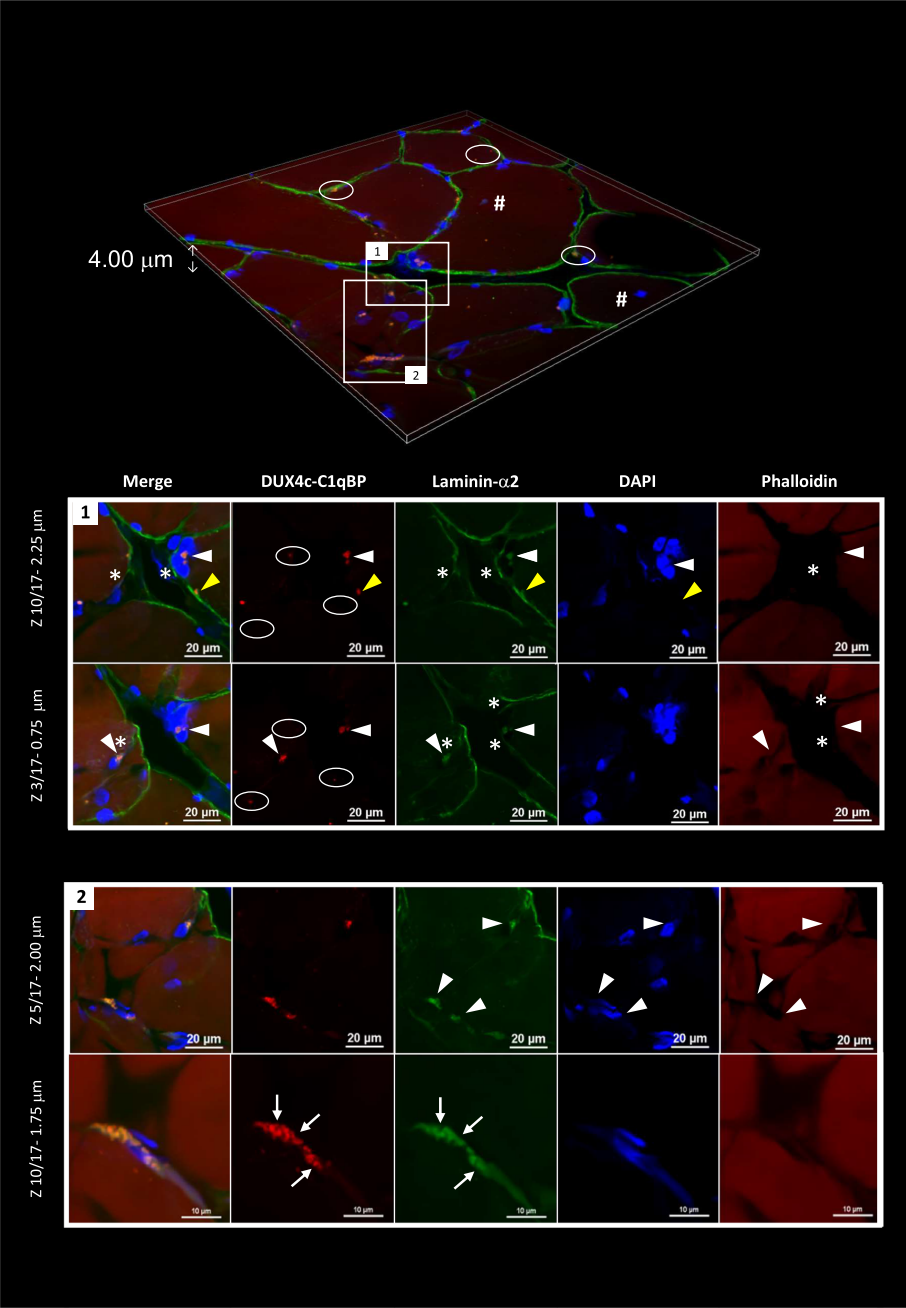
DUX4c interacts with C1qBP in FSHD myofibers. DUX4c and C1QBP interaction was determined by in situ proximity Ligation Assay (Duolink PLA) performed on fixed FSHD muscle sections (healthy control sections and negative controls are presented in Fig. S13A, B, C), using the rabbit anti-DUX4c serum and a mouse anti-C1QBP serum, followed by appropriate secondary antibodies coupled either to a plus- or a minus-DNA probe. If at 40-nm maximal distance both probes ligate, PLA signal can be amplified and detected by hybridization with a fluorochrome-coupled oligonucleotide, which corresponds to red dots. Laminin-α2 and the F-actin-binding phalloidin (to highlight the sarcoplasm) are detected with specific antisera, followed by secondary antisera coupled either with Alexa-488 (green, laminin-α2) or -647 (far red, F-actin), respectively. Staining was observed by confocal microscopy. (Upper panel) 3D reconstruction of a muscle section area with one myofiber containing a central nucleus (#). Boxes represent clear PLA signals with large dots in clusters and circles indicate the signals we have arbitrary set as nonspecific: dots not in a cluster on several *Z* axes (see example in box 1). (Bottom panels) Different focal depths or magnifications (17 images with steps of 0.25 μm in the *Z* axis). Box 1 corresponds to two distinct depths focusing on two specific PLA signals (arrowheads) either near a cluster of nuclei at a myofiber tip or at a single nucleus periphery. Both are in areas without phalloidin detection suggesting these nuclei belong to cells with a very small cytoplasm, as laminin-α2 staining is detected either surrounding the clustered nuclei or as a line inside a myofiber next the single nucleus, or is co-detected with the PLA signal (stars). The yellow arrowhead points to a PLA signal that presents a shape distinct from the surrounding laminin-α2 and might be localized at the tip of a satellite cell. Box 2 focusses on a region (two magnifications at two depths) where many PLA signals are found on both sides of an elongated nucleus with co-detection of intense laminin-α2 dots. This cell partially surrounds a myofiber and could be an activated satellite cell. Arrows point to distinct PLA signals and laminin-α2 dots. PLA was performed with the anti-DUX4c and-C1qBP primary antisera pairs on muscle sections from 4 patients with FSHD (in parallel to muscle sections from 3 healthy controls, see Fig. S13C)

To better characterize the cells with DUX4c-C1qBP interactions, we used PLA on muscle sections and found the larger and intense red dots at specific positions, generally next to nuclei, only in FSHD, not in healthy, muscles. DUX4c-C1qBP interactions were detected in fibers that presented DN or next to them (Fig. 7). The interactions (red dots in cluster) were found either between nuclei, forming a cluster at a myofiber tip, next to a single nucleus (box 1) or in activated SCs/MPs (in which laminin-α2 synthesis is ongoing) (box 2). Other smaller and less intense dots (arrows) could be detected inside or at the periphery of myofibers and corresponded to non-specific signals as they were also found in the negative controls used in parallel i.e. either an adjacent FSHD muscle section (either both rabbit and mouse IgGs in place of the two specific primary antisera, or preimmune serum combined with mouse IgGs, Fig. S13A, B, or competing peptide incubation before applying the specific primary antisera) or in healthy muscle sections (with both primary antisera) (Fig. S13C). Detection of the specific red dots (in cluster) occurred in very scarce areas in a single myofiber or bundled myofibers (Table S4) that presented typical feature(s) of regeneration.

### C1qBP also interacts with DUX4 in FSHD myofibers

**Fig. 8:**
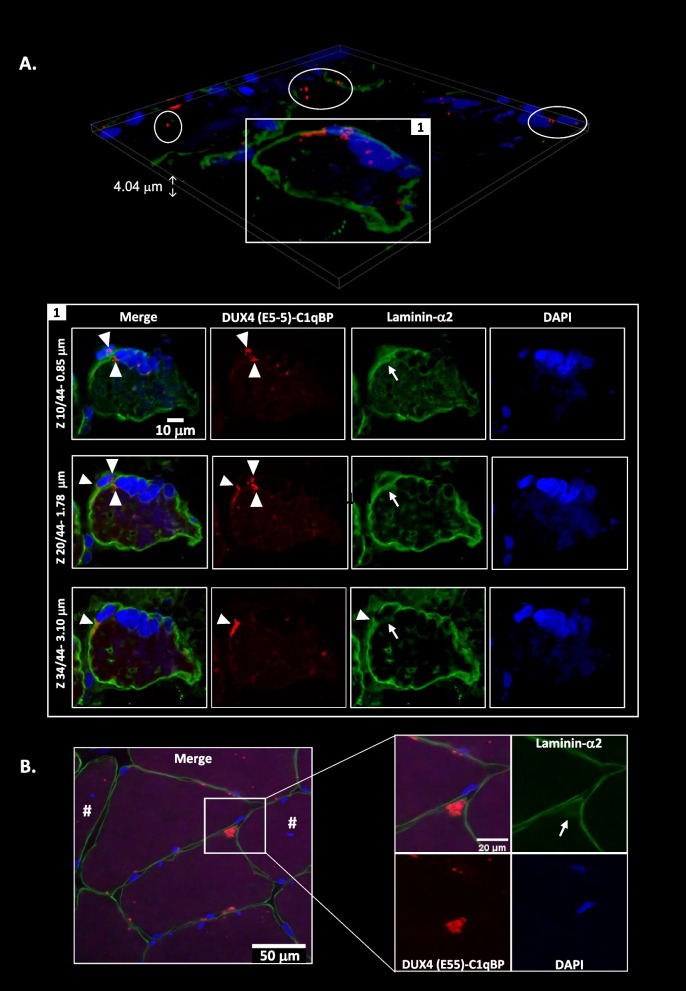
DUX4 interacts with C1qBP in a normal size myofiber with a cluster of nuclei and in a proximal close cell. C1QBP and DUX4 interaction was determined by PLA as described in Fig. 7 (healthy control sections and negative controls are presented in Fig. S14), using a mouse C1QBP antiserum and the rabbit anti-DUX4 E5-5 MAb. **A** (upper panel) 3D reconstruction of a muscle section area. Box 1 surrounds clear PLA signals with large dots in clusters and the circles indicate nonspecific signals (as determined in Fig. 7). **A** (bottom panels) Different focal depths (44 images with steps of 0.09 μm in the *Z* axis) showing PLA signal (arrowheads) in two close cells surrounded by laminin-α2 staining. The upper cell is small and could correspond to an activated satellite cell fusing with the below myofiber (arrow points to a lack of laminin-α2 only at a specific depth: images Z34/44). PLA signals are detected inside the nuclei (images Z10 and Z20) but also in the thin cytoplasm and in cluster at one nucleus side. Moreover, PLA signals are also observed next to the membrane and at the periphery of a very close nucleus residing inside the adjacent normal-size fiber (images Z10 and Z20), and next to the ‘fusion’ area (arrow). The myofiber present a very large cluster of nuclei, several are aligned and very close at the plasma membrane. **B** Cluster of PLA signals detected at a myofiber tip (triangular unusual shape) close to a myofiber having a central nucleus (#). The box is magnified for a better laminin-α2 detection at the fiber tip. A fuzzy laminin staining is also observed within the ‘triangular’ tip (arrow), overlapping with the PLA signals. PLA was performed with the specific anti-DUX4 and-C1qBP primary antisera pairs on muscle sections from 5 patients with FSHD (also see Fig. S13E-F) (in parallel to muscle sections from 3 healthy controls, see Fig. S14A)

Since we and other had previously confirmed that C1qBP was a DUX4 interactor [12, 19], we searched here for this interaction in FSHD muscle sections. We used either the mouse MAb 9A12 or rabbit MAb E5-5, respectively, with a rabbit or a mouse anti-C1qBP serum. We observed DUX4-C1qBP interactions with intense red dots only in the FSHD muscles (Fig. 8, Fig. S13D–F), not in the negative controls performed on healthy (Fig. S14A) or an adjacent FSHD (Fig. S14B) muscle section. Using MAb 9A12, we observed DUX4/4c interaction with C1qBP next to aligned adjacent nuclei at the periphery of a muscle cell and on one side of these nuclei (Fig. S13D). Using MAb E5-5, the specific DUX4-C1qBP interactions were similarly found next to a cluster of peripheral nuclei inside a myofiber close to another very small muscle cell (one nucleus surrounded by the lamina) (Fig. 8A). Both cells presented red dots and seemed to be fusing since a discontinuous laminin-α2 staining was found between them and could only be observed in a specific confocal *Z*-axis (arrow). PLA dots were mainly located on both sides at the cell–cell contact (arrowheads). Other interactions were also observed inside myofiber tip regions with unusually strong angular shapes (Fig. 8B, Fig. S13E, F). In such tip regions, we could sometimes observe nuclei, some larger and rounder in keeping with a regeneration process (Fig. S13F). Such a kind of labeling was never seen in the negative controls performed in parallel (Fig. S13G, S14). The clear PLA signals as red dot clusters were rare (Table S4) and generally found in or next to fibers that presented typical feature(s) of regeneration.

### DUX4c co-localizes with several RNA-binding proteins (RBPs) in a few FSHD muscle cells

**Fig. 9:**
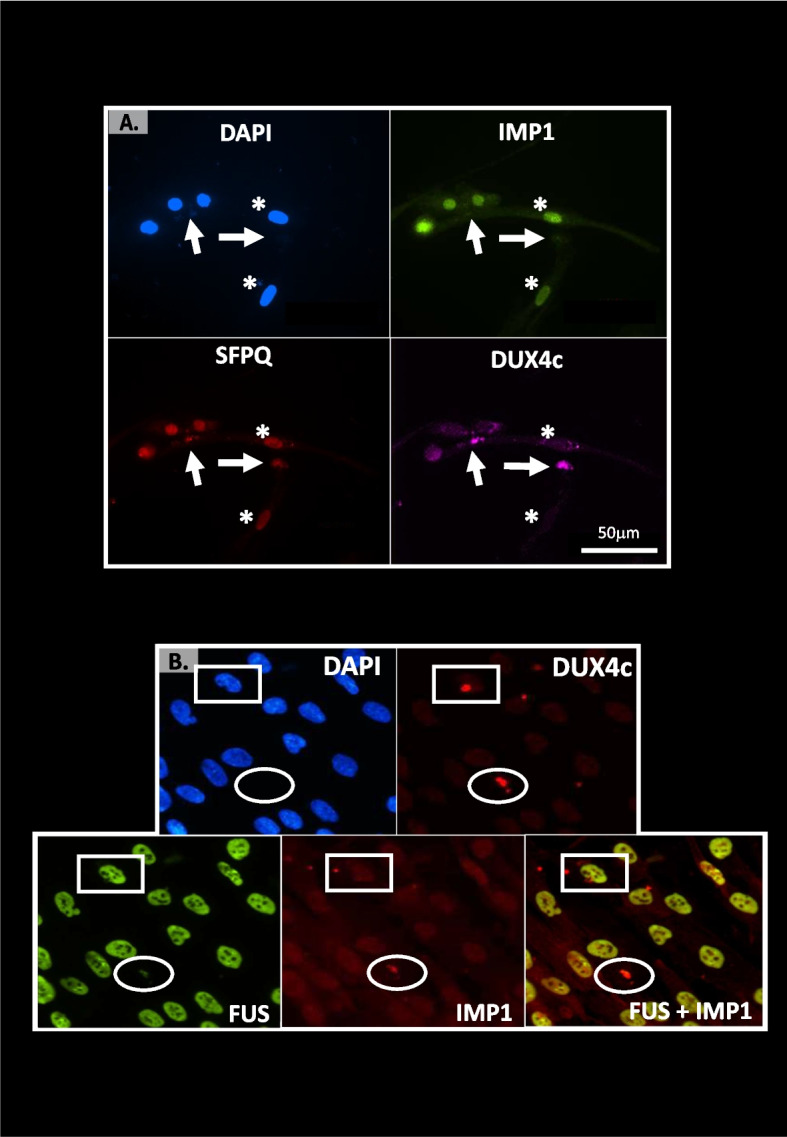
Partial co-localization of DUX4c with RNA-binding proteins IMP1, SFPQ, or FUS in FSHD muscle cells. FSHD primary myoblasts were fixed in PAF and immunofluorescence to detect DUX4c was performed as in Fig. 3 with the additional primary serum against either IMP1, SFPQ, or FUS and the appropriate secondary antibodies coupled to different Alexa Fluor with the indicated colors. Staining was observed by epifluorescence microscopy. The nuclei were labeled with DAPI. Partial cytoplasmic co-localization of DUX4c and the mRNP granule markers IMP1 and SFPQ (**A**, arrows) or IMP1 and FUS (**B**, circle). The arrows point to regions apparently at the tip of elongating cells (stars). Box shows an intense DUX4c staining without RBP co-detection. *N* = 3 biological replicates

We then wanted to investigate DUX4c interaction with other identified partners in FSHD myotube cultures. We have previously confirmed DUX4c RBP partners such as FUS (RNA-binding protein FUS) and SFPQ (splicing factor, proline-, and glutamine-rich) in cells transfected with a DUX4c expression vector [12]. Here, we confirmed partial co-localizations of endogenous DUX4c with SFPQ, FUS, or IMP1 (see above) in non-transfected FSHD myotubes. These co-localizations were found in the cytoplasm of very few cells, in line with the rare DUX4c cytoplasmic detection we had previously reported [12]. Several cells presented a triple co-detection of either DUX4c-IMP1 with SFPQ (Fig. 9, Fig. S15) or FUS (circle in Fig. 9B). However, on the 14 cultures performed in 8-well chamber slides and analyzed at different times during proliferation or differentiation, we only found 20 areas (× 20 or × 40 magnified fields) with such a labeling. These co-detections were close to myonuclei or clusters of myonuclei either as a large spot or in dots, and sometimes near cytoplasmic DAPI labeling that might correspond to mitochondrial DNA [36] (Fig. 9A). In FSHD myoblasts, such co-localizations also occurred in regions that seemed near cell–cell contacts (Fig. 9A), perhaps prior to fusion, as early myotubes were present (Fig. 2 and Fig. S3).

We also found DUX4c expression in testis as reported for DUX4 [35] and for other *DUX* genes at the RNA level (Table S3). We indeed detected DUX4c in some spermatocytes, late spermatids and spermatozoa (Fig. S16A). We also found partial co-localization of DUX4c with ILF3 or NF90 (interleukin enhancer-binding factor 3 or its alternative gene product nuclear factor 90) (Fig. S16B–D) that are RBPs involved in spermatogenesis [37] (see Additional file 1).

## Discussion

### Spliced *DUX4C* transcripts in primary muscle cells

We have previously detected *DUX4C* transcripts in primary human muscle cells [3]. We have now identified several *DUX4C* introns with reported splice consensus sites and 2 main RNA 3′ ends in FSHD and healthy muscle cells. Intron 2 had been described in muscle cells transfected with a *DUX4C* genomic construct comprising its own promoter [3].

The *DUX4C* mRNA might be regulated during early differentiation as we found that KLF15 (that physically associates with MyoD, [38]) affected *DUX4C* splicing in primary cells. Some alternative transcripts could be detected during a differentiation time course of immortalized muscle cells. However, they sporadically appeared with inter- and intra-variability in the several immortalized and primary cultures used (total of 17), suggesting, as for the protein (see below), a subtle regulation. Additional investigations are needed to determine whether specific isoforms are associated with muscle differentiation.

Together, our previous and current data demonstrate that *DUX4C* is an active gene. The gene showing the highest sequence identity with *DUX4C* is *DUX4L26* on chromosome 3. Like *DUX4C*, *DUX4L26* also presents a proximal enhancer-like signature in its 5′ part (ENCODE Accession: EH38E2215662). *DUX4L26* encodes DUXO (DUX of the organizer), a 243-residue protein that was detected in the nuclei of hES cells and was proposed as a regulator of the gastrula organizer in human embryonic stem cells [10]. A common transcription regulatory region might explain why the highest levels of both *DUX4C* and *L26* expression were found in brain cerebellum and testis (Table S3).

### The DUX4c protein is expressed in human differentiating muscle and in germline cells

In the present study, we have developed a new anti-DUX4c serum (rat) targeting a specific peptide that is different to the one previously used to raise the rabbit antiserum. The areas stained by these rat and rabbit sera partially co-localized in the same rare primary FSHD muscle cells. The cytoplasmic DUX4c fraction was only detected in elongating or fusing cells that expressed troponin T (TnT) or in ‘comet’-like cells showing a stronger DUX4c nuclear signal. Cytoplasmic DUX4c was mostly detected on one side of a cluster of nuclei or at the tip(s) of elongating muscle cells. DUX4c was immunostained with both antisera at cell–cell contacts, next to the membrane or to a cluster of nuclei and in the most intense TnT positive area, as previously observed with DUX4c ectopic overexpression [3]. As we had previously observed [12], we detected cytoplasmic DUX4c around the time of muscle cell fusion (a very quick event, [30]). We observed that the rabbit antiserum stained DUX4c in disorganized clusters of nuclei (less than 1% of all the analyzed nuclei) while the rat anti-serum only detected once a nuclear signal. It was in a single area composed of 3 nearby parallel myotubes with a normal morphology and aligned nuclei that were very close to each other (as found at cell fusion time) [30]. The diverse DUX4c intracellular locations found either using the rabbit, the rat or both antisera could be related to distinct DUX4c isoforms and might result from regulated post-translational modifications during the progress of differentiation.

Furthermore, DUX4c showed either no or variable detection in different muscles from a given patient, confirmed in 2 derived primary cultures, indicating that DUX4c level might be muscle-type dependent.

Lastly, in agreement with GTEx data (Table S3), we demonstrated that the DUX4c protein was expressed in testis, as previously shown for the homologous DUX4 protein [35]. Knopp et al. [14] proposed that both DUX4 and DUX4c target genes were involved in urogenital development. However, in contrast to DUX4, which was detected in spermatogonia and spermatocytes I [35], we found DUX4c staining generally located in differentiating germline cells, at the periphery of nuclei, extending to the cytoplasm. Moreover, in spermatocytes I, we observed co-localization of DUX4c with the ILF3/NF90 RBP at the nuclear periphery. We propose this might be linked to the regulation of mRNA fate since ILF3/NF90 were previously associated with RNA regulations [39].

### DUX4c protein detection in skeletal muscle is associated with muscle regeneration

In mature healthy skeletal muscle, the DUX4c protein is expressed at very low levels, and an amplification method has to be used for its immunodetection. DUX4c mRNA and protein were more easily detectable in FSHD muscle cells and sections (this study and [3, 12]). In the present study, we detected DUX4c at least in one muscle section from almost each FSHD muscle biopsy we analyzed and found nuclear or cytoplasmic staining, in keeping with our previous data in cell cultures and in a few muscle sections [3, 12]. DUX4c was always found in area containing myofibers with central/delocalized nuclei (DN) either at mild, moderate, or severe extent (according to [40]). Specifically, DUX4c labeling was generally found in bundles of small- to normal-size myofibers showing an angular, rectangular, flat or round shape. We have only taken pictures of these regions, therefore over-estimating the number of DUX4c-positive fibers. We determined that such fibers were in fact regenerating as we co-detected DUX4c with several regeneration markers such as the developmental myosin heavy chain (dMyHC), MYOD, CD56, and desmin (with the highest intensity). Our data support an active regeneration in FSHD muscles as recently described [32]. In all the FSHD muscle biopsies, we observed defects of the basal lamina (laminin-α2 immunostaining) independently of the clinical severity or of the muscle histology score. This might therefore reflect an early event in FSHD pathology. We believe these defects are not artefactual since they are mainly found next to myofibers with delocalized nuclei. Some intense or double laminin-α2 labeling was detected either inside or at the periphery of a myofiber and appeared either as a line or as cytoplasmic dots inside a cell with a unique large nucleus. The latter was also co-detected with dMyHC or DUX4c-C1qBP PLA dots inside cytoplasmic extensions partially surrounding a myofiber. These data support the fact that DUX4c-positive cells are either activated satellite cells (SCs) or another type of myogenic progenitor (MP). Laminin-α2 (as well as other laminin types) is induced during muscle regeneration and it could thus be stained as dots during protein synthesis in the cytoplasm before its secretion in the extracellular matrix [33].

Moreover, we also observed some areas between two adjacent muscle cells with missing lamina suggesting a recent fusion event. This was supported by the detection of “lobulated” myofibers presenting dMyHC in one ‘lobe” (that is in fact one cell) and a delocalized nucleus in another one without dMyHC staining. Furthermore, myofiber regions with delocalized nuclei also presented myofibers with an unusual shape (with one or more abnormal ‘extensions’ at their periphery like a round or angular tip) in which we could observe intense desmin and DUX4c staining. These tips could also contain a large nucleus or a cluster of nuclei that could be associated with discontinuous lamina. Finally, we also found a partial double lamina around MYOD and DUX4c co-detection. In conclusion, active regeneration was present in the majority of FSHD muscles analyzed.

It was proposed that SCs/MPs proliferate along the longitudinal axis in contact with a ghost fiber, i.e., the membrane left after a myofiber death [41]. We indeed observed such fibers in FSHD muscles with aligned and very close nuclei surrounded by an intense desmin and DUX4c staining at the fiber periphery, as previously seen in another FSHD muscle [12].

DUX4c staining could also be found with a pattern similar to the desmin one: polarized at one side or at a tip in immortalized or primary cell cultures ([3, 12], this study) but also in muscle sections (this study). DUX4c-desmin co-localization is in keeping with our previous study demonstrating desmin as a DUX4c protein partner [12]. We also found DUX4c next to the sarcolemma at cell–cell contact either inside peripheral nucleus or around it on both sides. MYOD was also present in such nuclei and in very thin long extension under the lamina. Our findings suggest that DUX4c is expressed early during the regeneration process as it is found concomitantly with either cytoplasmic or nuclear MYOD, the latter being essential for myoblast fusion. The membrane defects described in FSHD muscle cells and in myofibers of DUX4 mouse models [38] might be linked to fusion anomalies. Furthermore, both DUX4 and DUX4c overexpression negatively impacted myoblast fusion [14]. Future studies need to explore proteins involved in MYOD regulation in FSHD, such as (i) the MyoD family inhibitor (MDFIC) that maintains MyoD in the cytoplasm by masking its nuclear localization signal [42], (ii) the Id proteins that inhibit MYOD transcriptional activity [43], (iii) MYC that is reported to inhibit MyoD and muscle differentiation [44] and found stabilized by DUX4 ectopic expression [45], as well as other factors involved in muscle cell fusion. The fact that we found many activated SCs/MPs in several FSHD muscle types, as well as myofibers with an unusual shape, suggested that active regeneration in FSHD failed at one or several steps in the process although the succession of events leading to a complete regeneration is normally very fast [33, 41]. In agreement, clusters of unidentified cells were previously mentioned as a histological feature of FSHD muscles [46]. We found such clusters in some FSHD muscles that were surrounded by laminin-α2 with CD56 staining. It was reported that myoblasts migrating in the interstitial space did form clusters [47]. These cells might therefore be activated SCs blocked at a step of the regeneration process that could result from pathological modifications of their niche (fibrosis, inflammation, etc.) [31, 32]. We indeed detected early fibrosis in line with recent studies of FSHD biopsies [48, 49] and the very low chronic DUX4 expression model developed in mouse [50, 51]. and presenting pro-fibrotic alterations [52]. A recent study furthermore demonstrated that FSHD myoblasts stimulate collagen secretion by mesenchymal stem cells [53]. In patients, MRI combined to a proteomic study of muscle interstitial fluid suggested defective muscle regeneration and increased fibrosis in early/active FSHD [49]. Moreover, Banerji et al. [32] showed that the extent of fibrosis in FSHD muscles correlated with the proportion of fibers positive for dMyHC. To complete our knowledge on niche restructuration in FSHD muscles, additional components including soluble factors (laminins, collagens, fibronectin, prostaglandin E2, oncostatin M, etc.) [54, 55] need to be investigated. Our preliminary data indicate that most of the macrophages present in affected muscles were of the M2-type (Fig. S17B) that accumulate at sites of regeneration in dystrophic muscles [56]. Moreover, we also observed that Ki67-expressing cells in DMD were also positive for DUX4c, in agreement with its role in myoblast proliferation as determined by our gain- and loss-of-function studies [2, 3]. The fact that DUX4c-Ki67 co-localization was not found in FSHD contrarily to DMD muscles might suggest that the regeneration process is altered in FSHD. Indeed, myogenesis is perturbed in FSHD [57–59] and the muscle cell fate decision to proliferate or differentiate was proposed to be altered [17].

Altogether, our observations in FSHD muscles could correspond to delayed regeneration steps in which myoblast fusions might occur more slowly than usual. Laminin-α2 ‘defects’ we observed in such regenerating areas might correspond to remains of ghost fibers used as scaffolds for SCs/MPs as well as to differentiating MPs extending along degenerating fibers [41].

### *DUX4C* is an FSHD modifier gene

The chromatin remodeling at 4q35 in FSHD might impact *DUX4C* expression as suggested by its interaction by DNA looping with the D4Z4 array [60]. However, several observations suggested that DUX4c was not required to develop the pathology. Indeed FSHD could arise from chromosome 10q26 that lacks *DUX4C* if a PAS has been translocated distal of the repeat array and allowed for a stable DUX4 mRNA expression [61]. Moreover, *DUX4C* gene is deleted on the 4q35 permissive allele in some families with FSHD [62]. Nevertheless, as we previously mentioned in [2, 3, 12], *DUX4C* is still present on one 4q35 allele. Therefore, the increased DUX4c protein abundance in FSHD muscles we previously reported [2] might result from a transvection effect in which the activated permissive allele would induce *DUX4C* expression on the other allele. In addition, *DUX4L9* (corresponding to the *DUX4C* gene) is listed in the 228 most robust DUX4 targets in DUX4-overexpressing cells (two inducible cell lines) (Table 3 in [63]). Most of all, the observation that DUX4c gain-of-function induced disorganized myotubes with clusters of nuclei, accumulation of β-Catenin and delocalized α-Tubulin and Troponin T in vitro suggested it might impact muscle regeneration in vivo. Differentiation to such FSHD disorganized myotubes was only avoided by myoblast transfection with siRNA targeting DUX4c not DUX4 [16]. Moreover, during myogenic differentiation, the 4q35 chromatin undergoes dynamic changes [64]. Furthermore, the myogenic enhancers present at 4q35 and reported to regulate *DUX4* might regulate *DUX4c* as well [65] because we previously showed the primers used for 3C assay in that study targeted a sequence common to both genes (Figure S10 in [3]). We also have preliminary data showing that KLF15 (an activator of the *D4Z4* myogenic enhancer, [18]) impacts *DUX4C* splicing (see above). In agreement with a relationship between DUX4c and muscle regeneration, transcriptomic studies suggested that DUX4c, but not DUX4, was involved in muscle development by repressing genes such as *Hoxa1*, *Fzd2*, *Tnnc2*, *Myh7*, and *myoglobin* [14].

In conclusion, as DUX4c gain- and loss-of-function impacted either human myoblast proliferation, differentiation or function (myofibril and nuclear disorganizations) [3], it could affect any pathologic muscle. Furthermore, as DUX4c protein sequence, encompassing both homeodomains, is identical to a large part of DUX4, we could speculate that mis-expression of the FSHD causal protein in muscles might compete for normal DUX4c function in the regeneration process (see below). Indeed, we found both DUX4 and DUX4c in MPs. Competition of DUX4 with DUX4c normal functions when simultaneously expressed in identical muscle cells would be the reason why skeletal muscle is particularly sensitive to DUX4 toxicity.

### The DUX4 protein is detected in a few regenerating FSHD muscle fibers

We have previously immunodetected the DUX4 protein in nuclear or total extracts of FSHD muscle biopsies using western blots with MAb 9A12 and a very sensitive chemiluminescence detection procedure [5, 28]. The present study demonstrates DUX4 protein immunostaining in FSHD muscle sections. Even if MAb 9A12 was raised against a DUX4/4c common epitope, we have previously shown it was unable to detect endogenous DUX4c in FSHD muscle extracts by western blot (Figure S3 in [28]). Using MAb E5-5 specifically targeting DUX4, a labeling was found in rare cells, similarly to MAb 9A12 labeling that was found in fewer myofibers than DUX4c detected with the DUX4c-specific rabbit antisera in adjacent sections. Moreover, the staining pattern was different in the same cluster of double positive myofibers: MAb 9A12 epitope inside the myofiber around nuclei and DUX4c-specific epitope at the periphery. However, as observed in cell cultures using two distinct anti-DUX4c sera, we could not exclude that a few stealthy DUX4c isoform(s) might be detected by immunofluorescence using MAb 9A12.

Explanations as to why DUX4 is so difficult to detect in patient muscle could be provided by the extension to the whole tissue of data obtained in FSHD muscle cultures where its gene expression occurs as short bursts in very few myonuclei (1/1000 in myoblasts to 1/200 in myotubes); moreover the protein has a short half-life and its toxicity causes muscle cell death within 24–48 h [4, 5, 9, 66–69]. Similarly, we could detect DUX4 in just a few myofibers in muscle sections from 9 patients as recently shown in a single patient (biceps) by PLA designed to use 2 different antibodies targeting distinct DUX4 epitopes [70]. The images presented in the latter study identified DUX4 PLA dots in large nuclei that might correspond to MPs. In addition to nuclear PLA dots with combined P2G4 and E5-5 antibodies [68], DUX4 dots were also reported at the sarcolemma [70]. In our study, we found nuclear and cytoplasmic DUX4 in FSHD muscle cells using confocal microscopy with either MAb 9A12 or E5-5 used alone or in combination with an anti-C1qBP serum for PLA. Moreover, we found DUX4 near the sarcolemma either in partial co-detection with cytoplasmic MYOD or in desmin-positive cells, mainly in very small cells (< 15 µm), showing its mis-expression could occur at an early regeneration stage in agreement with its capacity to compete for PAX7 targets [71, 72]. The unexpected immunolocalization pattern might result from DUX4 cytoplasmic retention by interaction with a cytoplasmic protein such as desmin [12] or estrogen receptor β (ERβ) [73]. ERβ was found to be required for muscle regeneration and impacted the expression of extracellular matrix components, such as laminin and collagen [74]. By interacting with DUX4, ERβ could be involved in the laminin-α2 ‘defects’ we observed here. Moreover, the domain involved in nuclear hormone nuclear receptor interaction involved the specific C-terminal region of DUX4 [75] and might explain the difference in the detection observed between DUX4 (large dots inside the sarcoplasm) and DUX4c (‘line’ at the fiber periphery).

An additional complexity in FSHD pathological mechanism is the recent suggestion that DUX4 expression could mostly occur in inflammatory cells infiltrating patient muscles [76]. In the present study we have not observed DUX4 in non-muscle cells, but the muscle biopsies we have analyzed did not present strong inflammation.

### DUX4c protein partners are involved in muscle differentiation, repair, mass maintenance, and mitochondrial function

We found C1qBP was the major protein interactor of DUX4c. C1qBP is a constitutive multi-compartmental protein involved in several cellular functions such as the maintenance of oxidative phosphorylation and cell differentiation [77–79]. C1qBP has also been identified as an RBP [80]. Its function in skeletal muscle is not well established although its knockdown in sheep myoblasts inhibited their proliferation and differentiation and promoted apoptosis [81]. These observations underscore a role for DUX4c in muscle differentiation by its interaction with C1qBP. In agreement, the DUX4c-C1qBP interaction was found in MPs of FSHD muscle sections.

We have previously identified C1qBP among the DUX4 protein partners via its interaction with the homeodomains in a DNA-independent manner [12], and another study demonstrated C1qBP-DUX4 interaction in FSHD muscle cells [19]. Here, we have confirmed, as for DUX4c, DUX4-C1qBP interaction in MPs of FSHD muscle sections. These interactions were also observed facing each other on the sides of adjacent, probably fusing (as laminin-α2 staining was partly missing), myofibers.

In FSHD muscle cells (mostly in elongating or differentiating myoblasts), we showed here that endogenous DUX4c also interacted with other RBPs such as IMP1, FUS, and SFPQ. We had previously validated FUS and SFPQ as DUX4c partners in overexpression models and had found IMP1 as a putative partner [12]. IMP1 (also named IGF2 mRNA-binding protein 1, CRD-BP, VICKZ family member 1 or ZBP-1) is a protein involved in mRNA fate (nuclear export, protection from degradation, spatial and temporal translation regulation) [82–84] and its knockdown promotes myoblast proliferation and inhibits myotube formation [85]. IMP1 regulates actin synthesis following its phosphorylation at the membrane [86]. Similarly, such a regulation might be possible for DUX4c at the membrane where the IMP1-DUX4c co-localization occurred. It was also reported that a long non-coding RNA *lncMYOD*, expressed from a MYOD target gene and involved in myoblast differentiation, bound to both IMP1 and 2, and that *lncMYOD* knockdown up-regulated MYC [87]. Our earlier study pointed out that many DUX4 protein partners were involved in IMP1-dependent mRNP-granules [12] that regulate several specific mRNAs [84]. IMP2, that has a high sequence similarity with IMP1, is important for muscle repair and binds the *MYF5* mRNA to increase its translation [88]. We have previously found that DUX4c, but not DUX4, up-regulates the MYF5 protein [2]. In addition, we sometimes observed DUX4c-IMP1 co-localization with SFPQ or FUS in the cytoplasm. These proteins are also involved in mRNA fate [89] and are mainly known in axonal RNA transport [90]. Transcriptomic studies [14] also showed that DUX4c, but not DUX4, impacted expression of genes associated with axonal guidance. ILF3 and the associated NF90 isoform are also proteins associated with axonal RNA transport [91] (see Conclusion below). *Sfpq* knockdown induced progressive muscle mass reduction in the mouse [92]. FUS mislocalization is associated with mitochondrial abnormalities in skeletal muscle [93]. Ribonucleoparticles (RNPs) are highly dynamic structures controlling mRNA fate with frequent RBP exchanges [94] and their mis regulation is associated with skeletal muscle diseases [95]. Their precise regulations could explain why we only observed DUX4c-RBP interaction in very few cells. Transcriptome analyses (at global or single cell level) recently showed that the pathways affected in DUX4-positive FSHD muscle cells were mainly associated with mRNA fate [67, 96]. A proteomic study also pointed to the importance of post-transcriptional processes in DUX4-expressing cells [97].

In aggregate our data suggest that DUX4c interaction with RBP could have a function in muscle cell differentiation, specifically around the myoblast fusion stage. Increased levels of DUX4c or DUX4 proteins in FSHD muscle cells might interfere with normal functions of C1qBP or other RBPs and disturb the muscle regeneration process, thus aggravating the muscle pathology induced by DUX4.

## Conclusion

**Fig. 10:**
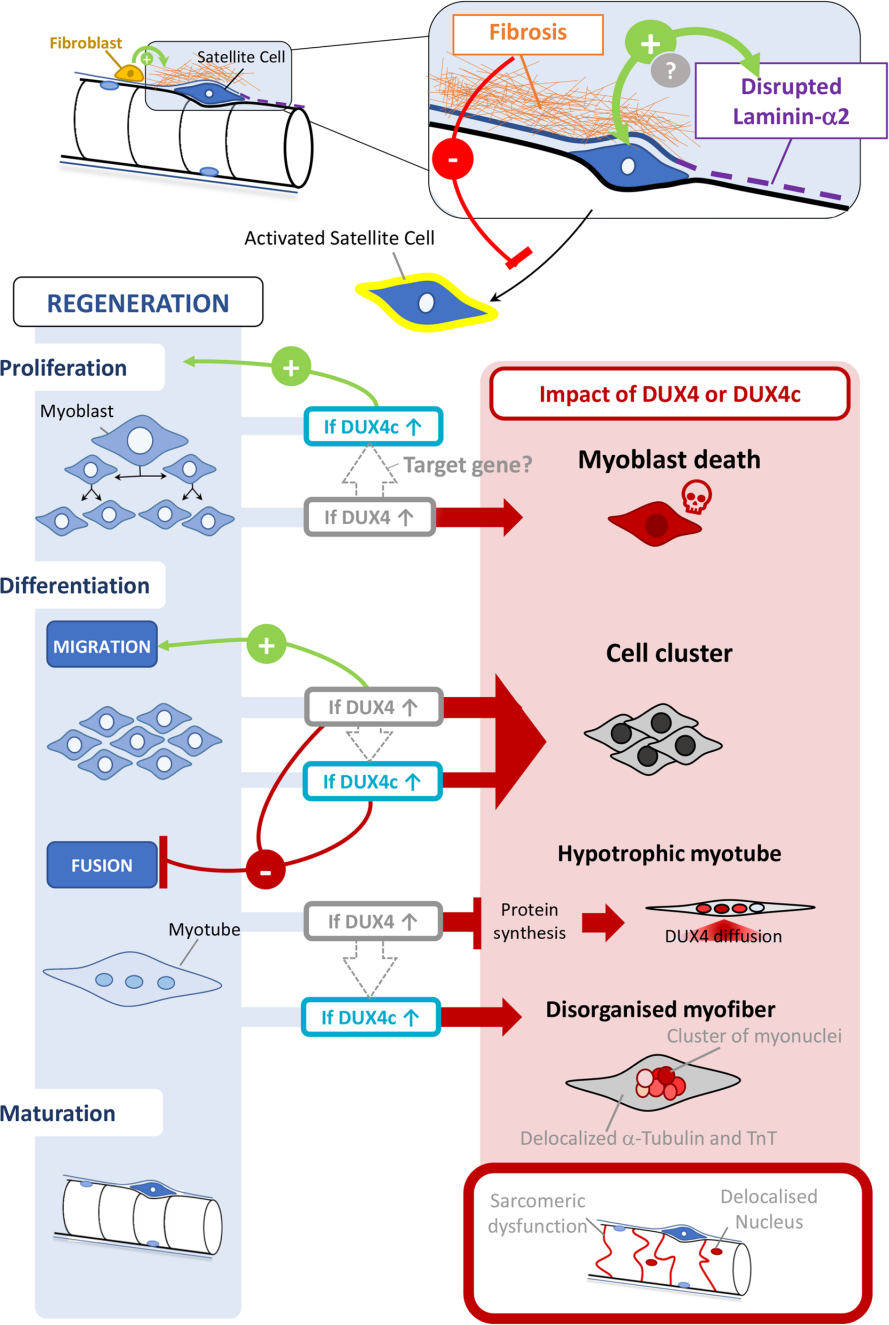
Proposed model on the impact of DUX4 mis- or DUX4c over-expression in FSHD muscles. (Upper panels) Punctate laminin-α2 disruptions or partial loss around myocytes/fibers are observed in all the FSHD muscles we analyzed including those with low clinical severity and histological score (Table S4). These laminin-α2 alterations might reflect basal membrane defects (as reported in FSHD, Figs. 10) that could either induce satellite cell (SC) activation or result from SC activation [100] (Figs. 10). We also observed cells containing intracellular laminin-α2 co-detected with either intense desmin (Fig. 3B, C), dMyHC (Fig. 4) or cytoplasmic MYOD staining (Fig. 5). These cells therefore correspond to activated SCs that are found next to specific myofiber(s) either hypotrophic, or with central nuclei or unusual shape, or several of these features. The fact we observed such cells could be that FSHD muscle cell fusion failed at some stage in the process as proposed for their classification as satellite cell-opathies [99]. Two features of FSHD muscles could affect the contribution of SCs to regeneration: DUX4 expression is known to inhibit myogenesis [30, 39, 101] and the extracellular matrix (ECM) thickening (Table S4, [31]) might affect the SC niche. (Bottom panels) Because DUX4c favors cell proliferation [2, 3], its overexpression would increase the myoblast proliferation rate. If DUX4 was expressed in proliferating SCs (myoblasts) or early myotubes, it would induce their death [4, 66, 67]. Myoblasts expressing DUX4 might have perturbed migration or increase the one of mesenchymal stem cells [67, 102]. Both DUX4- or DUX4c-overexpression negatively impact myoblast fusion [14]. Altogether, this might result in the formation of CD56- or MYOD-positive cell clusters corresponding to “frozen” satellite cells (blocked in differentiation) between myofibers (Figs. S9D, S11). DUX4 misexpression might perturb protein synthesis at the mRNA level ([97, 103, 104]) via its interaction with specific RBPs (major regulators of mRNA transport, translation and decay) and result in hypotrophic myotubes [3, 12] (Figs. 6 and 7, Fig. S11) in which DUX4 could diffuse among nuclei and thus expand its transcriptional deregulation cascade [9]. Moreover, DUX4 might compete with DUX4c for C1qBP binding since this interaction occurs via their identical homeodomains [12] and at similar intracellular locations next to clusters of large and close nuclei at the (ghost) myofiber periphery (Figs. 7 and 8, Fig. S13). DUX4c-overexpression in myotubes leads to the formation of disorganized myotubes presenting non-aligned nuclei (in clusters), that might favor DUX4 diffusion since they are closer to each other, as previously proposed [3]. Moreover, DUX4c-overexpression induced troponin T and α-tubulin delocalization, as well as β-catenin accumulation [3]. The latter is known to impact myogenesis [105] and to be a central coordinator of FSHD signaling pathways [106]. In mature fibers, Lassche et al. [107] reported sarcomeric dysfunction that might be associated to myofibril anomalies. Altogether, this would lead to non-functional myofibers and therefore SC activation. The higher proportion of M2 macrophages in FSHD muscle (Fig. S17B) is in favor of an active regeneration, as proposed by Banerji et al. [32]. However, early fibrosis ([31], Table S4) might interfere with a proper muscle regeneration. Moreover, Ki67 expression is not concomitant with DUX4c expression as is the case in DMD muscles (Fig. S10). In addition, other factors involved in muscle regeneration are deregulated by DUX4 misexpression (reviewed in [9]) and could further affect the myogenesis process. Finally, *DUX4C* (*DUX4L9*) might be a DUX4-target gene. Indeed, it was listed in the 228 most robust DUX4 targets (about a sixfold induction) [63]

In summary, our data underscore a functional role for the DUX4c protein by its interactions with several RNA-binding proteins, that are involved in muscle differentiation, repair and mass maintenance. As its homologue *DUX4*, the causal FSHD gene, *DUX4c* is also expressed in testis, but mainly in differentiating cells and in interaction with RBPs. DUX4c could have a broader function in cell differentiation since its major interactor C1qBP is a ubiquitous protein. DUX4c also interacts with other proteins known to be associated with axonal mRNA transport, whereas the GTEx database reported the highest *DUX4C* expression in brain cerebellum. Other tissues such as the pituitary, adrenal gland, esophagus and thyroid are also reported by GTEx to express *DUX4C* at weak levels. As several therapeutic strategies developed against FSHD focus on *DUX4* inhibition our study draws attention to the high sequence identity shared with *DUX4C* as well as *DUX4L26 (DUXO)*, the latter also detected at low level in skeletal muscle. Therefore, as previously mentioned [3, 12, 16], therapies for FSHD should avoid interference with the normal function of DUX4c or DUXO in adult tissues. Our data are in keeping with the active FSHD muscle regeneration recently shown by Banerji et al. [32, 72], and with a previous study suggesting that small angular fibers in FSHD mostly were the product of regeneration [98]. The present study has identified defects that may weaken the myofibers in FSHD muscles: (i) the absence of Ki67-positive proliferating cells, (ii) the formation of abnormal muscle cell clusters, (iii) a longer time for myoblast fusion, (iv) defective alignment of nuclei (that remain in clusters) in myofibers, and (v) abnormal myofibril organization. In addition, we also detected an increased population of M2-type macrophages in FSHD muscles as expected during an active regeneration but with an early fibrosis that might impair it. Altogether, our data agree with the recent proposition to consider FSHD as a satellite cell-opathy [99] and might explain why repression of PAX7-target gene signature is a superior FSHD marker than increase of DUX4 signature [71, 72]. Figure 10 summarizes our hypotheses on the impact of DUX4 mis-expression and DUX4c overexpression in FSHD muscles. However, additional studies are needed to determine whether DUX4c up-regulation observed in FSHD muscle biopsies [2] only reflects an attempt to regenerate.

As recently suggested [32], therapies targeting SC niche restoration or targeting of specific factors/pathways to improve muscle regeneration should be investigated in FSHD in combination with DUX4 suppression. In that context, we propose to further consider a decrease to a normal level (but not the suppression) of DUX4c protein, that our earlier and present data indicate is involved in muscle regeneration.

## Supplementary Information

**Additional file 1.** *DUX4c* protein detection in testis. DUX4c partial colocalization with ILF3/NF90 in testis.

**Additional file 2: Figure S1.** *DUX4C* mRNAs and protein in muscle cells. (**A-C**) Total RNAs were extracted, retro-transcribed and analyzed by 3’RACE with a *DUX4C*-specific primer as described in Methods. Samples of such 3’RACE products (see Table S2 for sequences) from RNAs of healthy or FSHD immortalized (**A**) or primary muscle cells (**B**) were analyzed by electrophoresis on agarose gels. P: proliferating myoblasts, C:confluent myoblasts, D: differentiating myoblasts (incubated either 1, 3, 7 or 9 days in adifferentiation medium). (**C**) *DUX4C* 3’RACE products of RNAs from primary muscle cells transfected with either an EGFP- or KLF15-expression vector. In parallel, *DUX4C* 3’RACE products of RNAs from C2C12 cells transfected with the 7.5 kb human genomic fragment comprising *DUX4C* (p7.5-kb) as described in (2). The arrows indicate the 1.2-kb product (intron 2 spliced out). Negative control: H2O in place of cDNA during the nested PCR. (**D**) Total (T), nuclear (N) and cytoplasmic (C) protein extracts of healthy or FSHD immortalized muscle cells were separated by SDS-PAGE transferred to a nitrocellulose membrane. The membrane was blocked and then incubated with rabbit anti-DUX4c serum followed by secondary antibodies coupled to HRP and revealed with the Super Signal West Femto maximum sensitivity substrate (see Material and Methods). C+ is the positive control i.e. an extract of cells transfected with pCIneo-DUX4C as described in (2).

**Additional file 3: Figure S2.** Validation of the rat anti-DUX4c serum. (A) Schematic alignment of DUX4, DUXO and DUX4c protein sequences. The percent identities indicated on vertical black lines correspond to the sequences aligned from the N terminus to this point. The percent value in red refers to the short sequence alignment in red. The specific DUX4c peptide sequences used for rabbit (2) or rat immunization are shown, as well as the regions targeted by the mouse 9A12 and the rabbit E5-5 monoclonal antibodies.raised against DUX4. (B) HEK293 cells were transfected with either *pCIneo*, *pCIneo-DUX4* or *pCIneo-DUX4c*. Twenty-four hours later, cells were harvested and total proteins were extracted, separated by SDS-PAGE and transferred to a nitrocellulose membrane for immunodetection with the mentioned primary antibodies as described in Fig. S1.

**Additional file 4: Figure S3.** Time-course of DUX4c expression in primary FSHD muscle cells. (**A**) Microscope images of enlarged fields presented in Figs. 2 or S3B (boxed) showing merged immunofluorescence of DUX4c with both rabbit (red) and rat (pink) antisera, Troponin T (TnT-green) and DAPI (blue). The yellow signal corresponds to intense TnT staining co-localized with DUX4c detection. Myotubes with a cluster of 3 to 10 nuclei and high DUX4c nuclear and cytoplasmic labeling are indicated (#): they appear like ‘comets’ and were scarcely observed. For the *Serratus Posterior Superior* (SPS) muscle cultures, the following numbers of microscopic fields were analyzed at the indicated times, 5 fields in proliferation (P), at days D1 and D3; and 4 fields at day D6. For the *Serratus Posterior Inferior* (SPI) muscle culture, 3 fields were analyzed in P and at day D6); 11 fields at D1 and 5 fields at D3. (**B**) Additional magnified regions presenting DUX4c staining as described in Fig. 2. Arrows point to DUX4c cytoplasmic labeling, and stars to DUX4c-negative nuclei. The strongest DUX4c nuclear staining was detected in TnT-expressing cells (arrowheads). The circle highlights cytoplasmic TnT accumulation that co-localized with DUX4c when it was detected by immunostaining with the rabbit but not the rat antiserum. The opposite was observed in Fig. 2 (at D1): TnT co-localized with DUX4c immunostaining observed with the rat but not the rabbit antiserum.

**Additional file 5: Figure S4.** DUX4c immunofluorescence intensity variation during primary myoblast differentiation time-course. (**A**-**B**) Representative pictures of DUX4c detection (red) with the same parameters for the image acquisition and processing at each time point:  DUX4c immunostaining intensity changed during the differentiation time-course and was culture-dependent: SPS (A) and SPI (B) primary muscle cell cultures. (**C**) The negative control used in parallel for the immunofluorescence with combined mouse, rat and rabbit pre-immune sera in place of the primary antibodies. Nuclei were stained with DAPI (blue).

**Additional file 6: Figure S5.** DUX4c detection by immunohistochemistry in muscle sections and histological alterations in FSHD muscles. (**A**) Immunostaining was performed with the rabbit serum raised against a DUX4c peptide (2) and secondary antibodies coupled to HRP on healthy muscle sections using the TSA amplification system with DAB detection. PAS counterstaining (pink) delimits muscle fibers, including satellite cells. Pictures were only taken in areas presenting DUX4c-positive nuclei (black arrows). Yellow arrows point to DUX4c-negative nuclei. (**B-C**) DUX4c immunostaining was performed on FSHD muscle sections as in (A) except a standard procedure was used with DAB detection and hemalun counterstaining (negative nuclei in blue, yellow arrows). The black arrows indicate strong DUX4c labeling in myonuclei at the periphery of fibers adjacent to an angular fiber (star, left panel) or presenting delocalized nuclei (right panel). Yellow arrows highlight DUX4c-negative nuclei. DUX4c staining could also present a granular aspect in the sarcoplasm (star) or extend from a peripheral nucleus to just under the basement or the sarcoplasmic membrane (arrowheads). (**C**) Sections of FSHD muscles from a single patient were treated (as in **Fig. 2C**) in parallel to detect DUX4c: either no (*infraspinatus*, IS, muscle) or variable DUX4c immunostaining from scarce (*sub-scapularis*, SS, muscle: in some nuclei at the periphery of a degenerating fiber) to several positive peripheral nuclei (*serratus posterior inferior*, SPI, and *intercostalis*, I, muscles). (**A-C**) Negative controls (boxed panel) correspond to either omission of the primary antibody or antigenic peptide competition on consecutive sections. (**D**) Muscle sections derived from patients presenting a CSS **<**5 (upper panels) or > 5 (middle and bottom panels) (characterized in **Table S4**) were stained with Heidenhain blue trichrome. The bottom panels present a cluster of hypotrophic myofibers with peripheral or delocalized nuclei (red arrowheads) and myofibril disorganization detected as branched or split sarcomeres (square) or presenting partial loss of sarcomeric regions (arrowhead). A degenerated muscle myofiber (in the center of the image) is characterized by acidophilic myofibrils (dark pink), vacuolar sarcoplasm and pyknotic delocalized nuclei. Further degeneration is shown by the presence of ghost fibers only detected by their empty basal lamina (PAS diastase stain).

**Additional file 7: Figure S6.** DUX4c and laminin-α2 detection by co-immunofluorescence in FSHD muscle sections. Immunofluorescence was performed on FSHD muscle sections as in **Fig. 3** using the primary rabbit anti-DUX4c and rat anti-laminin-a2 sera followed by appropriate secondary antibodies coupled to different Alexa Fluor molecules. Images were taken by an epifluorescence microscope. To better visualize DUX4c-specific staining, a higher image contrast was applied for panels C-F. (**A**) A rare cluster of small muscle cells (boxed, magnified to the right) next to myofibers presenting lamina defects such as either a large loss of the expected staining (yellow arrows), or a punctuated disruption, or a very thin staining (yellow arrowheads). Adjacent fibers present delocalized nuclei (#) and circles highlight areas with either intense laminin staining or a double lamina. (**B**) DUX4c detection in or next to delocalized nuclei (#) and in the sarcoplasm (granular aspect) in two adjacent angular fibers, similar to the one shown in **Fig.** **S5B** (star) and in our previous published data in a muscle section from another patient (*Figure 9* in Ansseau et al 2016). Of note, the image was taken in a region difficult to focus on. (**C-F**) Pictures taken on the same muscle section. In a few myofibers, DUX4c staining is detected as a ‘line’ in the sarcoplasm (arrowheads) of fibers presenting delocalized nuclei (#) and this DUX4c signal can be either between delocalized nuclei (C) or in the sarcoplasm with a faint laminin-α2 staining in the immediate vicinity (D). **(D)** Adjacent fibers also present delocalized nuclei (#). Arrows point to DUX4c-positive regions of these fibers or of a nearby hypotrophic fiber that present an unusual round or angular shape, sometimes around a nucleus and with a higher DUX4c staining just under the lamina. A very small fiber with a faint laminin-α2 staining ($) also presents intense DUX4c sarcoplasmic staining (arrowhead). (**E**) At proximity, very small myofibers (< 15 µm in at least onediameter) were found (star) with also a DUX4c staining under the lamina (detected either entire or partial), next to fibers with either delocalized nuclei (#) or lamina defect (yellow arrows) starting with an intense laminin-a2 staining (boxed) followed by a very long and thin extension (about 100 µm) ending in an intense DUX4c staining (white arrow). In this extension a double lamina (§) was observed around two nuclei (found in a cluster of 5 nuclei) as well as a DUX4c staining. (**F**) Partial DUX4c detection (arrowheads) at the periphery of another normal size myofiber (with delocalized nuclei and partial laminin-a2 staining) was found around a peripheral nucleus with staining extending under the lamina and that continues to another peripheral nucleus in the ‘missing’ laminin portion. A little further, a DUX4c staining (white arrow) was detected in the area where a laminin staining could be expected to complete the myofiber surrounding. (**G**) Negative control using competition with the antigenic peptide, the microscope green channel detection was boosted compared to the **A-F** pictures to allow visualization of a faint staining in a similar region presenting a normal size myofiber with delocalized nuclei.

**Additional file 8: Figure S7.** DUX4c-desmin co-detection by immunofluorescence in FSHD muscles. Immunofluorescence was performed on FSHD muscle sections as in **Fig. 3**. A background staining around all myofibers is observed with the rabbit anti-DUX4c serum (panels C-E). To better visualize DUX4c-specific staining, a higher image contrast was applied for panels A-B. (**A**) Enlarged picture of the area presented in **Fig. 3C** showing that the myofiber with the unusual triangular tip presenting DUX4c and desmin staining is surrounded by fibers with delocalized nuclei (#). (**B**) DUX4c was detected in aligned round hypotrophic fibers (numbered from 1 to 6) either in the sarcoplasm, sometimes as a ‘line’, (myofibers 1, 2, 4 and 6 that also present intense desmin staining) or at the myocyte periphery (myofibers 3 and 5) as also shown in a larger hypotrophic fiber (star). Adjacent fibers present DUX4c staining in some region of their periphery, and a more intense one around a nucleus (enlarged in the inset box). Of note, the image was taken in a region difficult to focus on; the adjacent section presenting the same hypotrophic fibers was used for DUX4 detection (**Fig** **S11A**) and confirmed laminin-a2 staining around myofiber 3. (**C**) Intense DUX4c (green) and intense desmin (red) co-staining around aligned nuclei (numbered from 1 to 7) at the periphery of a myofiber (surrounded by laminin-α2 staining, purple). (**D-E**) Partial DUX4c and desmin co-localization in putative regenerating muscle cells (arrowheads). In panel **E**, DUX4c presents the same staining ‘polarity’ (at one side of the hypotrophic fiber) than the intense desmin staining. Another hypotrophic fiber (star) with almost no desmin detection presents an a specific DUX4c staining at its periphery, near an adjacent normal size fiber with DUX4c-desmin co-detection at its periphery (arrow).

**Additional file 9: Figure S8.** DUX4c is immunodetected in regenerating myofibers. (**A**) Enlarged region of **Fig. 4B** with a 3D image reconstruction at the confocal microscope showing that the regenerating normal size myofiber (dMyHC-positive, green) seems to fuse with the adjacent fiber presenting a central nucleus (#, in top panel). (Bottom panel) 3D image reconstruction with an enhanced pink fluorescence detection shows a faint discontinuous laminin-a2 staining (suggesting a fusion event) next to a nucleus (pointed with #) that is thus not delocalized/central. The ‘ajdacent’ fiber with a central nucleus appears to extend at its other side to another fiber (see top panel) surrounded with a partial laminin-a2 staining (arrow). **(B-C) The negative control** is an adjacent section used in parallel with a non-immune rabbit serum instead of anti-DUX4c serum. Arrowheads point to the most intense negative staining found outside fibers or between regenerating hypotrophic fibers (corresponding to a lamina part). In addition, some intense stained laminin-a2 areas highlighted by the yellow arrows do not present any labeling with the non-immune rabbit serum.

**Additional file 10: Figure S9.** DUX4c co-detection with MYOD or CD56 as myogenic cell markers, and CD56 immunodetection pattern in FSHD muscle sections. Immunofluorescence was performed on FSHD muscle sections as in **Fig. 5.** (**A-B**) 3D reconstruction with a Z section showing merged and individual detections of DUX4c (red), MYOD (green) and laminin-a2 (purple). (**A**) Magnification of box 3 from **Fig. 5**. MYOD is immunodetected in some nuclear regions and in an area partially surrounding a peripheral nucleus where its signal extends from just under the lamina to the end of an intense laminin-a2 staining (circle). An adjacent fiber also presents a MYOD staining extending to part of its periphery (§). Several intense DUX4c signals were observed (arrowheads): next to or inside the nucleus in co-detection with MYOD; co-localized with an intense laminin-a2 staining (circle); and in another cell (left), next to a MYOD-positive nucleus either inside a nearby nucleus or next to a sarcoplasmic MYOD staining. (**B**) DUX4c detection around and inside two apparently bound nuclei at the fiber periphery: both nuclei are surrounded by MYOD staining but without DUX4c co-localization. The larger DUX4c positive area on one side (arrowhead) is surrounded by laminin-a2 staining that does not fully extend around the nuclei (§) suggesting these cells may be in the process of fusion. (**C**) Co-immunodetection of DUX4c (red) and CD56 (green) as a satellite/myogenic cell marker with respective specific monoclonal antibodies. DUX4c and CD56 co-localized at the periphery of adjacent myofibers (arrowheads). A myofiber with unusual shape and a delocalized nucleus (#) is observed nearby. The arrow points to a CD56 staining not associated with DUX4c labeling. (**D-E**) The CD56-positive cells are sometimes observed either (D) in small clusters between fibers or (E) in larger heterogenous cell clusters, where only some nuclei are next to CD56 labeling. Arrows point to abnormal tips in adjacent myofibers with an unusual shape.

**Additional file 11: Figure S10.** DUX4c co-detection with Ki67 proliferation marker in DMD and FSHD muscles. Immunofluorescence was performed on DMD or FSHD muscle sections as in** Fig. 5** with a monoclonal antibody against Ki67 instead of MYOD. (**A**) DUX4c-Ki67 co-labeling in DMD muscles. (**B-C**) No Ki67 staining is observed in the 7 FSHD muscle sections (**Table S4**). Rare DUX4c positive signals in putatively delocalized nuclei (longitudinal section, **B**) or near nuclei (transversal section, **C**).

**Additional file 12: Figure S11.** DUX4 detection in regenerating myofibers of FSHD muscles. (**A-C**) Immunofluorescence was performed on FSHD muscle sections as in **Fig S6** with the use of 9A12 (A-C) or E5-5 (D-E) monoclonal antibodies instead of anti-DUX4c serum. (**A**) Section adjacent to the one used in **Fig. S7B** showing desmin-positive aligned and round hypotrophic fibers, near a fiber with a central nucleus (#). 9A12 staining in dots (red) was detected around the nuclei or in the sarcoplasm of these fibers (numbered 1 to 7). Fiber 3 is surrounded by a laminin-a2 staining in contrast to **Fig. S7B **which only presents a peripheral DUX4c staining. Fiber 6 is missing in this section. The layer of myofibers of the section shown in **Fig. S7B** is shown by the dotted lines. The staining with 9A12 was distinct from the one observed with antisera against DUX4c in the same fibers, f.i., not in line or at the fiber periphery. Moreover, the adjacent myofibers (including the larger hypotrophic fiber pointed by *) are negative with 9A12 in contrast to the staining observed with anti-DUX4c serum. The arrowhead points to a myofiber with an intense DUX4c staining area around a nucleus in **Fig. S7B**. A nucleus is at a similar position in the present muscle section (inset). (**B**) DUX4 immunostaining is detected around 3 aligned nuclei localized in the longitudinal axis of a myofiber showing a shrunk region (boxed, magnified in the bottom panels) with faint laminin-α2 staining. Nearby myofibers present delocalized nuclei or an hypotrophic morphology. A yellow arrow points to another laminin defect in the same fiber. A weak DUX4 staining is also observed next to nuclei (arrowheads) or in hypotrophic fibers (stars): the bottom star is in a magnification of the region with DUX4 detection presented in **Fig. 6A**. The arrow points to a double lamina close to a peripheral nucleus. (**C**) Immunodetection with 9A12 mAb in normal size fibers with a central nucleus next to fibers with delocalized nuclei (#). Staining in dots is found either at the nuclear periphery or near the sarcolemma (arrowheads). The most intense labeling is co-detected with faint laminin-α2 staining next to its edge (circle). A yellow arrow points to another laminin defect in the same fiber. Arrows point to unusual tips or double lamina at the fiber periphery, sometimes around a nucleus. (**D**) Specific DUX4 detection using E5-5 MAb showing similar detection as above such as around 3 aligned and close nuclei in a fuzzy laminin staining area (as in panel B) or at the muscle periphery (as in panel C). (**E**) Specific DUX4 co-detection with sarcoplasmic MYOD in a cluster of myogenic cells with intense laminin-α2 staining, magnified in the right panels. (**F**) A competition with the DUX4 immunogenic protein domain (residues 190-424 coupled to a His-tag) was performed on a parallel section (negative control). The red channel signal was boosted compared to pictures A-F to allow the detection of a faint staining in a region presenting a hypotrophic fiber with a delocalized nucleus.

**Additional file 13: Figure S12.** C1qBP is the major DUX4c protein partner. **(A**) Purification of HaloTag-DUX4c protein complexes. HEK293 cells were transfected with *pHaloTag-DUX4c* expression vector. Cells were harvested 24 h later and lysed. The HaloTag protein complexes were then purified by affinity chromatography on Halo-Link resin and DUX4c protein complexes released by digestion with TEV protease to remove the Halo-Tag. Twenty-five μg proteins of the protein lysate (before chromatography, left) and the purified Halo-Tag complex (after TEV cleavage, right) were separated by SDS-PAGE followed by a Ponceau staining. After washing, DUX4c was immunodetected at the expected size either fused with the Halo-Tag (left) or after tag removal (right). (**B**) Volcano plot comparing the abundances of proteins co-purified with fusion proteins of DUX4c-or EGFP to Halo-Tag. The black curves mark the boundaries for a false detection rate of 1%. X-axis: the Log_2_ difference of abundances between the two conditions. Y-axis: P-value estimate for each protein. Proteins of interest are indicated in red. N=6. (**C)** C1QBP and DUX4/4c co-localization was detected by *in situ* Proximity Ligation Assay performed on healthy or FSHD immortalized myoblasts following fixation with PAF, using a mouse anti-C1QBP and a rabbit anti-DUX4/4c serum and appropriate secondary antibodies for PLA signal amplification (see Material and methods). Red dots correspond to these protein co-localizations. Negative controls used in parallel with non-immune mouse (Ms) and rabbit (Rb) sera in place of one or both primary antisera as indicated. N=3 biological replicates.

**Additional file 14: Figure S13.** PLA experiments in myofibers (negative controls and detection of DUX4-C1qBP interactions). (**A**-**C**) **Negative PLA controls for interactions between C1qBP and DUX4c. **3D reconstruction and examples of a Z-axis image for each negative control done in parallel with the PLA procedure of **Fig. 7**. In place of the specific primary antiserum pair used in **Fig. 7**, we incubated adjacent FSHD muscle sections with either mouse (Ms) and rabbit (Rb) non-immune sera (A) or the rabbit pre-immune serum with mouse IgGs (B). We also used healthy muscle sections with the pair of rabbit anti-DUX4c and mouse anti-C1qBP sera (C). No PLA dots in large cluster were found in these sections, only a specific staining was observed in the lamina. Pictures were taken with the same parameters for all the PLA reactions in areas with either hypotrophic fibers (A) or fibers with delocalized nuclei (B). PLA was performed with the non-immune serum pair on muscle sections from 3 patients with FSHD (and 1 healthy control, not shown), and the pre-immune serum combined with Ms IgGs on the FSHD muscle section. Another FSHD muscle section was used for PLA with the primary antiserum pair after competition with the DUX4c immunogenic peptide (pictures similar to A-B were observed, not shown). The pair of anti-DUX4c and anti-C1qBP primary antisera was used on 3 healthy muscle sections. **(D-F)** **DUX4 interacts with C1qBP in FSHD myofibers. **(**D**) Same procedure as above except that the primary antibodies used for PLA are the mouse 9A12 mAb and a rabbit anti-C1qBP serum. The only PLA dots we take into account are the ones in clusters (arrowheads). Such a cluster was found in a myofiber presenting delocalized nuclei, next to very closely aligned nuclei at the fiber periphery. They all seem surrounded by a lamina. Arrows point to an incomplete laminin-a2 staining region inside this ‘fiber’ that coincide with flat nuclei suggesting they are peripheral ones and that this ‘fiber’ is in fact in a fusion process. (Bottom right panels) Magnification of the boxed area in the upper merged picture. Some intense laminin-α2 dots are co-localized with the PLA dots (arrowhead), suggesting laminin-α2 is being synthesized there before extracellular secretion. Therefore, aligned nuclei could correspond to activated satellite cells or myogenic precursors. An FSHD muscle section was used for PLA with the 9A12/anti-C1qBP serum pair. (**E-F**) Same procedure as above except that the primary antibodies used for PLA are the rabbit E5-5 mAb and a rabbit anti-C1qBP serum on FSHD muscle sections (n=4). (**E**) Enlarged region of **Fig. 8B** with a 3D reconstruction showing a cluster of PLA dots in a tip with an unusual shape, adjacent to several fibers with delocalized nuclei (#). A focal depth different from the one presented in **Fig. 8B** allows a better detection of a fuzzy laminin-a2 staining in this tip area, suggesting that the tip is in fact a myogenic progenitor. (**F**) Focus in an FSHD muscle section on a myofiber with a delocalized nucleus (#) and an unusual shape with an angular tip showing both a laminin-a2 defect (yellow arrow) and areas of PLA dots in cluster (the larger ones in the 3D reconstruction), each next to a nucleus: one of them is larger and rounder, in accordance with a regeneration process at the abnormal tip.

**Additional file 15: Figure S14.** Negative PLA controls in healthy and FSHD myofibers. Negative controls used in parallel to sections of **Fig. S13E-F**. 3D reconstruction and an example of a Z-axis image for each negative control. (**A**) PLA with the pair of E5-5 (anti-DUX4 MAb) and anti-C1qBP serum on healthy control muscle sections (n=3). (**B**) PLA with combined mouse and rabbit IgGs in place of the primary antibody pair (n=2). Pictures were taken with the same parameters as in Fig. S13E-F and show, in the FSHD muscle section, myofibers with a delocalized nucleus (#) or an unusual shape with laminin defect (inset enlarged in the bottom panels). No PLA dots in large cluster were found. The circle in the 3D reconstruction points to nonspecific PLA signals in the lamina. Same experiment as** in Fig. 7 **except that the myoblasts had been cultured in a differentiation medium for 1- (**A-B**, n=3) or 3-days (**C-D**, n=3). Arrows or circle point to cytoplasmic partial co-localizations of DUX4c with the indicated RNA binding proteins.

**Additional file 16: Figure S15.** Partial co-localization of DUX4c with RNA binding proteins in differentiating muscle cells. Human testis sections were used. (**A**) Immunohistochemistry was performed as in **Fig. S5B**. The small boxed areas on the images are shown below at higher magnification. (Sg: Spermatogonia; Ser: Sertoli cells; SpI: spermatocytes I; St(1): early spermatids; St(2) late spermatids; Sz: spermatozoa). Arrows and arrowheads indicate DUX4c localization. (**B-D**) Immunofluorescence was performed as in **Figs. 3**: DUX4c (green) and ILF3/NF90 (red) were detected with specific primary antisera (see Methods) and appropriate secondary antibodies coupled to Alexa Fluor 488 or 555 respectively. (**B**) The boxed regions correspond to DUX4c/ILF3 partial co-localization. The star indicates a large spermatocyte I nucleus with DUX4c- and ILF3-positive nuclear spots. (**C**) Arrows indicate DUX4c/ILF3 partial co-localization at the nuclear periphery of a round spermatid (higher magnification of the boxed region in C in another Z axis image.) (**D**) Elongating spermatids (arrowheads) showing ILF3 cytoplasmic labeling. The arrow points to diffuse and weak cytoplasmic DUX4c staining.

**Additional file 17: Figure S16.** DUX4c immunodetection and partial co-localization with the RNA-binding protein ILF3/NF90 in testis. (**A**) **Detection of slow and fast myosin.** Immunofluorescence was performed on muscle sections with specific mAbs for slow and fast myosin (as described in Methods). In healthy control muscles, slow- and fast-twitch fibers present similar diameters. In the FSHD affected muscle analyzed, many slow fibers are atrophic (arrow) or necrotic (arrowhead). Ghost fibers or adipocytes are detected by their lack of myosin labeling (stars). (**B**) Co-immunofluorescence labeling of macrophage CD206/CD68 markers and laminin-α2 in affected muscles. Nuclei are labeled with DAPI. Few CD68+ (pro-inflammatory M1 macrophages) cells are observed (white arrows, upper panel). CD68^+^/CD206^+^ cells corresponding to M2 macrophages (yellow arrows) are much more frequent in the analyzed FSHD muscles (n=5). All macrophages observed inside FSHD muscle fibers are of M2 type (star, bottom panel). The histogram represents the percentage of M1 and M2 macrophages evaluated by counting CD68^+^/CD206^-^ and CD68^+^/CD206^+^ cells on 10 microscopic fields.

**Additional file 18: Figure S17.** Myosin and macrophages detection in FSHD muscles. (**A**) Detection of slow and fast myosin. Immunofluorescence was performed on muscle sections with specific mAbs for slow and fast myosin (as described in Methods). In healthy control muscles, slow- and fast-twitch fibers present similar diameters. In the FSHD affected muscle analyzed, many slow fibers are atrophic (arrow) or necrotic (arrowhead). Ghost fibers or adipocytes are detected by their lack of myosin labeling (stars). (**B**) Co-immunofluorescence labeling of macrophage CD206/CD68 markers and laminin-α2 in affected muscles. Nuclei are labeled with DAPI. Few CD68^+^ (pro-inflammatory M1 macrophages) cells are observed (white arrows, upper panel). CD68^+^/CD206^+^ cells corresponding to M2 macrophages (yellow arrows) are much more frequent in the analyzed FSHD muscles (n=5). All macrophages observed inside FSHD muscle fibers are of M2 type (star, bottom panel). The histogram represents the percentage of M1 and M2 macrophages evaluated by counting CD68^+^/CD206- and CD68^+^/CD206^+^ cells on 10 microscopic fields.

**Additional file 19: Table S1.** Primary antibodies. **Table S2.** Endogenous *DUX4C* mRNA sequences. **Table S3.** GTEx data on human *DUX* gene expression. **Table S4.** Clinical and histological features of patients and muscles, including patterns of DUX4c and DUX4 staining, their co-immunodetection with regeneration markers and their interaction with C1qBP (PLA).

## Data Availability

All data generated or analyzed during this study are included in this published article (and its additional files). The rat DUX4c antisera generated in this study are limited material that could be produced commercially based on the provided sequences.

## Abbreviations

C1qBP: Complement component 1 Q subcomponent-binding protein
DMD: Duchenne muscular dystrophy
DN: Central/delocalized nuclei
*DUX4C*: The double homeobox 4 centromeric gene/transcripts
DUX4c: The double homeobox 4 centromeric protein
*DUX4L9*: DUX4-like 9 gene
DUXO: DUX of the Organizer
FSHD: Facioscapulohumeral muscular dystrophy
ILF3/NF90: Interleukin enhancer binding factor 3/nuclear factor 90
IMP1: IGF2 mRNA-binding protein 1
MPs: Myogenic progenitors
RBPs: RNA-binding proteins
SCs: Satellite cells
