# Supplementary material for: The double homeodomain protein DUX4c is associated with regenerating muscle fibers and RNA-binding proteins
Source: Skelet Muscle. 2023 Mar 7;13:5. doi: 10.1186/s13395-022-00310-y (PMC9990282; doi:10.1186/s13395-022-00310-y)
Supplement: Supplementary file 1 — Additional file 1. DUX4c protein detection in testis. DUX4c partial colocalization with ILF3/NF90 in testis. [file 13395_2022_310_MOESM1_ESM.docx]

**Additional file 1**

**DUX4c protein detection in testis**

The DUX4c paralogues with the highest sequence identity (**Fig. S2A**) were previously detected in testis: these were DUX4 protein (79) and *DUX4L26* RNA (**Table S2**). We therefore investigated DUX4c protein expression in this tissue. DUX4c was immunodetected in some spermatocytes (large round nucleus) as well as in early (round nuclei) and late (triangular nuclei) spermatids and spermatozoa (**Fig. S16A**). In spermatocytes I, DUX4c labeling was heterogenous, from diffuse nuclear staining to specific spots on or at the nuclear periphery, or in the cytoplasm (arrows). A few early spermatids exhibited diffuse cytoplasmic labeling extending from one to two opposite spots at the nuclear periphery. In late spermatids, DUX4c labeling was generally observed in the nucleus vicinity, but on the side opposite to the acrosome position, and was sometimes detected as double spots (**Fig.** **S16A**, arrowheads). Sertoli cells and spermatogonia were unlabeled.

**DUX4c partial co-localization with ILF3/NF90 in testis**

ILF3 and NF90 (Interleukin enhancer binding factor 3 or its alternative gene product Nuclear Factor 90) are RBPs expressed in many cell types including germ cells. Because we detected DUX4c in testis and had previously identified a putative interaction with ILF3/NF90 in muscle cells (Ansseau et al 2016), we performed a co-immunofluorescence staining in testis. In spermatocytes I (star in **Fig. S16B**) showing specific DUX4c nuclear spots and in adjacent early spermatids (smaller nuclei), we observed partial co-localization of ILF3/NF90 (higher intensity) with DUX4c spots (lower intensity) (**Fig. S16B** boxes). Regions with stronger DUX4c staining did not show ILF3/NF90 co-localization, except at the nuclear periphery in a few cells (arrows in **Fig. S16C**). Cytoplasmic ILF3/NF90 was observed in elongating spermatids (arrowheads in **Fig. S16D**). Discrete spots of cytoplasmic DUX4c staining of weak intensity could be observed in these cells (arrows).
