# Supplementary figures and images for: The double homeodomain protein DUX4c is associated with regenerating muscle fibers and RNA-binding proteins
Source: Skelet Muscle. 2023 Mar 7;13:5. doi: 10.1186/s13395-022-00310-y (PMC9990282; doi:10.1186/s13395-022-00310-y)

Fig. S1

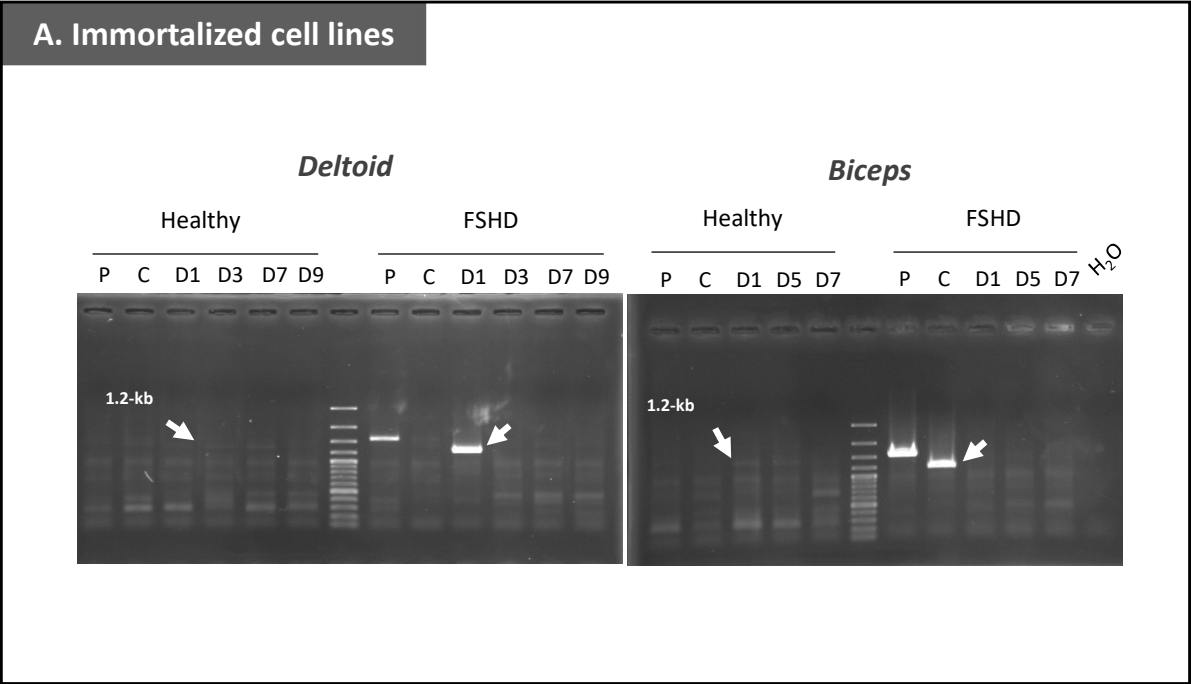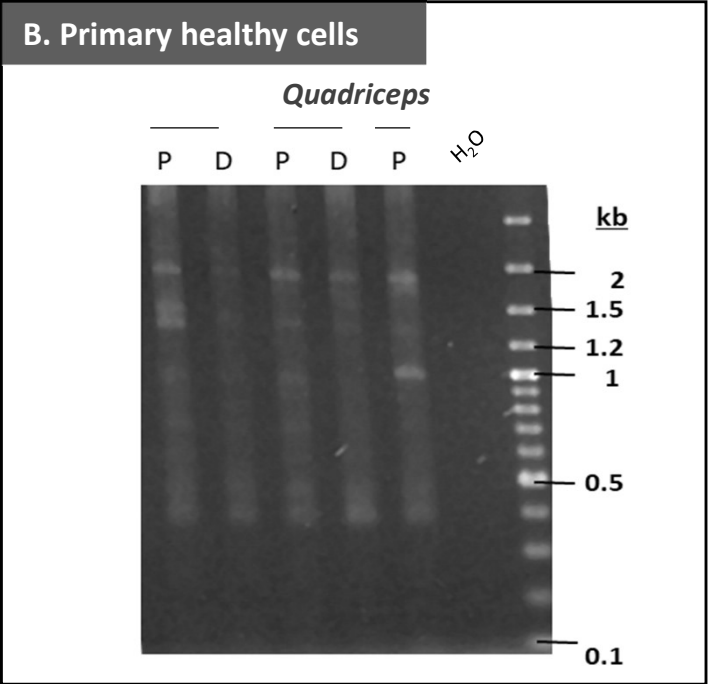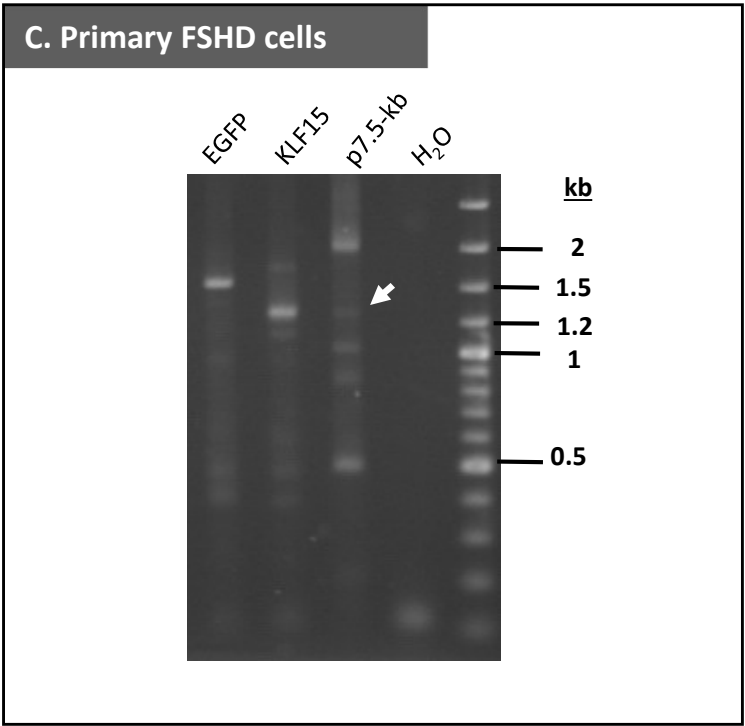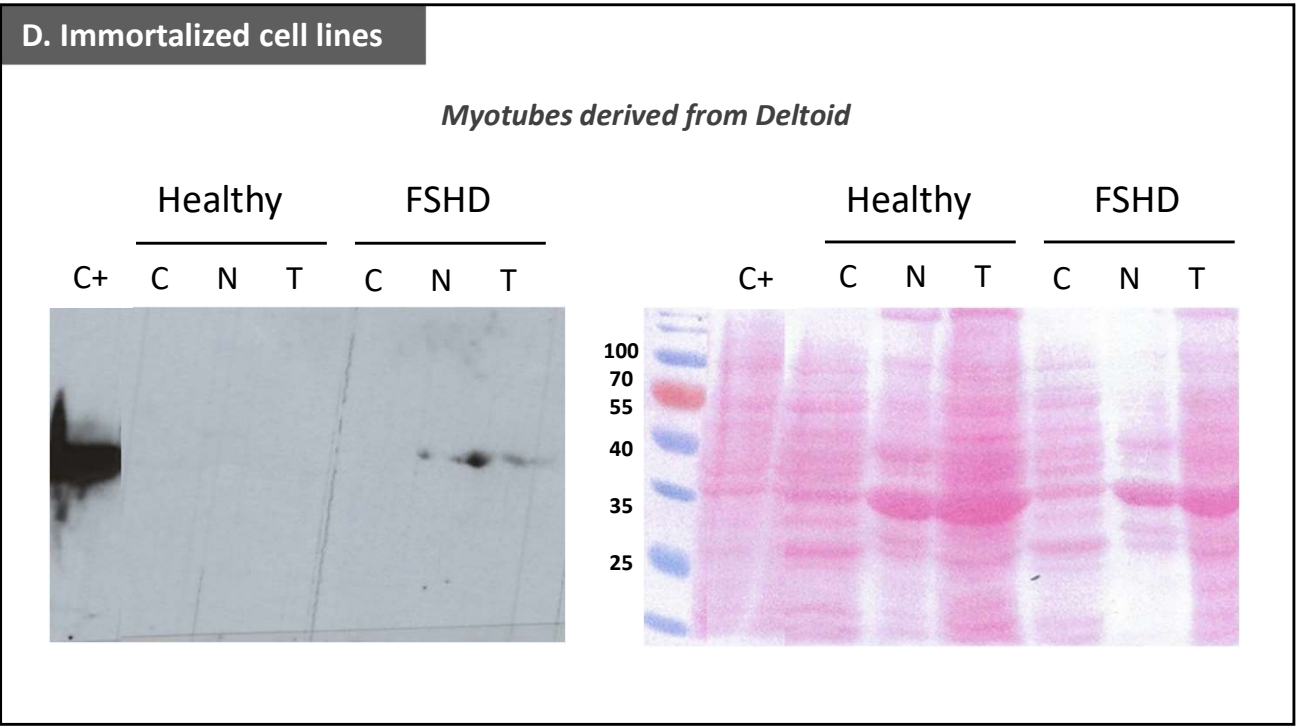

Supplement: Supplementary file 2 — Additional file 2: Figure S1. DUX4C mRNAs and protein in muscle cells. (A-C) Total RNAs were extracted, retro-transcribed and analyzed by 3’RACE with a DUX4C-specific primer as described in Methods. Samples of such 3’RACE products (see Table S2 for sequences) from RNAs of healthy or FSHD immortalized (A) or primary muscle cells (B) were analyzed by electrophoresis on agarose gels. P: proliferating myoblasts, C:confluent myoblasts, D: differentiating myoblasts (incubated either 1, 3, 7 or 9 days in adifferentiation medium). (C) DUX4C 3’RACE products of RNAs from primary muscle cells transfected with either an EGFP- or KLF15-expression vector. In parallel, DUX4C 3’RACE products of RNAs from C2C12 cells transfected with the 7.5 kb human genomic fragment comprising DUX4C (p7.5-kb) as described in (2). The arrows indicate the 1.2-kb product (intron 2 spliced out). Negative control: H2O in place of cDNA during the nested PCR. (D) Total (T), nuclear (N) and cytoplasmic (C) protein extracts of healthy or FSHD immortalized muscle cells were separated by SDS-PAGE transferred to a nitrocellulose membrane. The membrane was blocked and then incubated with rabbit anti-DUX4c serum followed by secondary antibodies coupled to HRP and revealed with the Super Signal West Femto maximum sensitivity substrate (see Material and Methods). C+ is the positive control i.e. an extract of cells transfected with pCIneo-DUX4C as described in (2). [file 13395_2022_310_MOESM2_ESM.pdf]

Fig. S2

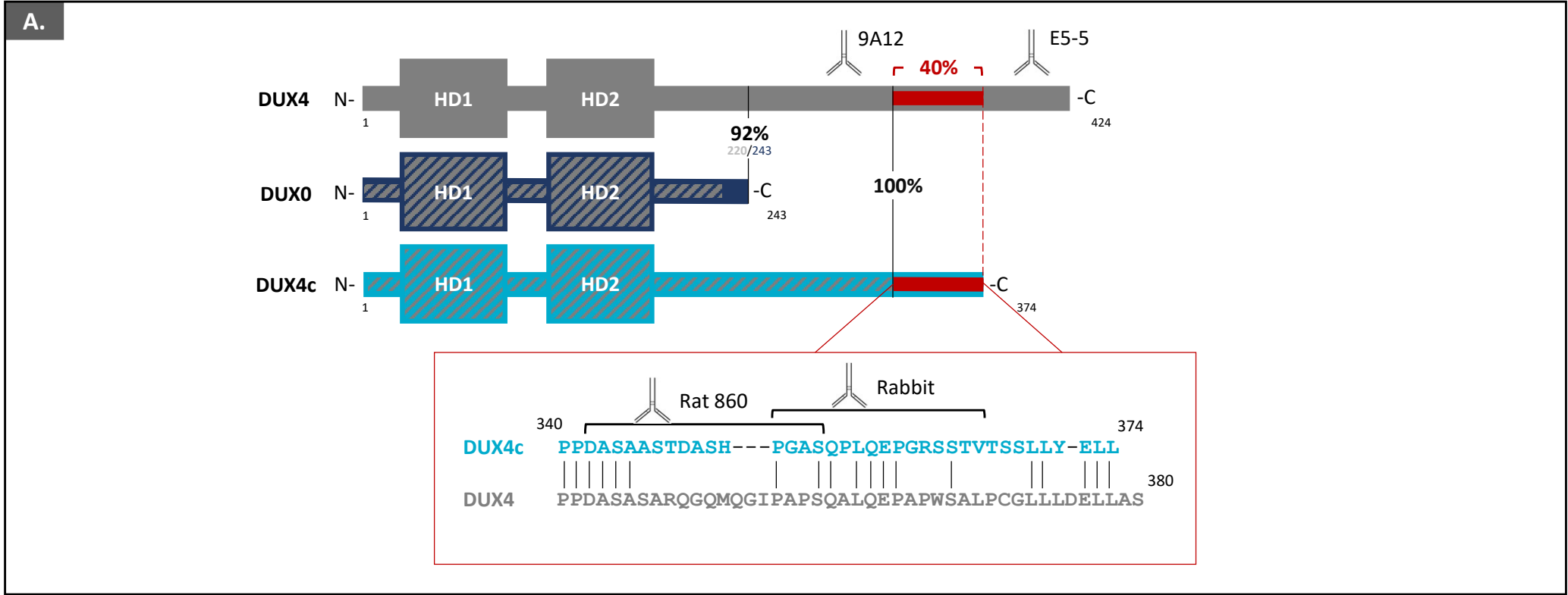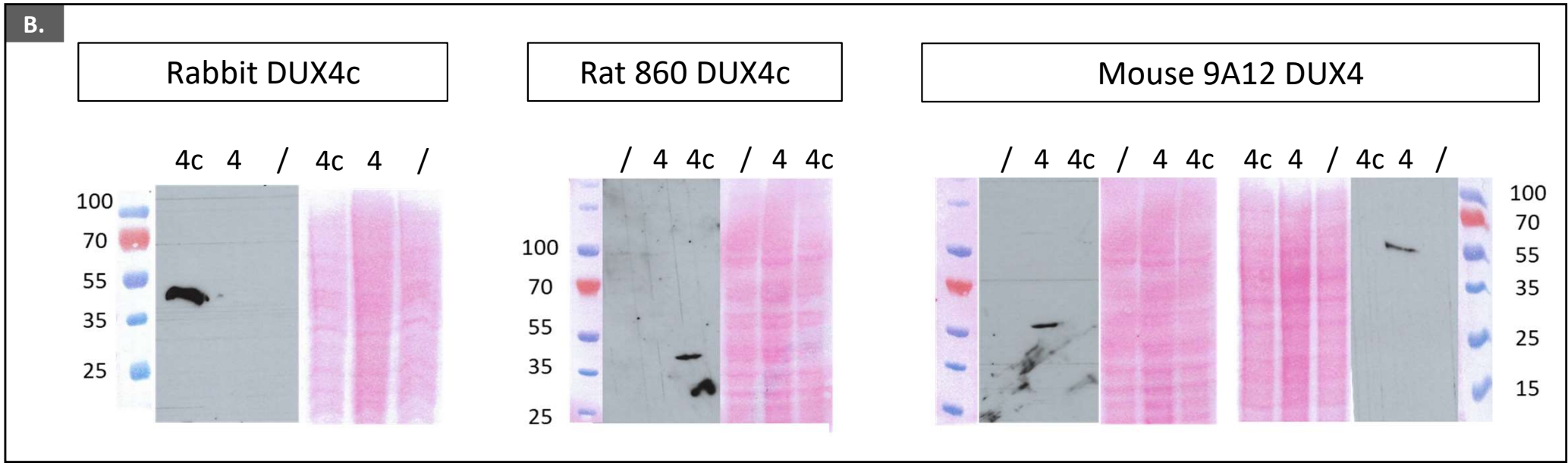

Supplement: Supplementary file 3 — Additional file 3: Figure S2. Validation of the rat anti-DUX4c serum. (A) Schematic alignment of DUX4, DUXO and DUX4c protein sequences. The percent identities indicated on vertical black lines correspond to the sequences aligned from the N terminus to this point. The percent value in red refers to the short sequence alignment in red. The specific DUX4c peptide sequences used for rabbit (2) or rat immunization are shown, as well as the regions targeted by the mouse 9A12 and the rabbit E5-5 monoclonal antibodies.raised against DUX4. (B) HEK293 cells were transfected with either pCIneo, pCIneo-DUX4 or pCIneo-DUX4c. Twenty-four hours later, cells were harvested and total proteins were extracted, separated by SDS-PAGE and transferred to a nitrocellulose membrane for immunodetection with the mentioned primary antibodies as described in Fig. S1. [file 13395_2022_310_MOESM3_ESM.pdf]

Fig. S3

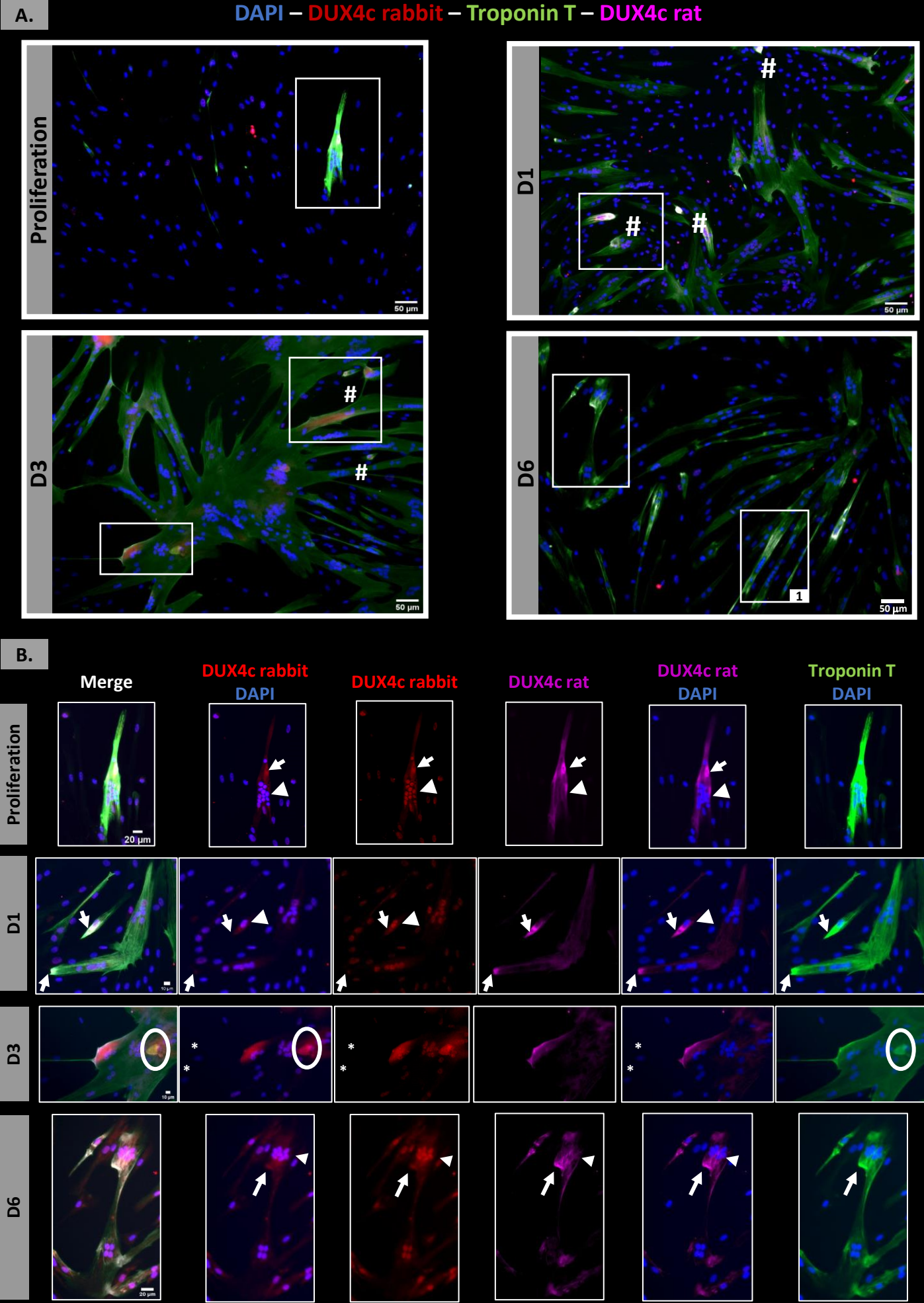

Supplement: Supplementary file 4 — Additional file 4: Figure S3. Time-course of DUX4c expression in primary FSHD muscle cells. (A) Microscope images of enlarged fields presented in Figs. 2 or S3B (boxed) showing merged immunofluorescence of DUX4c with both rabbit (red) and rat (pink) antisera, Troponin T (TnT-green) and DAPI (blue). The yellow signal corresponds to intense TnT staining co-localized with DUX4c detection. Myotubes with a cluster of 3 to 10 nuclei and high DUX4c nuclear and cytoplasmic labeling are indicated (#): they appear like ‘comets’ and were scarcely observed. For the Serratus Posterior Superior (SPS) muscle cultures, the following numbers of microscopic fields were analyzed at the indicated times, 5 fields in proliferation (P), at days D1 and D3; and 4 fields at day D6. For the Serratus Posterior Inferior (SPI) muscle culture, 3 fields were analyzed in P and at day D6); 11 fields at D1 and 5 fields at D3. (B) Additional magnified regions presenting DUX4c staining as described in Fig. 2. Arrows point to DUX4c cytoplasmic labeling, and stars to DUX4c-negative nuclei. The strongest DUX4c nuclear staining was detected in TnT-expressing cells (arrowheads). The circle highlights cytoplasmic TnT accumulation that co-localized with DUX4c when it was detected by immunostaining with the rabbit but not the rat antiserum. The opposite was observed in Fig. 2 (at D1): TnT co-localized with DUX4c immunostaining observed with the rat but not the rabbit antiserum. [file 13395_2022_310_MOESM4_ESM.pdf]

Fig. S5

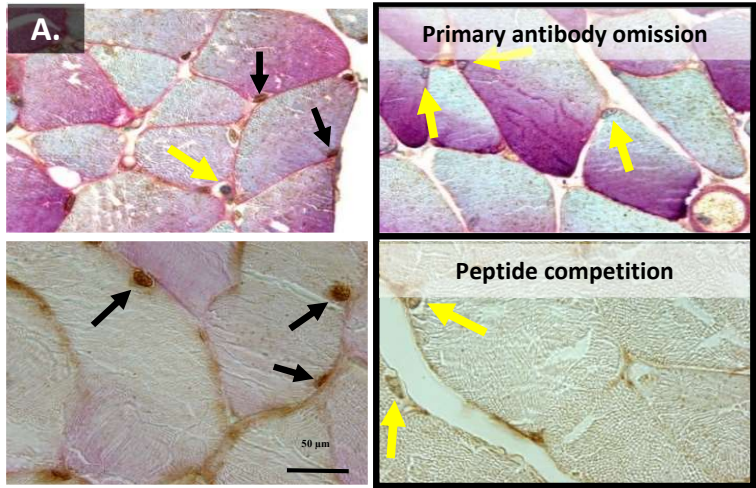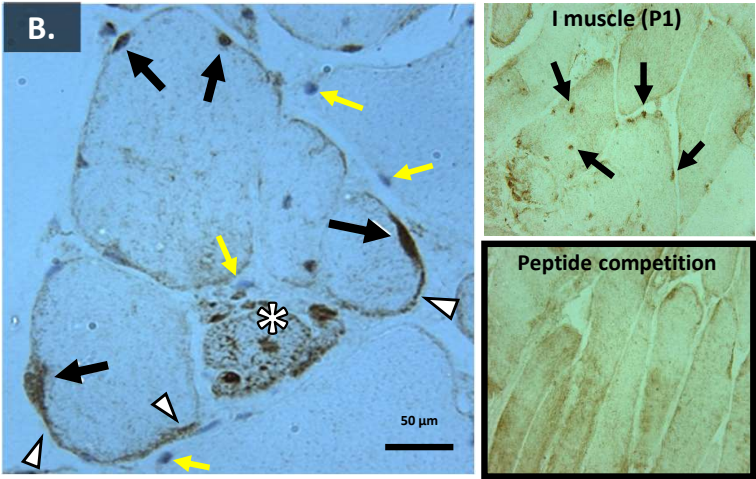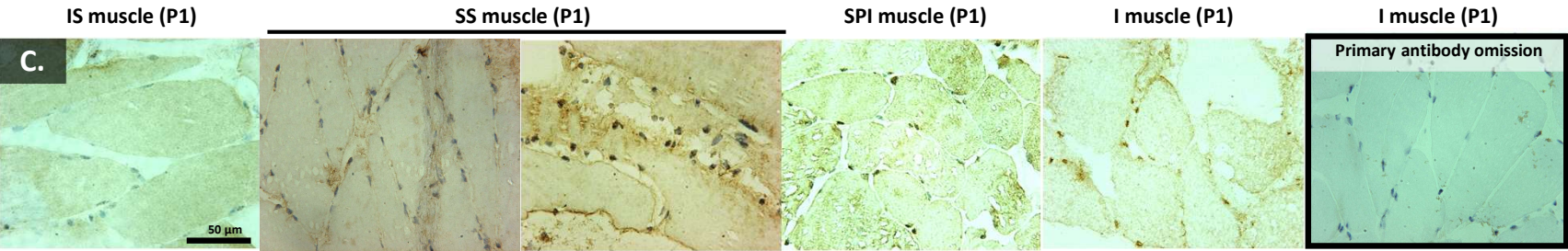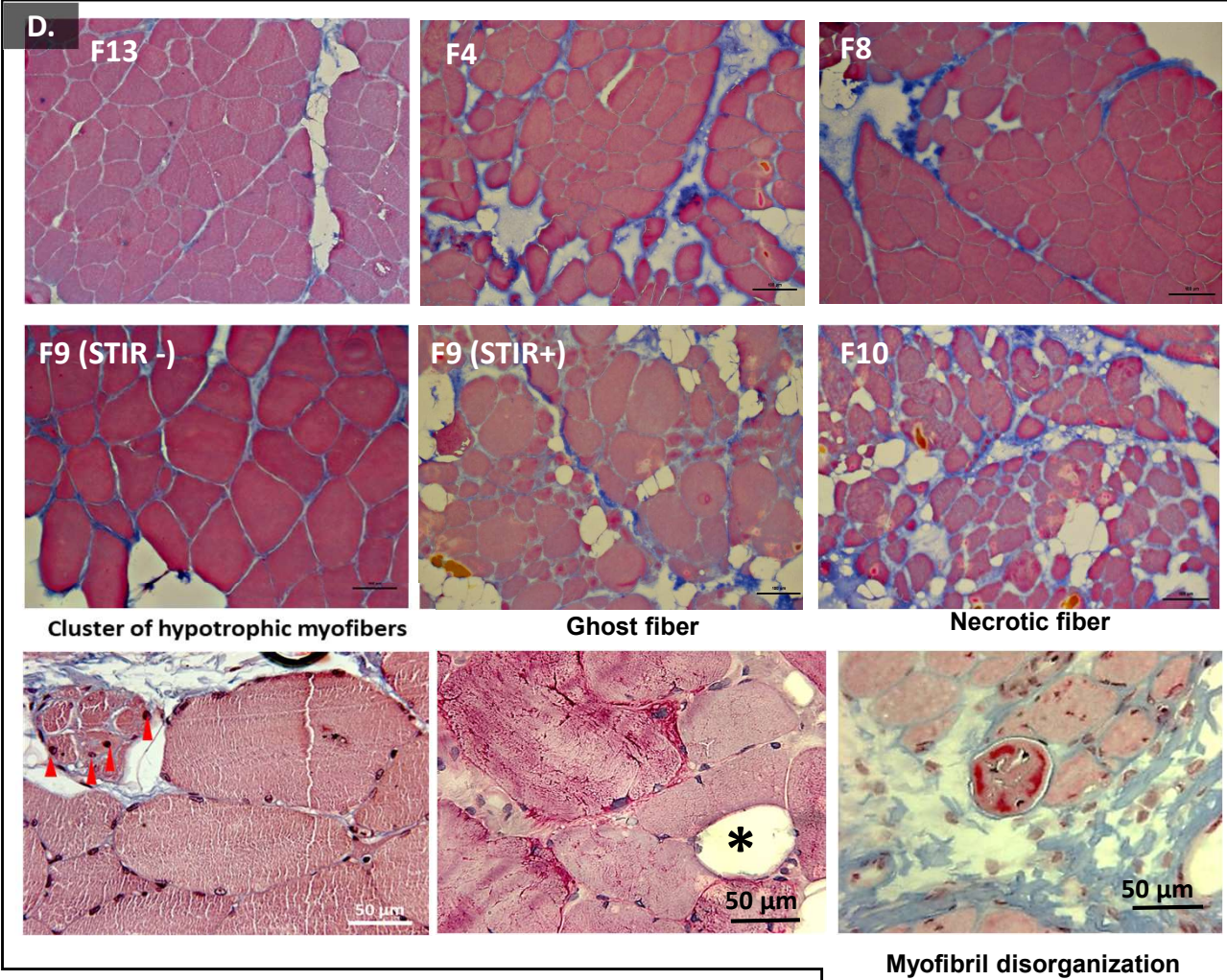

Supplement: Supplementary file 6 — Additional file 6: Figure S5. DUX4c detection by immunohistochemistry in muscle sections and histological alterations in FSHD muscles. (A) Immunostaining was performed with the rabbit serum raised against a DUX4c peptide (2) and secondary antibodies coupled to HRP on healthy muscle sections using the TSA amplification system with DAB detection. PAS counterstaining (pink) delimits muscle fibers, including satellite cells. Pictures were only taken in areas presenting DUX4c-positive nuclei (black arrows). Yellow arrows point to DUX4c-negative nuclei. (B-C) DUX4c immunostaining was performed on FSHD muscle sections as in (A) except a standard procedure was used with DAB detection and hemalun counterstaining (negative nuclei in blue, yellow arrows). The black arrows indicate strong DUX4c labeling in myonuclei at the periphery of fibers adjacent to an angular fiber (star, left panel) or presenting delocalized nuclei (right panel). Yellow arrows highlight DUX4c-negative nuclei. DUX4c staining could also present a granular aspect in the sarcoplasm (star) or extend from a peripheral nucleus to just under the basement or the sarcoplasmic membrane (arrowheads). (C) Sections of FSHD muscles from a single patient were treated (as in Fig. 2C) in parallel to detect DUX4c: either no (infraspinatus, IS, muscle) or variable DUX4c immunostaining from scarce (sub-scapularis, SS, muscle: in some nuclei at the periphery of a degenerating fiber) to several positive peripheral nuclei (serratus posterior inferior, SPI, and intercostalis, I, muscles). (A-C) Negative controls (boxed panel) correspond to either omission of the primary antibody or antigenic peptide competition on consecutive sections. (D) Muscle sections derived from patients presenting a CSS <5 (upper panels) or > 5 (middle and bottom panels) (characterized in Table S4) were stained with Heidenhain blue trichrome. The bottom panels present a cluster of hypotrophic myofibers with peripheral or delocalized nuclei (red arrowheads [file 13395_2022_310_MOESM6_ESM.pdf]

Fig. S6

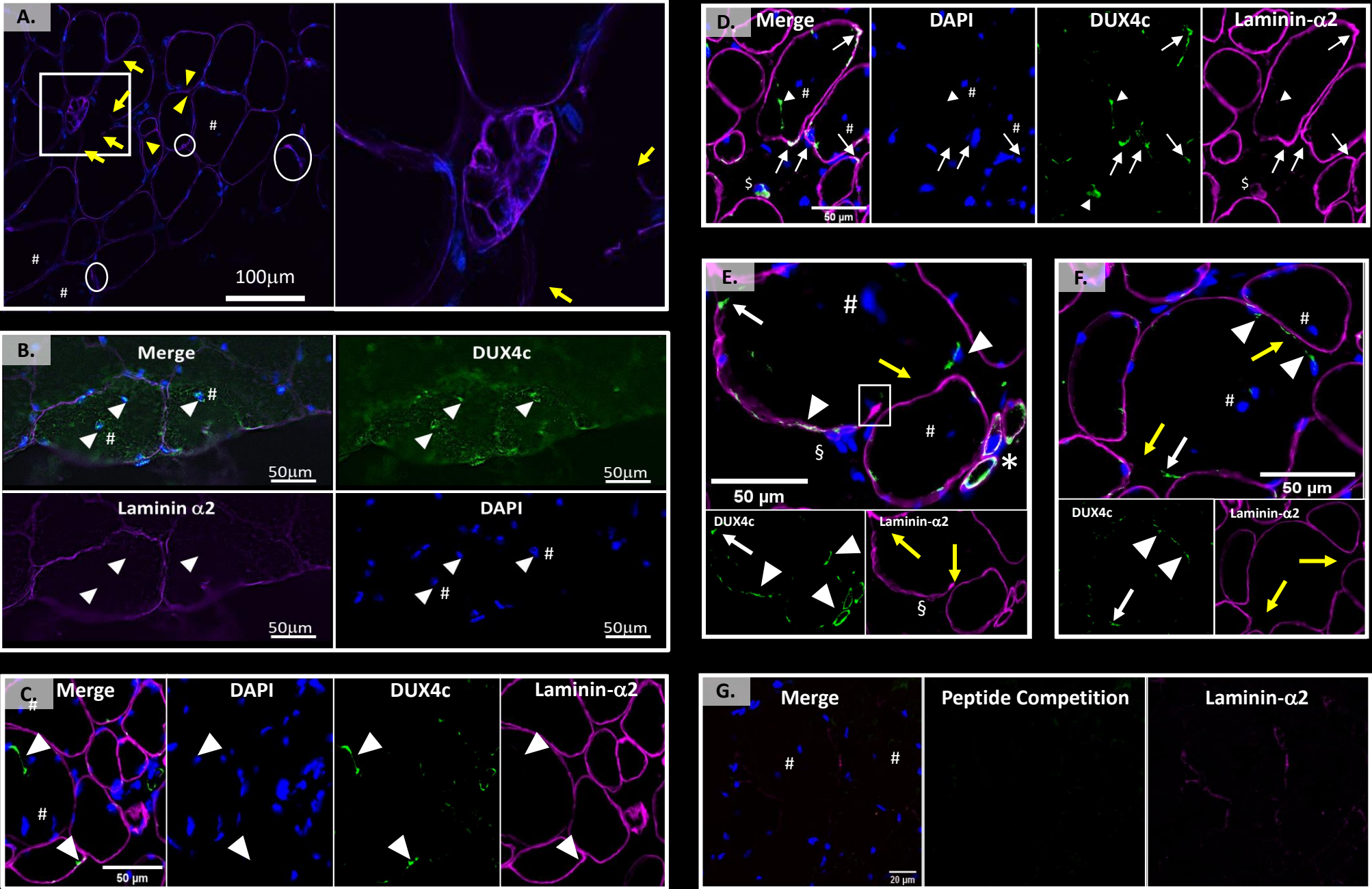

Supplement: Supplementary file 7 — Additional file 7: Figure S6. DUX4c and laminin-α2 detection by co-immunofluorescence in FSHD muscle sections. Immunofluorescence was performed on FSHD muscle sections as in Fig. 3 using the primary rabbit anti-DUX4c and rat anti-laminin-a2 sera followed by appropriate secondary antibodies coupled to different Alexa Fluor molecules. Images were taken by an epifluorescence microscope. To better visualize DUX4c-specific staining, a higher image contrast was applied for panels C-F. (A) A rare cluster of small muscle cells (boxed, magnified to the right) next to myofibers presenting lamina defects such as either a large loss of the expected staining (yellow arrows), or a punctuated disruption, or a very thin staining (yellow arrowheads). Adjacent fibers present delocalized nuclei (#) and circles highlight areas with either intense laminin staining or a double lamina. (B) DUX4c detection in or next to delocalized nuclei (#) and in the sarcoplasm (granular aspect) in two adjacent angular fibers, similar to the one shown in Fig. S5B (star) and in our previous published data in a muscle section from another patient (Figure 9 in Ansseau et al 2016). Of note, the image was taken in a region difficult to focus on. (C-F) Pictures taken on the same muscle section. In a few myofibers, DUX4c staining is detected as a ‘line’ in the sarcoplasm (arrowheads) of fibers presenting delocalized nuclei (#) and this DUX4c signal can be either between delocalized nuclei (C) or in the sarcoplasm with a faint laminin-α2 staining in the immediate vicinity (D). (D) Adjacent fibers also present delocalized nuclei (#). Arrows point to DUX4c-positive regions of these fibers or of a nearby hypotrophic fiber that present an unusual round or angular shape, sometimes around a nucleus and with a higher DUX4c staining just under the lamina. A very small fiber with a faint laminin-α2 staining ($) also presents intense DUX4c sarcoplasmic staining (arrowhead). (E) At proximity, very small myofibers (< 15 µm [file 13395_2022_310_MOESM7_ESM.pdf]

Fig. S7

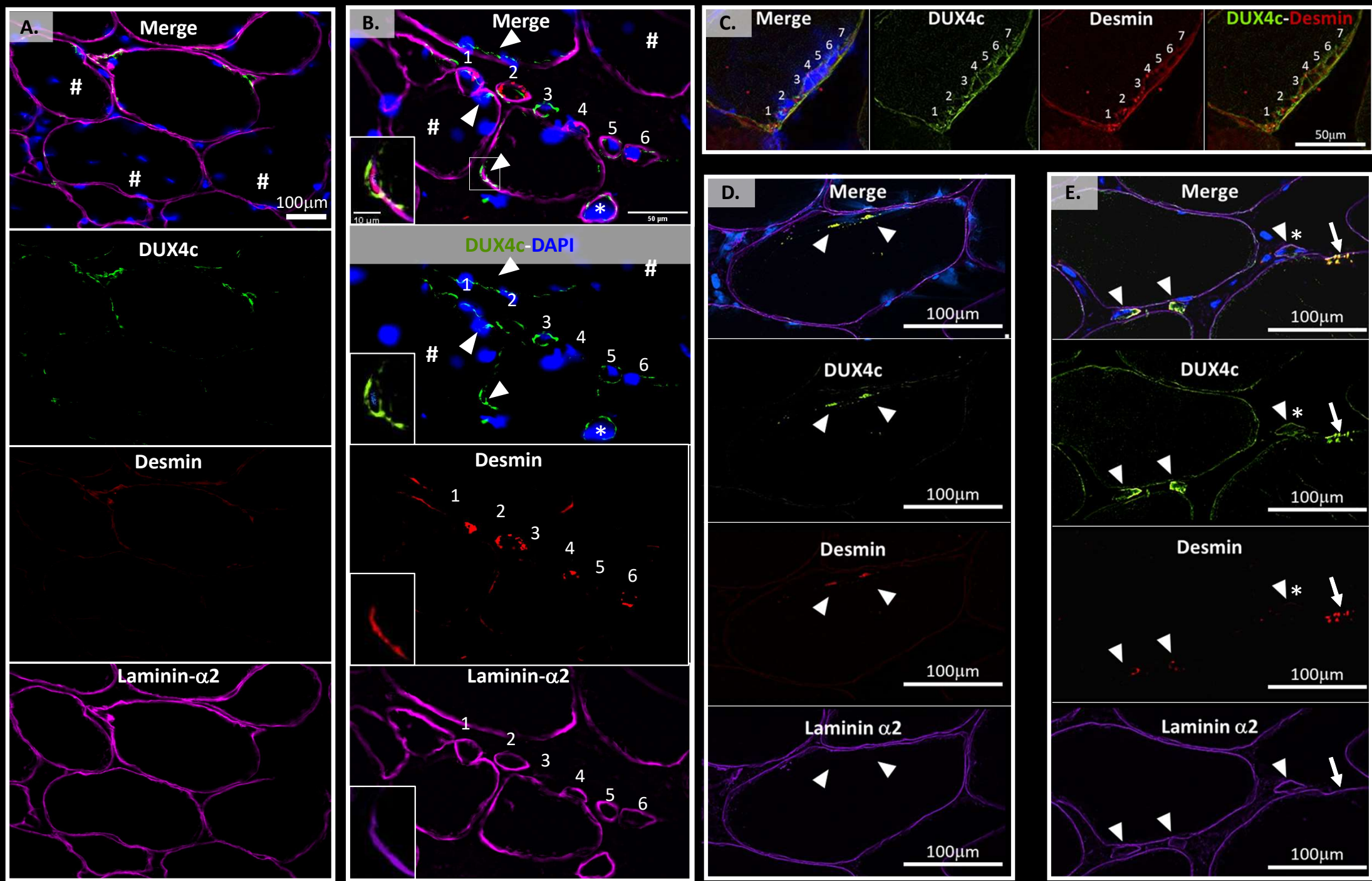

Supplement: Supplementary file 8 — Additional file 8: Figure S7. DUX4c-desmin co-detection by immunofluorescence in FSHD muscles. Immunofluorescence was performed on FSHD muscle sections as in Fig. 3. A background staining around all myofibers is observed with the rabbit anti-DUX4c serum (panels C-E). To better visualize DUX4c-specific staining, a higher image contrast was applied for panels A-B. (A) Enlarged picture of the area presented in Fig. 3C showing that the myofiber with the unusual triangular tip presenting DUX4c and desmin staining is surrounded by fibers with delocalized nuclei (#). (B) DUX4c was detected in aligned round hypotrophic fibers (numbered from 1 to 6) either in the sarcoplasm, sometimes as a ‘line’, (myofibers 1, 2, 4 and 6 that also present intense desmin staining) or at the myocyte periphery (myofibers 3 and 5) as also shown in a larger hypotrophic fiber (star). Adjacent fibers present DUX4c staining in some region of their periphery, and a more intense one around a nucleus (enlarged in the inset box). Of note, the image was taken in a region difficult to focus on; the adjacent section presenting the same hypotrophic fibers was used for DUX4 detection (Fig S11A) and confirmed laminin-a2 staining around myofiber 3. (C) Intense DUX4c (green) and intense desmin (red) co-staining around aligned nuclei (numbered from 1 to 7) at the periphery of a myofiber (surrounded by laminin-α2 staining, purple). (D-E) Partial DUX4c and desmin co-localization in putative regenerating muscle cells (arrowheads). In panel E, DUX4c presents the same staining ‘polarity’ (at one side of the hypotrophic fiber) than the intense desmin staining. Another hypotrophic fiber (star) with almost no desmin detection presents an a specific DUX4c staining at its periphery, near an adjacent normal size fiber with DUX4c-desmin co-detection at its periphery (arrow). [file 13395_2022_310_MOESM8_ESM.pdf]

Fig. S8

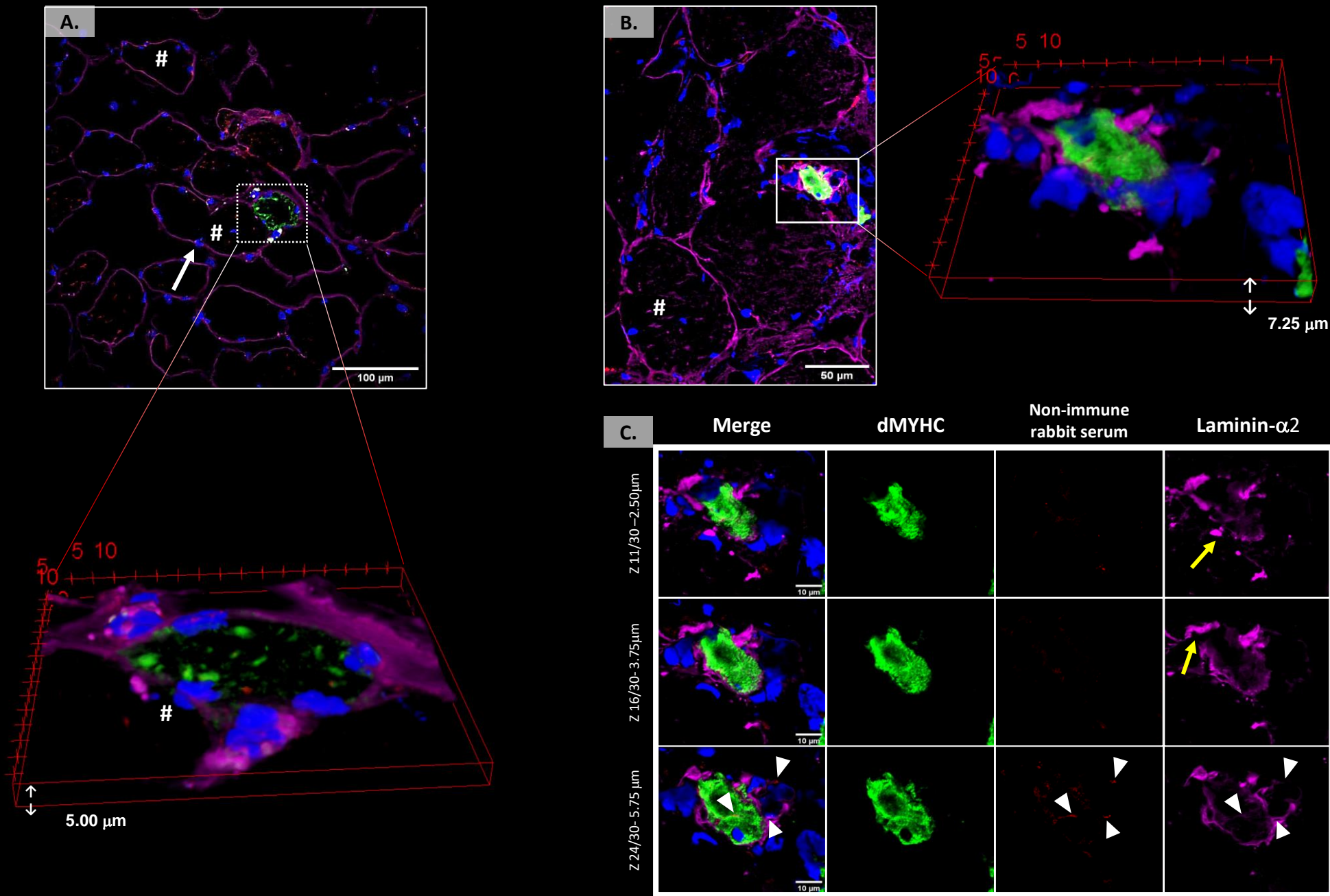

Supplement: Supplementary file 9 — Additional file 9: Figure S8. DUX4c is immunodetected in regenerating myofibers. (A) Enlarged region of Fig. 4B with a 3D image reconstruction at the confocal microscope showing that the regenerating normal size myofiber (dMyHC-positive, green) seems to fuse with the adjacent fiber presenting a central nucleus (#, in top panel). (Bottom panel) 3D image reconstruction with an enhanced pink fluorescence detection shows a faint discontinuous laminin-a2 staining (suggesting a fusion event) next to a nucleus (pointed with #) that is thus not delocalized/central. The ‘ajdacent’ fiber with a central nucleus appears to extend at its other side to another fiber (see top panel) surrounded with a partial laminin-a2 staining (arrow). (B-C) The negative control is an adjacent section used in parallel with a non-immune rabbit serum instead of anti-DUX4c serum. Arrowheads point to the most intense negative staining found outside fibers or between regenerating hypotrophic fibers (corresponding to a lamina part). In addition, some intense stained laminin-a2 areas highlighted by the yellow arrows do not present any labeling with the non-immune rabbit serum. [file 13395_2022_310_MOESM9_ESM.pdf]

Fig. S9

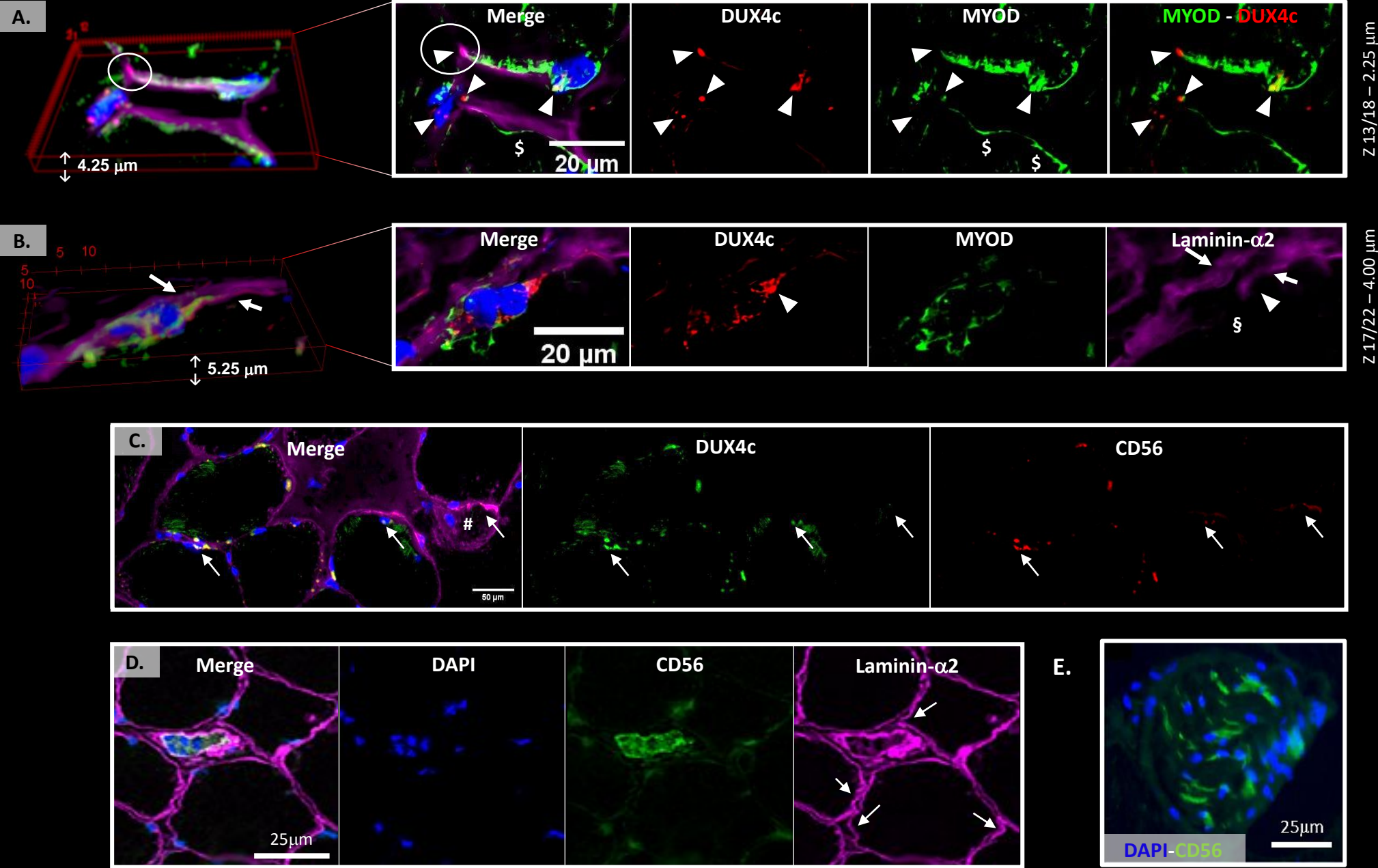

Supplement: Supplementary file 10 — Additional file 10: Figure S9. DUX4c co-detection with MYOD or CD56 as myogenic cell markers, and CD56 immunodetection pattern in FSHD muscle sections. Immunofluorescence was performed on FSHD muscle sections as in Fig. 5. (A-B) 3D reconstruction with a Z section showing merged and individual detections of DUX4c (red), MYOD (green) and laminin-a2 (purple). (A) Magnification of box 3 from Fig. 5. MYOD is immunodetected in some nuclear regions and in an area partially surrounding a peripheral nucleus where its signal extends from just under the lamina to the end of an intense laminin-a2 staining (circle). An adjacent fiber also presents a MYOD staining extending to part of its periphery (§). Several intense DUX4c signals were observed (arrowheads): next to or inside the nucleus in co-detection with MYOD; co-localized with an intense laminin-a2 staining (circle); and in another cell (left), next to a MYOD-positive nucleus either inside a nearby nucleus or next to a sarcoplasmic MYOD staining. (B) DUX4c detection around and inside two apparently bound nuclei at the fiber periphery: both nuclei are surrounded by MYOD staining but without DUX4c co-localization. The larger DUX4c positive area on one side (arrowhead) is surrounded by laminin-a2 staining that does not fully extend around the nuclei (§) suggesting these cells may be in the process of fusion. (C) Co-immunodetection of DUX4c (red) and CD56 (green) as a satellite/myogenic cell marker with respective specific monoclonal antibodies. DUX4c and CD56 co-localized at the periphery of adjacent myofibers (arrowheads). A myofiber with unusual shape and a delocalized nucleus (#) is observed nearby. The arrow points to a CD56 staining not associated with DUX4c labeling. (D-E) The CD56-positive cells are sometimes observed either (D) in small clusters between fibers or (E) in larger heterogenous cell clusters, where only some nuclei are next to CD56 labeling. Arrows point to abnormal tips in adjacent myofibers with an unusu [file 13395_2022_310_MOESM10_ESM.pdf]

Fig. S10

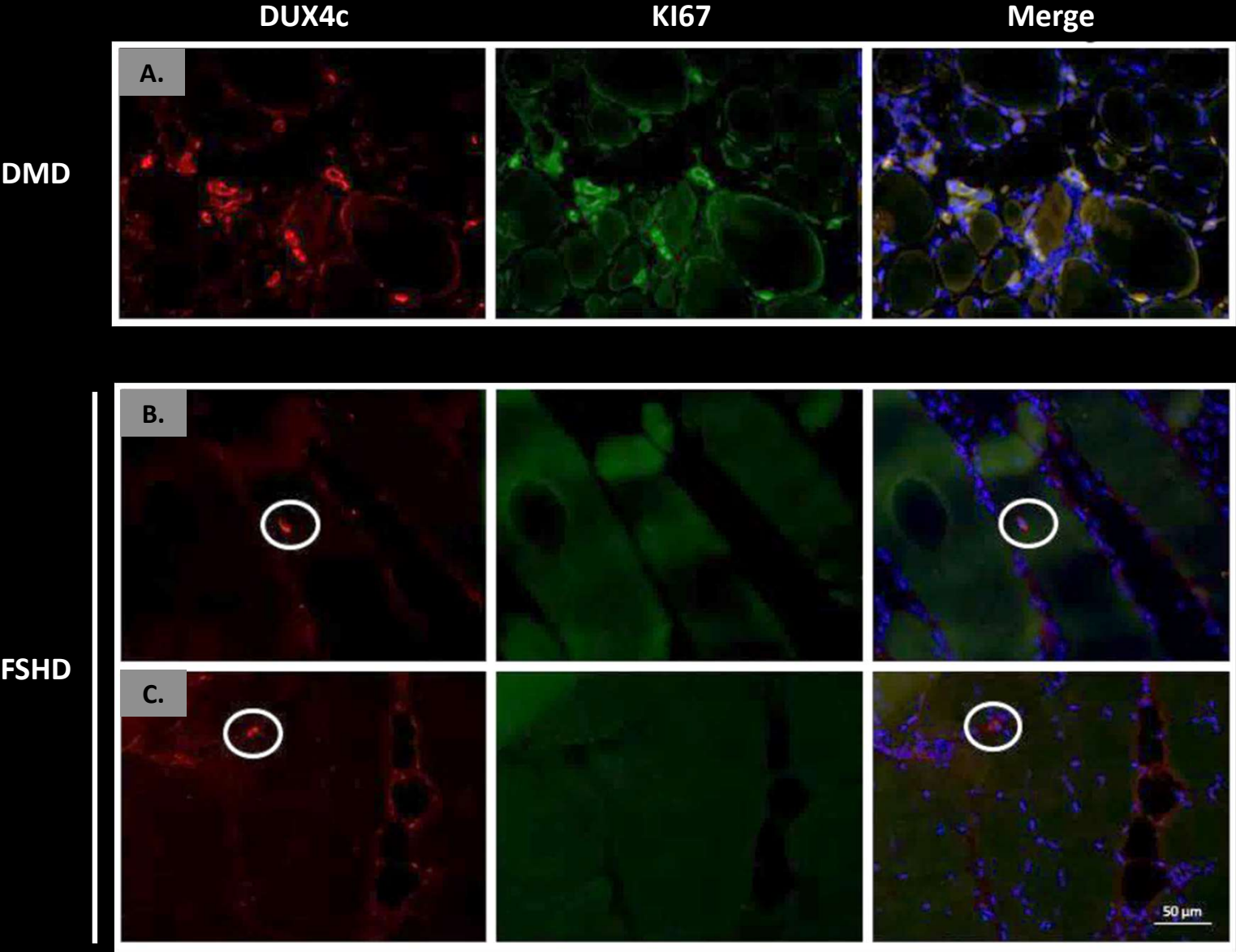

Supplement: Supplementary file 11 — Additional file 11: Figure S10. DUX4c co-detection with Ki67 proliferation marker in DMD and FSHD muscles. Immunofluorescence was performed on DMD or FSHD muscle sections as in Fig. 5 with a monoclonal antibody against Ki67 instead of MYOD. (A) DUX4c-Ki67 co-labeling in DMD muscles. (B-C) No Ki67 staining is observed in the 7 FSHD muscle sections (Table S4). Rare DUX4c positive signals in putatively delocalized nuclei (longitudinal section, B) or near nuclei (transversal section, C). [file 13395_2022_310_MOESM11_ESM.pdf]

Fig. S11

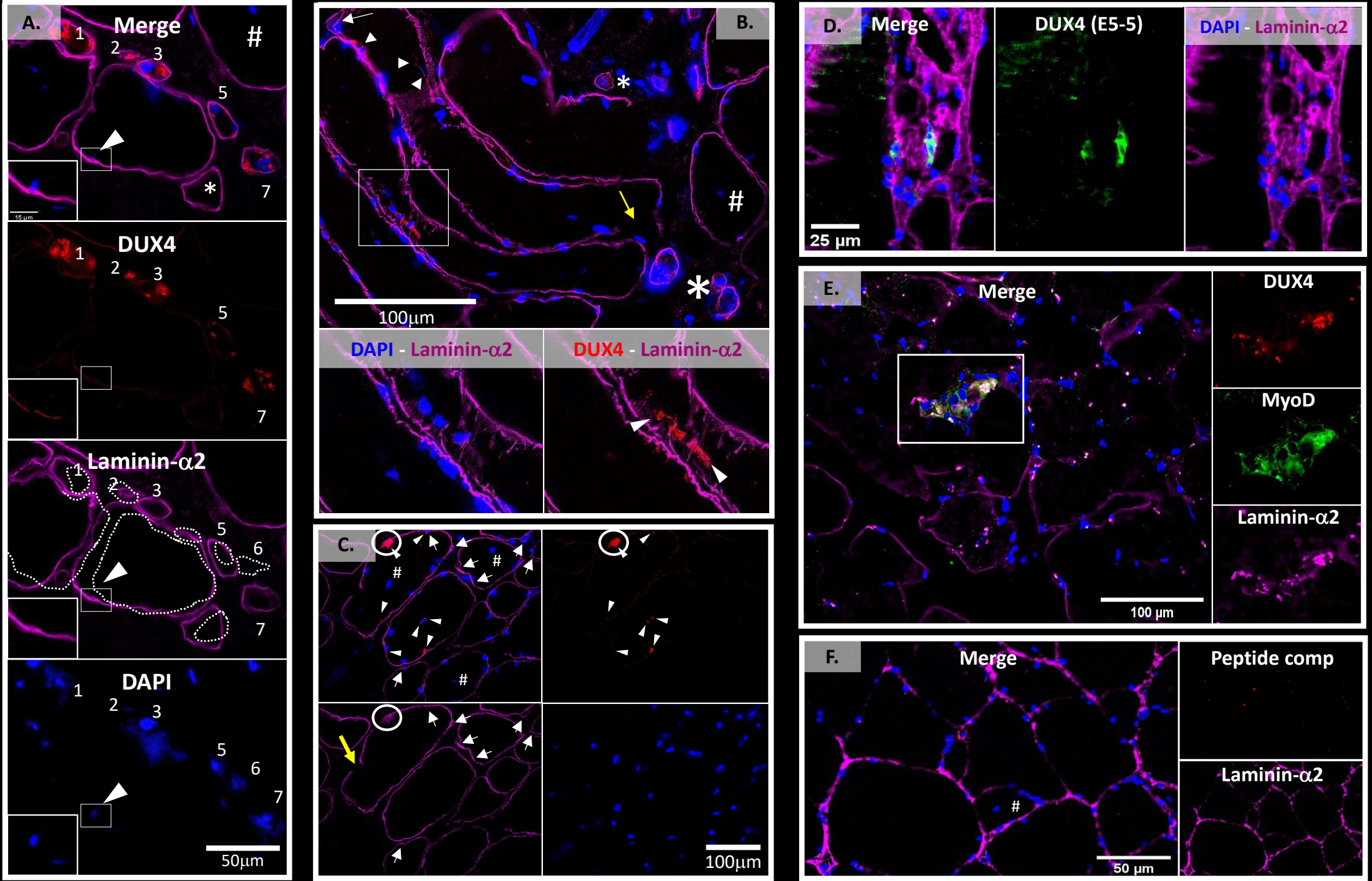

Supplement: Supplementary file 12 — Additional file 12: Figure S11. DUX4 detection in regenerating myofibers of FSHD muscles. (A-C) Immunofluorescence was performed on FSHD muscle sections as in Fig S6 with the use of 9A12 (A-C) or E5-5 (D-E) monoclonal antibodies instead of anti-DUX4c serum. (A) Section adjacent to the one used in Fig. S7B showing desmin-positive aligned and round hypotrophic fibers, near a fiber with a central nucleus (#). 9A12 staining in dots (red) was detected around the nuclei or in the sarcoplasm of these fibers (numbered 1 to 7). Fiber 3 is surrounded by a laminin-a2 staining in contrast to Fig. S7B which only presents a peripheral DUX4c staining. Fiber 6 is missing in this section. The layer of myofibers of the section shown in Fig. S7B is shown by the dotted lines. The staining with 9A12 was distinct from the one observed with antisera against DUX4c in the same fibers, f.i., not in line or at the fiber periphery. Moreover, the adjacent myofibers (including the larger hypotrophic fiber pointed by *) are negative with 9A12 in contrast to the staining observed with anti-DUX4c serum. The arrowhead points to a myofiber with an intense DUX4c staining area around a nucleus in Fig. S7B. A nucleus is at a similar position in the present muscle section (inset). (B) DUX4 immunostaining is detected around 3 aligned nuclei localized in the longitudinal axis of a myofiber showing a shrunk region (boxed, magnified in the bottom panels) with faint laminin-α2 staining. Nearby myofibers present delocalized nuclei or an hypotrophic morphology. A yellow arrow points to another laminin defect in the same fiber. A weak DUX4 staining is also observed next to nuclei (arrowheads) or in hypotrophic fibers (stars): the bottom star is in a magnification of the region with DUX4 detection presented in Fig. 6A. The arrow points to a double lamina close to a peripheral nucleus. (C) Immunodetection with 9A12 mAb in normal size fibers with a central nucleus next to fibers with delocalized nuclei (#). Stainin [file 13395_2022_310_MOESM12_ESM.pdf]

Fig. S12

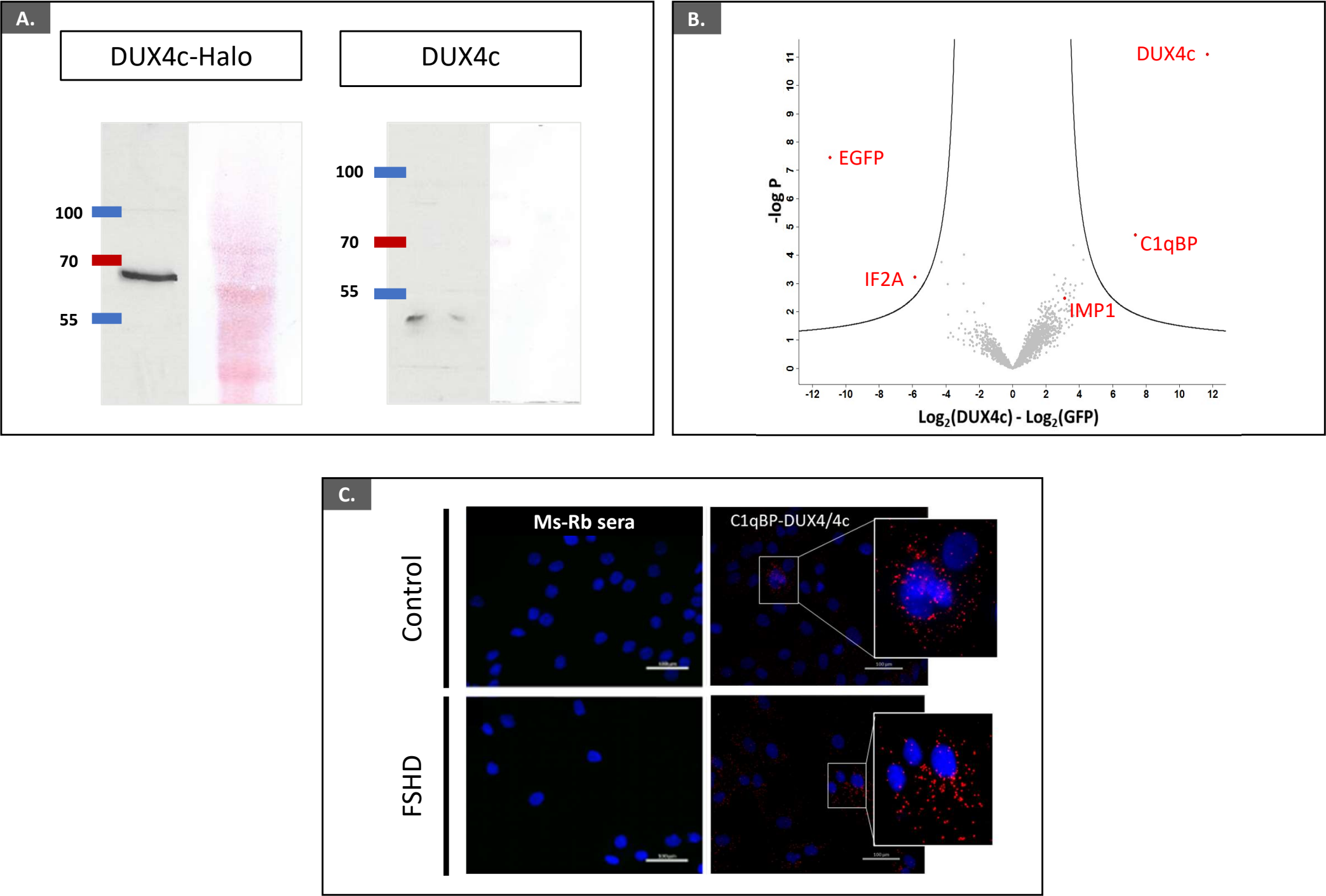

Supplement: Supplementary file 13 — Additional file 13: Figure S12. C1qBP is the major DUX4c protein partner. (A) Purification of HaloTag-DUX4c protein complexes. HEK293 cells were transfected with pHaloTag-DUX4c expression vector. Cells were harvested 24 h later and lysed. The HaloTag protein complexes were then purified by affinity chromatography on Halo-Link resin and DUX4c protein complexes released by digestion with TEV protease to remove the Halo-Tag. Twenty-five μg proteins of the protein lysate (before chromatography, left) and the purified Halo-Tag complex (after TEV cleavage, right) were separated by SDS-PAGE followed by a Ponceau staining. After washing, DUX4c was immunodetected at the expected size either fused with the Halo-Tag (left) or after tag removal (right). (B) Volcano plot comparing the abundances of proteins co-purified with fusion proteins of DUX4c-or EGFP to Halo-Tag. The black curves mark the boundaries for a false detection rate of 1%. X-axis: the Log2 difference of abundances between the two conditions. Y-axis: P-value estimate for each protein. Proteins of interest are indicated in red. N=6. (C) C1QBP and DUX4/4c co-localization was detected by in situ Proximity Ligation Assay performed on healthy or FSHD immortalized myoblasts following fixation with PAF, using a mouse anti-C1QBP and a rabbit anti-DUX4/4c serum and appropriate secondary antibodies for PLA signal amplification (see Material and methods). Red dots correspond to these protein co-localizations. Negative controls used in parallel with non-immune mouse (Ms) and rabbit (Rb) sera in place of one or both primary antisera as indicated. N=3 biological replicates. [file 13395_2022_310_MOESM13_ESM.pdf]

Fig. S13

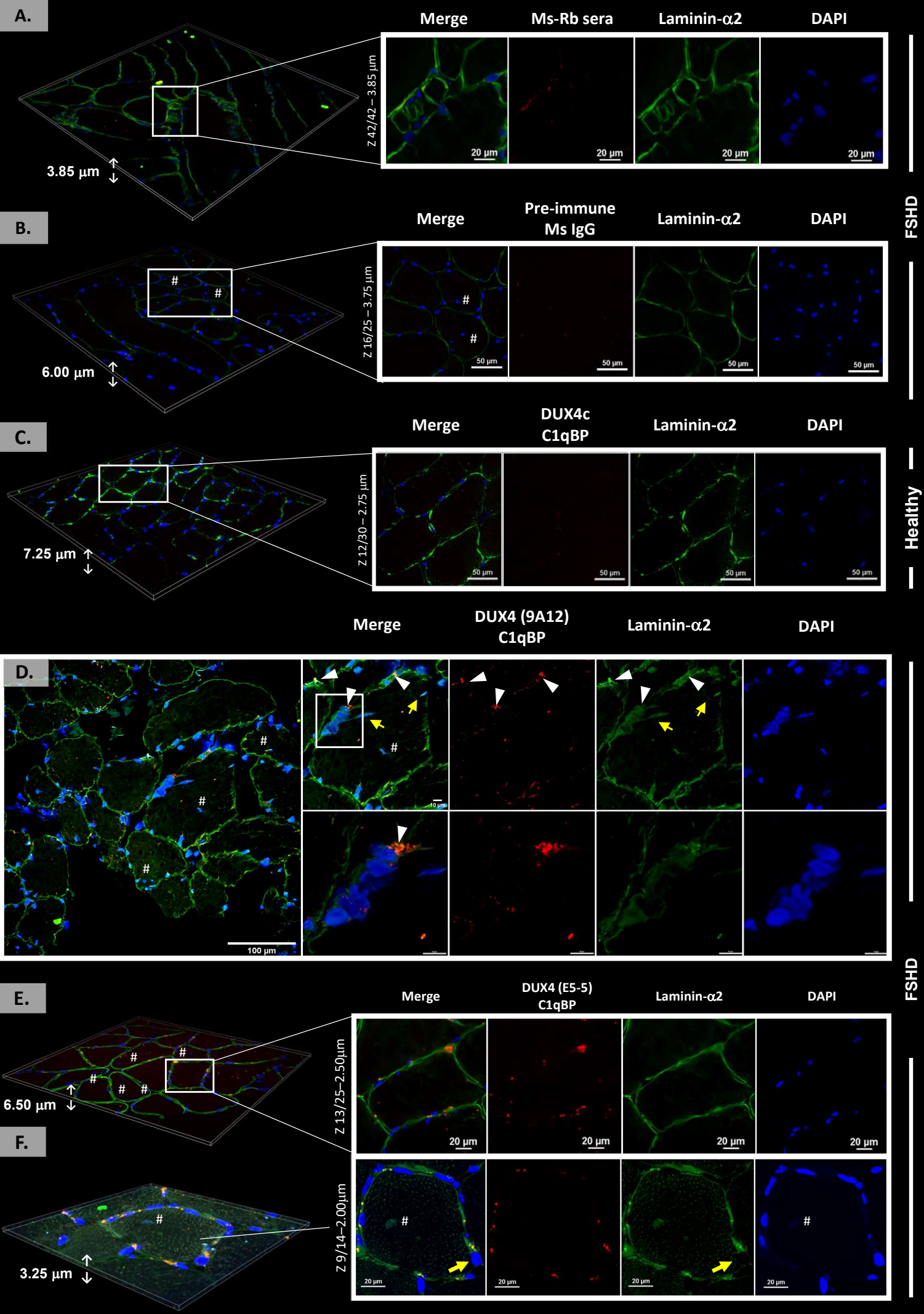

Supplement: Supplementary file 14 — Additional file 14: Figure S13. PLA experiments in myofibers (negative controls and detection of DUX4-C1qBP interactions). (A-C) Negative PLA controls for interactions between C1qBP and DUX4c. 3D reconstruction and examples of a Z-axis image for each negative control done in parallel with the PLA procedure of Fig. 7. In place of the specific primary antiserum pair used in Fig. 7, we incubated adjacent FSHD muscle sections with either mouse (Ms) and rabbit (Rb) non-immune sera (A) or the rabbit pre-immune serum with mouse IgGs (B). We also used healthy muscle sections with the pair of rabbit anti-DUX4c and mouse anti-C1qBP sera (C). No PLA dots in large cluster were found in these sections, only a specific staining was observed in the lamina. Pictures were taken with the same parameters for all the PLA reactions in areas with either hypotrophic fibers (A) or fibers with delocalized nuclei (B). PLA was performed with the non-immune serum pair on muscle sections from 3 patients with FSHD (and 1 healthy control, not shown), and the pre-immune serum combined with Ms IgGs on the FSHD muscle section. Another FSHD muscle section was used for PLA with the primary antiserum pair after competition with the DUX4c immunogenic peptide (pictures similar to A-B were observed, not shown). The pair of anti-DUX4c and anti-C1qBP primary antisera was used on 3 healthy muscle sections. (D-F) DUX4 interacts with C1qBP in FSHD myofibers. (D) Same procedure as above except that the primary antibodies used for PLA are the mouse 9A12 mAb and a rabbit anti-C1qBP serum. The only PLA dots we take into account are the ones in clusters (arrowheads). Such a cluster was found in a myofiber presenting delocalized nuclei, next to very closely aligned nuclei at the fiber periphery. They all seem surrounded by a lamina. Arrows point to an incomplete laminin-a2 staining region inside this ‘fiber’ that coincide with flat nuclei suggesting they are peripheral ones and that this ‘fiber’ is in fact in a fusi [file 13395_2022_310_MOESM14_ESM.pdf]

Fig. S14

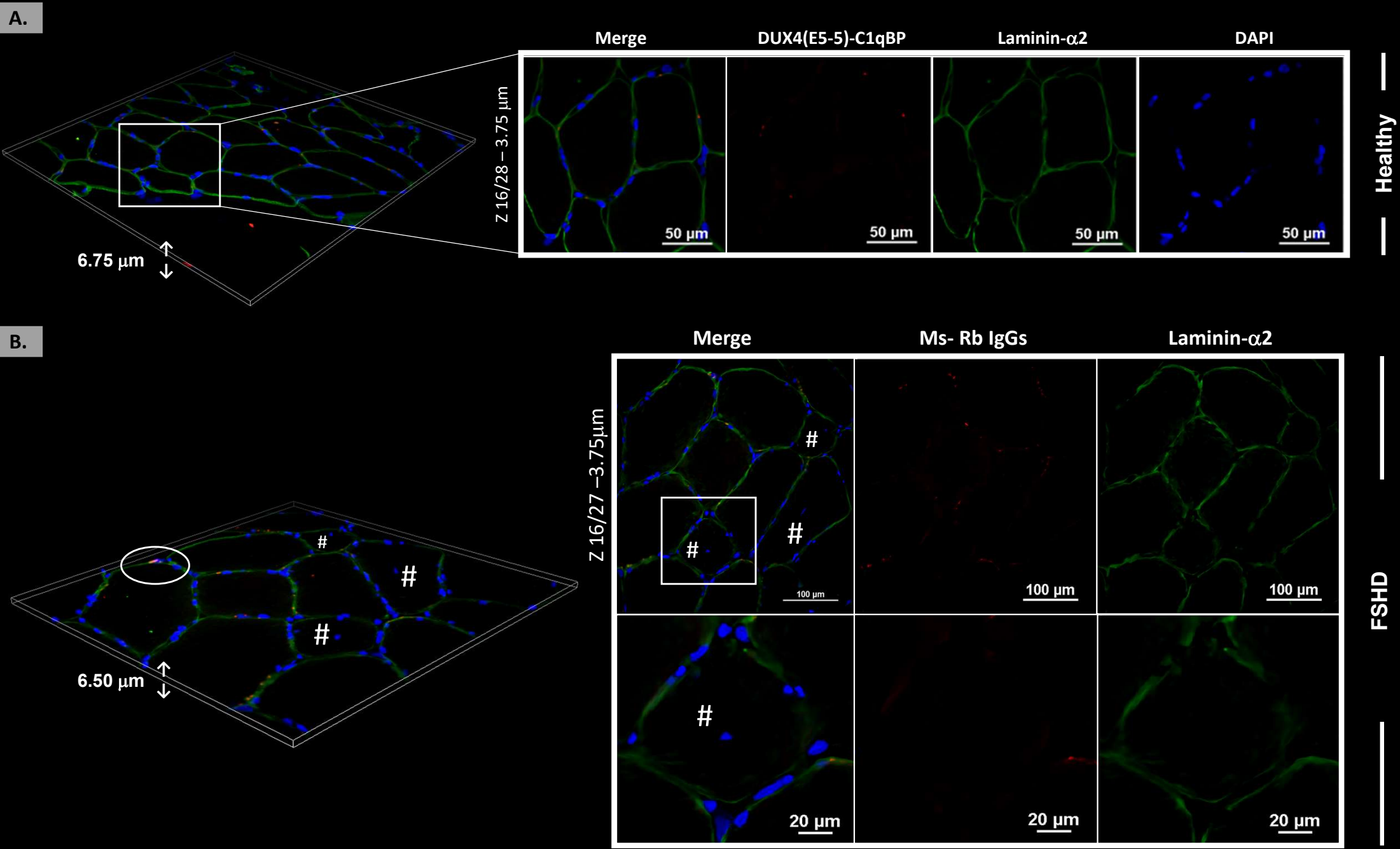

Supplement: Supplementary file 15 — Additional file 15: Figure S14. Negative PLA controls in healthy and FSHD myofibers. Negative controls used in parallel to sections of Fig. S13E-F. 3D reconstruction and an example of a Z-axis image for each negative control. (A) PLA with the pair of E5-5 (anti-DUX4 MAb) and anti-C1qBP serum on healthy control muscle sections (n=3). (B) PLA with combined mouse and rabbit IgGs in place of the primary antibody pair (n=2). Pictures were taken with the same parameters as in Fig. S13E-F and show, in the FSHD muscle section, myofibers with a delocalized nucleus (#) or an unusual shape with laminin defect (inset enlarged in the bottom panels). No PLA dots in large cluster were found. The circle in the 3D reconstruction points to nonspecific PLA signals in the lamina. Same experiment as in Fig. 7 except that the myoblasts had been cultured in a differentiation medium for 1- (A-B, n=3) or 3-days (C-D, n=3). Arrows or circle point to cytoplasmic partial co-localizations of DUX4c with the indicated RNA binding proteins. [file 13395_2022_310_MOESM15_ESM.pdf]

Fig. S15

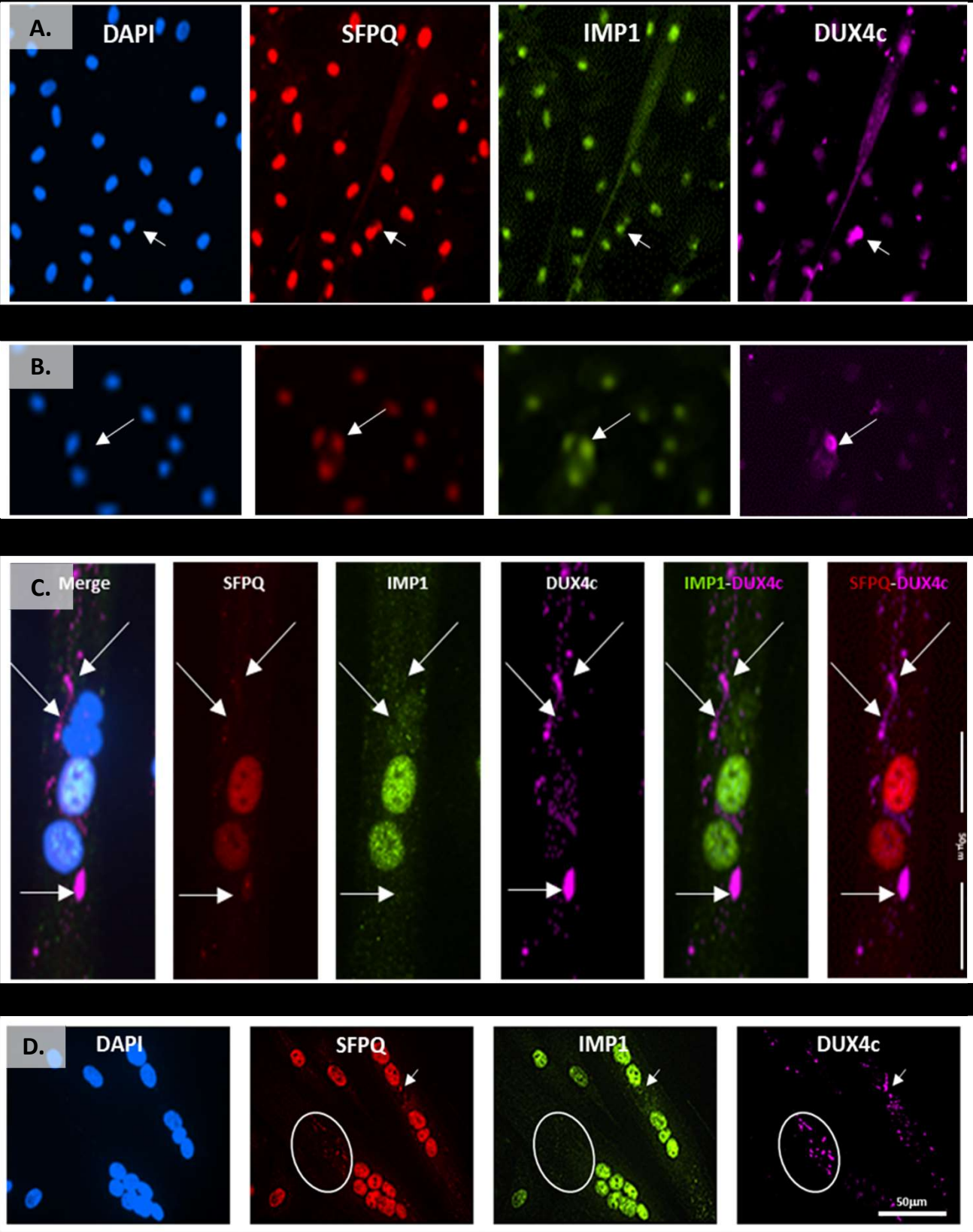

Supplement: Supplementary file 16 — Additional file 16: Figure S15. Partial co-localization of DUX4c with RNA binding proteins in differentiating muscle cells. Human testis sections were used. (A) Immunohistochemistry was performed as in Fig. S5B. The small boxed areas on the images are shown below at higher magnification. (Sg: Spermatogonia; Ser: Sertoli cells; SpI: spermatocytes I; St(1): early spermatids; St(2) late spermatids; Sz: spermatozoa). Arrows and arrowheads indicate DUX4c localization. (B-D) Immunofluorescence was performed as in Figs. 3: DUX4c (green) and ILF3/NF90 (red) were detected with specific primary antisera (see Methods) and appropriate secondary antibodies coupled to Alexa Fluor 488 or 555 respectively. (B) The boxed regions correspond to DUX4c/ILF3 partial co-localization. The star indicates a large spermatocyte I nucleus with DUX4c- and ILF3-positive nuclear spots. (C) Arrows indicate DUX4c/ILF3 partial co-localization at the nuclear periphery of a round spermatid (higher magnification of the boxed region in C in another Z axis image.) (D) Elongating spermatids (arrowheads) showing ILF3 cytoplasmic labeling. The arrow points to diffuse and weak cytoplasmic DUX4c staining. [file 13395_2022_310_MOESM16_ESM.pdf]

Fig. S16

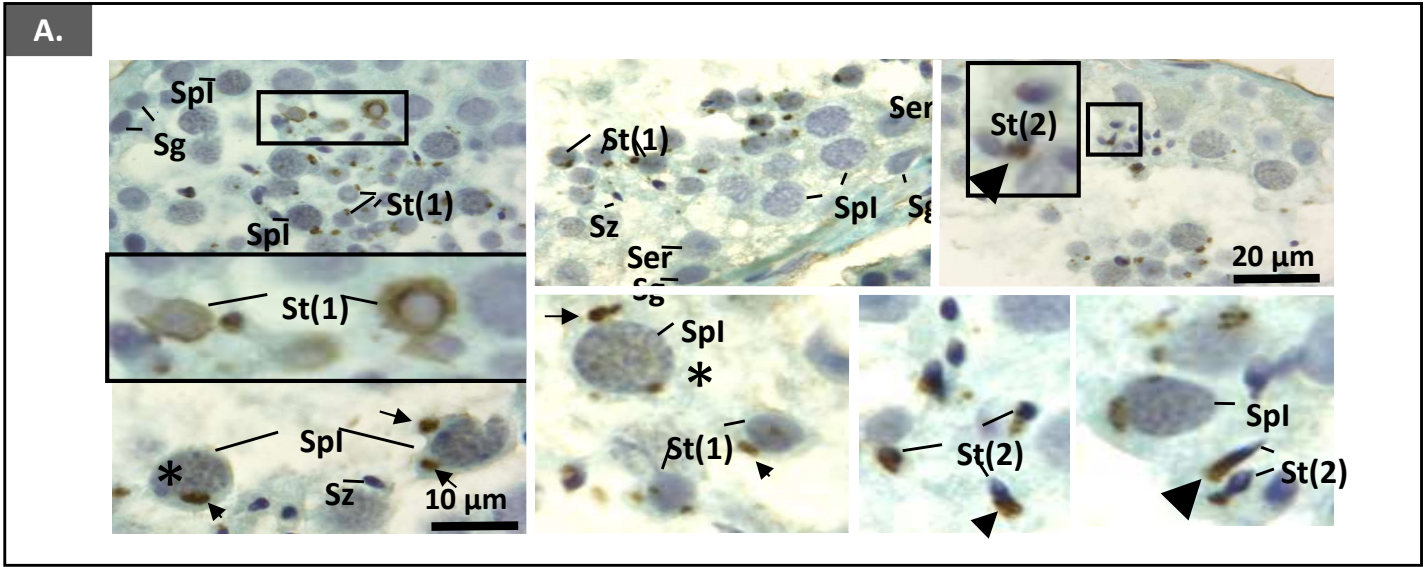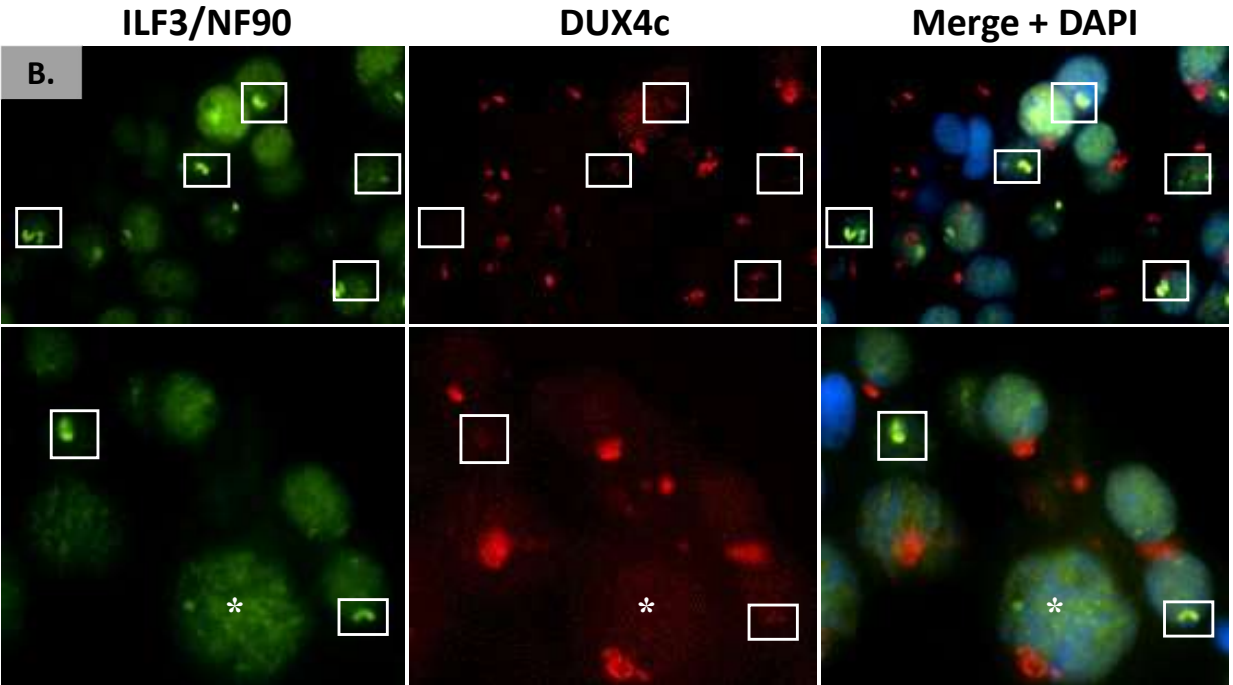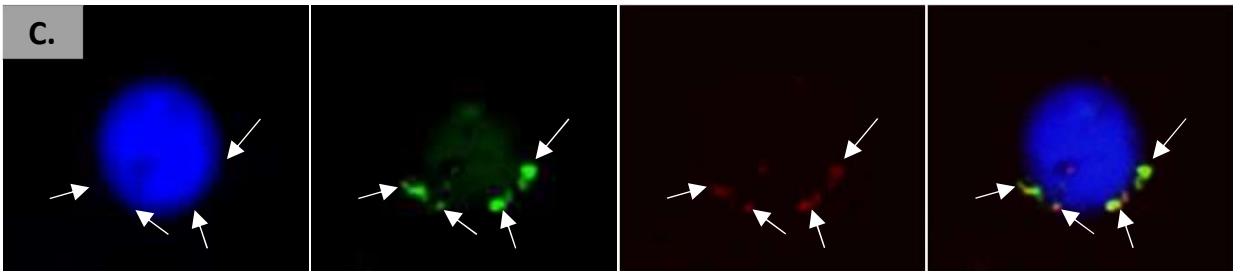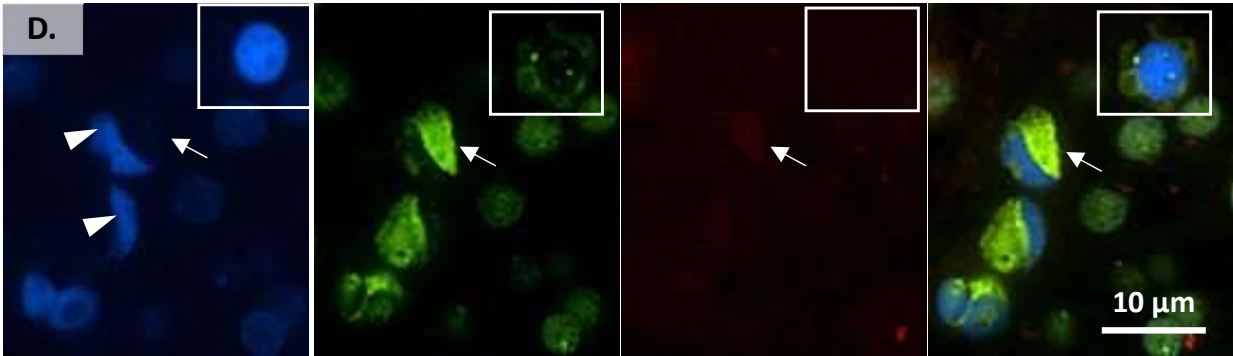

Supplement: Supplementary file 17 — Additional file 17: Figure S16. DUX4c immunodetection and partial co-localization with the RNA-binding protein ILF3/NF90 in testis. (A) Detection of slow and fast myosin. Immunofluorescence was performed on muscle sections with specific mAbs for slow and fast myosin (as described in Methods). In healthy control muscles, slow- and fast-twitch fibers present similar diameters. In the FSHD affected muscle analyzed, many slow fibers are atrophic (arrow) or necrotic (arrowhead). Ghost fibers or adipocytes are detected by their lack of myosin labeling (stars). (B) Co-immunofluorescence labeling of macrophage CD206/CD68 markers and laminin-α2 in affected muscles. Nuclei are labeled with DAPI. Few CD68+ (pro-inflammatory M1 macrophages) cells are observed (white arrows, upper panel). CD68+/CD206+ cells corresponding to M2 macrophages (yellow arrows) are much more frequent in the analyzed FSHD muscles (n=5). All macrophages observed inside FSHD muscle fibers are of M2 type (star, bottom panel). The histogram represents the percentage of M1 and M2 macrophages evaluated by counting CD68+/CD206- and CD68+/CD206+ cells on 10 microscopic fields. [file 13395_2022_310_MOESM17_ESM.pdf]

Fig. S17

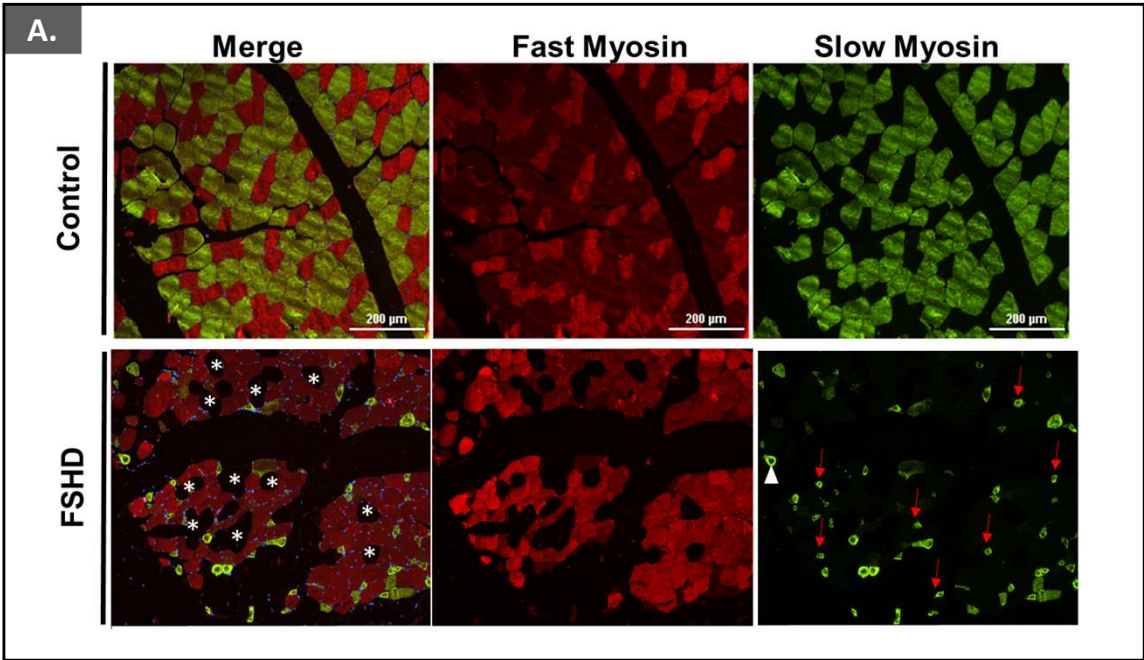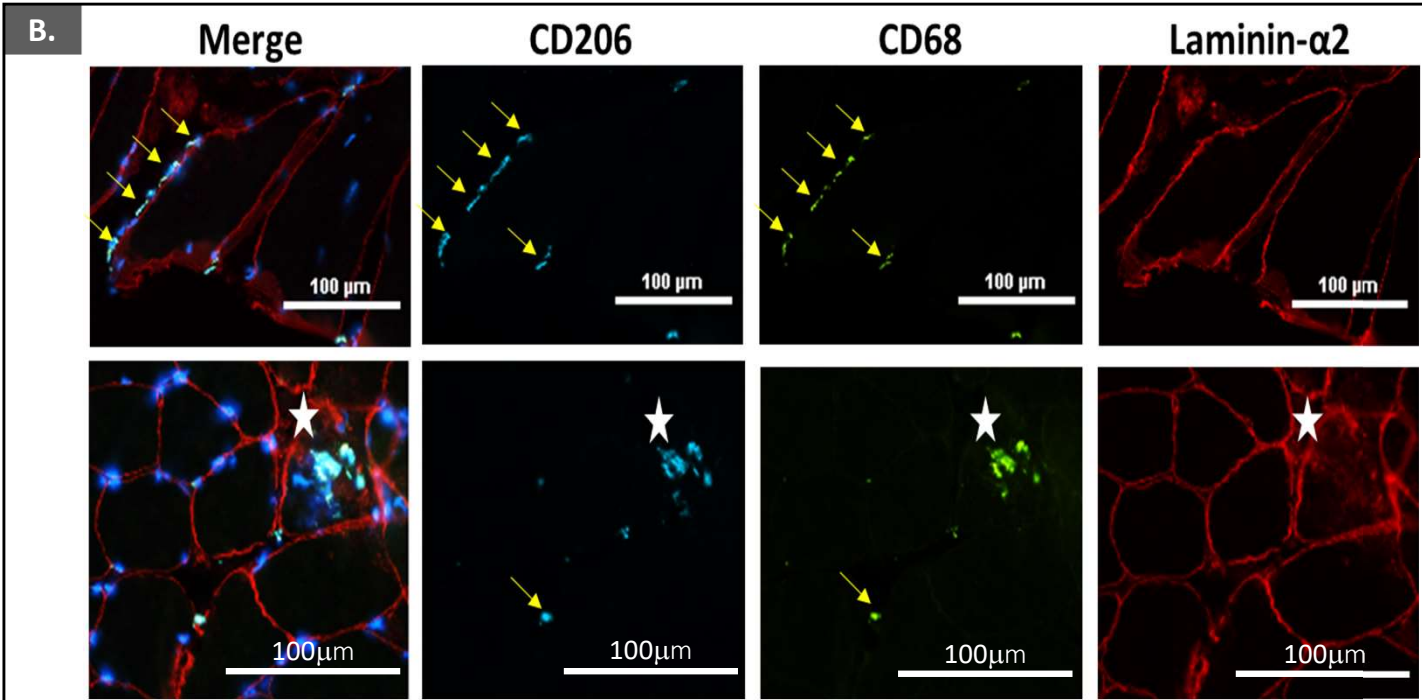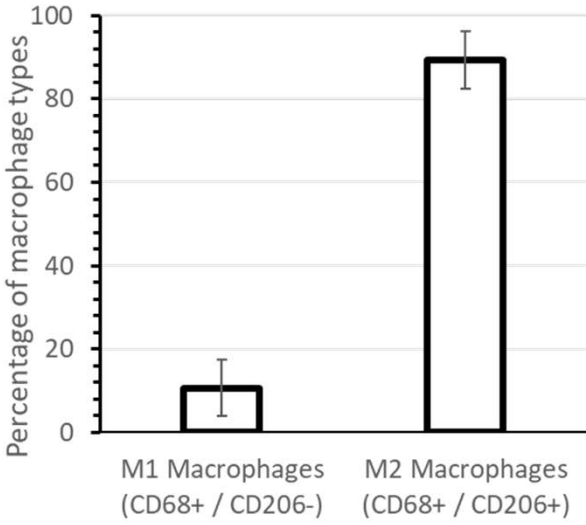

Supplement: Supplementary file 18 — Additional file 18: Figure S17. Myosin and macrophages detection in FSHD muscles. (A) Detection of slow and fast myosin. Immunofluorescence was performed on muscle sections with specific mAbs for slow and fast myosin (as described in Methods). In healthy control muscles, slow- and fast-twitch fibers present similar diameters. In the FSHD affected muscle analyzed, many slow fibers are atrophic (arrow) or necrotic (arrowhead). Ghost fibers or adipocytes are detected by their lack of myosin labeling (stars). (B) Co-immunofluorescence labeling of macrophage CD206/CD68 markers and laminin-α2 in affected muscles. Nuclei are labeled with DAPI. Few CD68+ (pro-inflammatory M1 macrophages) cells are observed (white arrows, upper panel). CD68+/CD206+ cells corresponding to M2 macrophages (yellow arrows) are much more frequent in the analyzed FSHD muscles (n=5). All macrophages observed inside FSHD muscle fibers are of M2 type (star, bottom panel). The histogram represents the percentage of M1 and M2 macrophages evaluated by counting CD68+/CD206- and CD68+/CD206+ cells on 10 microscopic fields. [file 13395_2022_310_MOESM18_ESM.pdf]
