# Supplementary material for: The double homeodomain protein DUX4c is associated with regenerating muscle fibers and RNA-binding proteins
Source: Skelet Muscle. 2023 Mar 7;13:5. doi: 10.1186/s13395-022-00310-y (PMC9990282; doi:10.1186/s13395-022-00310-y)
Supplement: Supplementary file 5 — Additional file 5: Figure S4. DUX4c immunofluorescence intensity variation during primary myoblast differentiation time-course. (A-B) Representative pictures of DUX4c detection (red) with the same parameters for the image acquisition and processing at each time point: DUX4c immunostaining intensity changed during the differentiation time-course and was culture-dependent: SPS (A) and SPI (B) primary muscle cell cultures. (C) The negative control used in parallel for the immunofluorescence with combined mouse, rat and rabbit pre-immune sera in place of the primary antibodies. Nuclei were stained with DAPI (blue). [file 13395_2022_310_MOESM5_ESM.pdf]

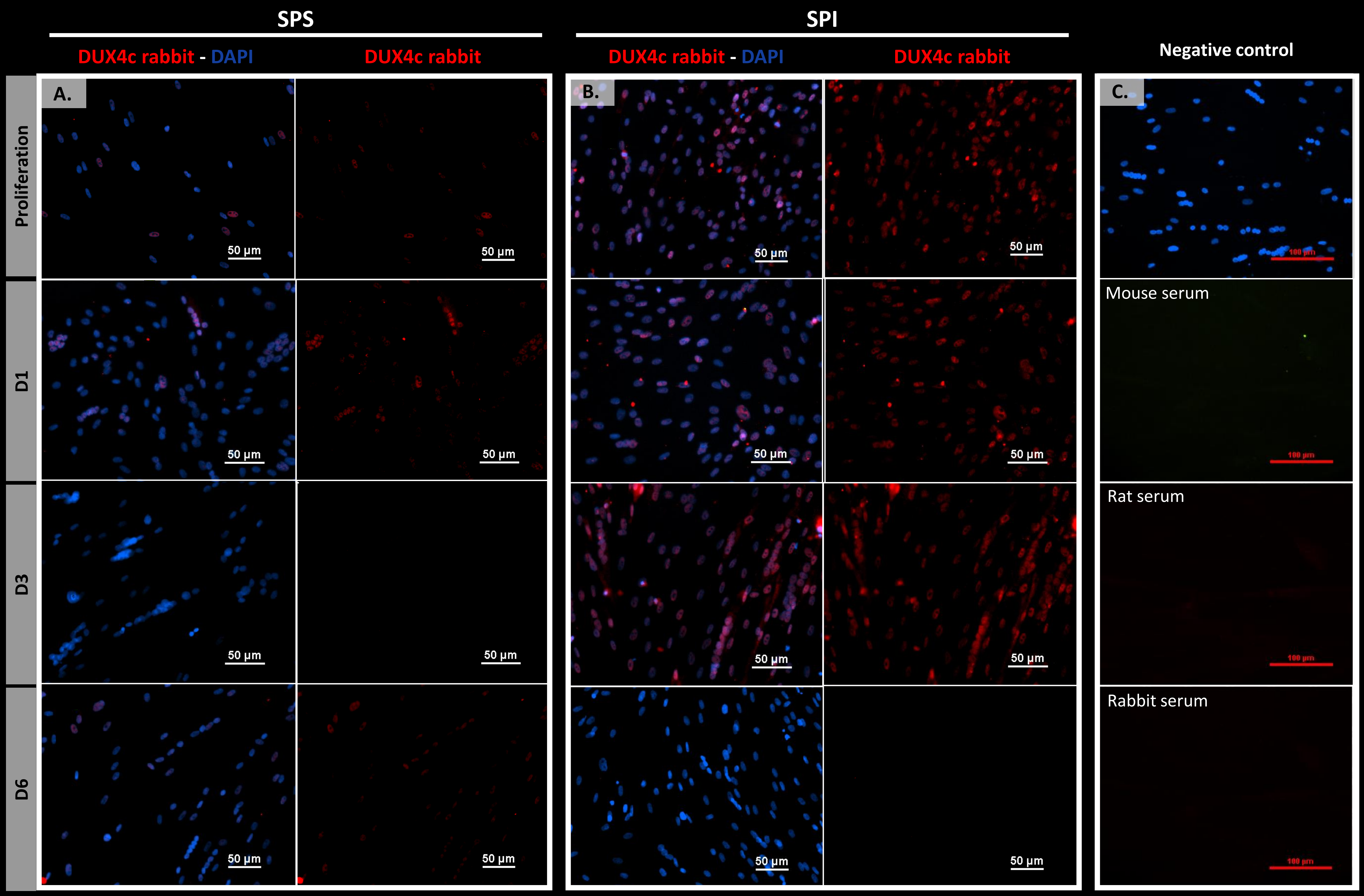
SPS
SPI
DUX4c rabbit - DAPI
DUX4c rabbit
DUX4c rabbit - DAPI
DUX4c rabbit
Negative control
A.
B.
C.
Proliferation
D1
D3
D6
50 µm
100 µm
Mouse serum
Rat serum
Rabbit serum
