# Supplementary material for: The double homeodomain protein DUX4c is associated with regenerating muscle fibers and RNA-binding proteins
Source: Skelet Muscle. 2023 Mar 7;13:5. doi: 10.1186/s13395-022-00310-y (PMC9990282; doi:10.1186/s13395-022-00310-y)
Supplement: Supplementary file 19 — Additional file 19: Table S1. Primary antibodies. Table S2. Endogenous DUX4C mRNA sequences. Table S3. GTEx data on human DUX gene expression. Table S4. Clinical and histological features of patients and muscles, including patterns of DUX4c and DUX4 staining, their co-immunodetection with regeneration markers and their interaction with C1qBP (PLA). [file 13395_2022_310_MOESM19_ESM.docx]

**Table S1: Primary antibodies.**

| **Antibody against** | **species** | **dilution** | **Manufacturer** |
| --- | --- | --- | --- |
| **C1qBP** | mouse | 1/100 (PLA in cell culture)  1/300 to 1/500 (PLA) | ab24733, Abcam |
| **C1qBP** | rabbit | 1/300 (PLA) | A302-862, Bethyl Laboratories |
| **DUX4c** | Rat (860)  Rabbit    pre-immune serum (rat or rabbit) | 1/50  1/40 (co-IF)  1/200 to 1/500 (PLA)  1/100 | Eurogentec, custom made  Ansseau et al. 2009 |
| **DUX4c/DUX4** | Rabbit | 1/50 (PLA in cell culture) | PA566173, Invitrogen |
| **DUX4** | mouse | 1/50 (co-IF) or 1/500 (PLA) | 9A12, Dixit et al. 2007 |
| **DUX4** | rabbit | 1/50 (co-IF) or 1/200 (PLA) | E5-5, Novus Biologicals/Abcam |
| **CD56** | mouse | 1/25 | Becton Dickinson |
| **CD68** | mouse | 1/200 | Abcam |
| **CD206** | rabbit | 1/50 | Abcam |
| **dMyHC** | mouse | 1/10 | NCL-MHCd, Novocastra |
| **HaloTag** | rabbit | 1/500 | Promega |
| **FUS** | mouse | 1/500 | 4H11, Santa Cruz |
| **IMP1** | rabbit | 1/200 | ab100999, Abcam |
| **ILF3** | rabbit | 1/100 | ab50832, Abcam |
| **Ki67** | mouse | 1/200 | ab8191, Abcam |
| **Laminin alpha 2** | rat | 1/200 | Sigma |
| **MYOD1** | Mouse | 1/50 or 1/200 | 5.8A, Santa Cruz |
| **SFPQ** | mouse | 1/200 | 6D7, Sigma |
| **Slow myosin** | mouse | 1/200 | NOQ7.4.5D, Sigma |
| **Fast myosin** | mouse | 1/200 | MY32, Sigma |
| **Troponin T** | mouse | 1/200 | T6277, Sigma |

**PLA:** proximity ligation assays performed on muscle sections except if indicated in cell culture

**IF:** immunofluorescence

**Table S2: Endogenous DUX4c mRNA sequences.**

Sequence of the *DUX4C* mRNA 3’ ends. The RT-PCR products were separated on agarose gel electrophoresis, the bands of the indicated sizes were cut out, the cDNAs were eluted from the gel bands and cloned into *pJET2.1* plasmid followed by sequencing.

| **PCR-band size**  Number of clones and of independent cell cultures | **Exon 1-(part of intron1)-Exon2** | **Exon 3-**adapter sequence |
| --- | --- | --- |
|  | **Positions in GenBank # AF146191.1 containing the *DUX4C* flanking genes: *FRG1* and *FRG2***  **(sequence identity)** | |
| **A. Immortalized muscle cells** | | |
| DUX4C (intron 2 spliced out)  **1.2-kb band**  2 clones  FSHD culture  differentiation | **114,483-113,807 (97% identity)**  tgagttcctgtagcacccagagtttcagcaaaaggccaggacttttcttAGATCCGGCGCCACTGGGGGAGCGAAAGACCTGGAAGAGCCCGTTTTGCTGGAACCATTCTTCAGCCCAGGAAGACCACCGGGTTTTGCTGGAGGAGCAGGTTTGAGCGGGGTTGGGGCGGGGTGGGGGCAGGACGGCGCCCTCTCTTTCGCGGTGAACCTCTGACTCGGTATGGAGAGGCGTGCCTTCCCTTCCAGCTGACCTGTCTAGGATCCCTGAGTTCCAGGTCCGGTGAGAGACTCCACACAGAGGAGGGCTGTCATTCTTTCCTGAGCATCCCGGGGATCCCAGGGCCCGCCCAGGTACCGGGAGGTGGACTGTCTACTGCGCATGCGCAGGTTTGCAGGCACTAGCCTAGGTTTTCCAACCAGCCCAGGCGGAGCTCTCATTCCTTTTTCCCCAGCGTTCTTCAGTCGAGTTGGCGGAGACCTCAGTCCGCGAAGCGCTGGGCCGGGGCAGAAGCCAGGCCAGTTCTCCTTTCCGTGGCTCGACTCCTCTGCCTCTTCGCTCACCAACACTTGCCAACCCCCGTCCCGCCAGCCTCCTCGCCAGCACCATGGAGCGCCTTGCAACTAAATGTAGACCCGAGACCCCGTGCAAACCAGGGTGCTGCCCTTTCCAGGCAAG**AG** | **113,571-113,041 (99% identity)**  **AAC**CAACAGGACCCAGCACTCCGGGAAAATGCTGGGTGCCCAGCGCGGGCTAAGTGCTGGGCCCTCCGGATCGCCAGCCTGAGTTACTTCATCCCGGAGCGATTCAGACGAATTCCGTTTCCGAAGGAATGAGCGAATTCCCCAGAGAGCAATGAGCTGAGACTCAGGTGGTTGTCCGTTTTTCATCCACATGGTTCACAGATGACATATCCCCACGTTGAGCCCTGCAACAGAGCGCGAGGCGGATAGTCCCATCCACACAGGAATCACGCTCAGGACAACTAAAGCGTGATTCTGGATTCCACGTTTCTTTGCCCTCTGCAGAGGTGCCTGTTGCTCAAGTCTCTGCCCCCGCCCCCCGAAAGTGTGACCATGTTGACTGTTTGTTTCCCGAGCTCTGTGGGGACCCAGAAACTTCCAGGAATGCGTGGAACACCAGCATCGTGTCGGTGCTCTCCTTTCCAGTTTCAAACAGGCTATATTGCAGATCCCCAATTTTGCAGGAAACAGGAATCCATCGTCAGGCCGTGAAAAAAAAAAAACCTATAGTGAGTCGTATTAATTCGGATCCGCG |
| DUX4C (introns 1&2 spliced out)  **1-kb product within the 1.2-kb band**  2 clones  FSHD culture  differentiation | **114,483-114,134 (98% identity)**  ACCAGAGTTTCAGCAAAAGGCACGGCCTTTCCTAGATCCGGCGCCACTGGGGGAGCTGAAGGACGTGGAAGAGCCCGCTCTGCTGGAACCACTCCTCAGCCAGGAAGAACACCGGGCTCTGCTGGAGGAGCAGGTTGGAGCGGGTTTGGGCGGGGTGGGGGCAGGACGGCGCCCTCTCTTTCGCGGTGAACCTCTGACTCGGTATGGAGAGGCGTGCCTTCCCTTCCAGCTGACCTGTCTAGGATCCCTGAGTTCCAGGTCCGGTGAGAGACTCCACACAGAGGAGGGCTGTCATTCTTTCCTGAGCATCCCGGGGATCCCAGGGCCCGCC**CAG**  **114,032-113,807 (100% identity)**  **CGT**TCTTCAGTCGAGTTGGCGGAGACCTCAGTCCGCGAAGCGCTGGGCCGGGGCAGAAGCCAGGCCAGTTCTCCTTTCCGTGGCTCGACTCCTCTGCCTCTTCGCTCACCAACACTTGCCAACCCCCGTCCCGCCAGCCTCCTCGCCAGCACCATGGAGCGCCTTGCAACTAAATGTAGACCCGAGACCCCGTGCAAACCAGGGTGCTGCCCTTTCCAGGCAA**GAG** | **113,571-113,041 (99% identity)**  **AAC**CAACAGGACCCAGCACTCCGGGAAAATGCTGGGTGCCCAGCGCGGGCTAAGTGCTGGGCCCTCCGGATCGCCAGCCTGAGTTACTTCATCCCGGAGCGATTCAGACGAATTCCGTTTCCGAAGGAATGAGCGAATTCCCCAGAGAGCAATGAGCTGAGACTCAGGTGGTTGTCCGTTTTTCATCCACATGGTTCACAGATGACATATCCCCACGTTGAGCCCTGCAACAGAGCGCGAGGCGGATAGTCCCATCCACACAGGAATCACGCTCAGGACAACTAAAGCGTGATTCTGGATTCCACGTTTCTTTGCCCTCTGCAGAGGTGCCTGTTGCTCAAGTCTCTGCCCCCGCCCCCCGAAAGTGTGACCATGTTGACTGTTTGTTTCCCGAGCTCTGTGGGGACCCAGAAACTTCCAGGAATGCGTGGAACACCAGCATCGTGTCGGTGCTCTCCTTTCCAGTTTCAAACAGGCTATATTGCAGATCCCCAATTTTGCAGGAAACAGGAATCCATCGTCAGGCCGTGAAAAAAAAAAAAAACCTATAGTGAGTCGTATTAATTCGGATCCGCG |
| **B. Primary cells** | | |
| *DUX4C (intron 2 spliced out)*  **1.2-kb band**  - 2/5 clones  healthy control cultures-Prolif.  -2/4 clones  healthy control cultures-Diff.  - 1/4 clones FSHD culture -Diff. | **114,483-113,814 (99% identity)**  TGAGCTCCTGTAGACACCAGAGTTTCAGCAAAAGGCACGACCTTTCCTAGATCCGGCGCCACTGGGGGAGCTGAAGGACGTGGAAGAGCCCGCTCTGCTGGAACCACTCCTCAGCCAGGAAGAACACCGGGCTCTGCTGGAGGAGCAGGTTGGAGCGGGGTTGGGGCGGGGTGGGGGCAGGACGGCGCCCTCTCTTTCGCGGTGAACCTCTGACTCGGTATGGAGAGGCGTGCCTTCCCTTCCAGCTGACCTGTCTAGGATCCCTGAGTTCCAGGTCCGGTGAGAGACTCCACACAGAGGAGGGCTGTCATTCTTTCCTGAGCATCCCGGGGATCCCAGGGCCCGCCCAGGTACCGGGAGGTGGACTGTCTACTGCGCATGCGCAGGTTTGCAGGCACTAGCCTAGGTTTTCCAACCAGCCCAGGCGGAGCTCTCATTCCTTTTTCCCCAGCGTTCTTCAGTCGAGTTGGCGGAGACCTCAGTCCGCGAAGCGCTGGGCCGGGGCAGAAGCCAGGCCAGTTCTCCTTTCCGTGGCTCGACTCCTCTGCCTCTTCGCTCACCAACACTTGCCAACCCCCGTCCCGCCAGCCTCCTCGCCAGCACCATGGAGCGCCTTGCAACTAAATGTAGACCCGAGACCCCGTGCAAACCAGGGTGCTGCCCTTTC**CAG** | **113,571-112,647 (99% identity)**  **AAC**CAACAGGACCCAGCACTCCGGGAAAATGCTGGGTGCCCAGCGCGGGCTAAGTGCTGGGCCCTCCGGATCGCCAGCCTGAGTTACTTCATCCCGGAGCGATTCAGACGAATTCCGTTTCCGAAGGAATGAGCGAATTCCCCAGAGAGCAATGAGCTGAGACTCAGGTGGTTGTCCGTTTTTCATCCACATGGTTCACAGATGACATATCCCCACGTTGAGCCCTGCAACAGAGCGCGAGGCGGATAGTCCCATCCACACAGGAATCACGCTCAGGACAACTAAAGCGTGATTCTGGATTCCACGTTTCTTTGCCCTCTGCAGAGGTGCCTGTTGCTCAAGTCTCTGCCCCCGCCCCCCGAAAGTGTGACCATGTTGACTGTTTGTTTCCCGAGCTCTGTGGGGACCCAGAAACTTCCAGGAATGCGTGGAACACCAGCATCGTGTCGGTGCTCTCCTTTCCAGTTTCAAACAGGCTATATTGCAGATCCCCAATTTTGCAGGAAACAGGAATCCATCGTCAGGCCGTGATGCACGGGACGTTTCTTTTCTCTGTGGTTTCGATCTCGTTGTCTACATGAAAGATCCCAAGACAAAGGTACTGACGGACATCCAGACACACCCCACCACAATCACTAGCAAACCCACTCCCAAACACACAGACACACACGGGCGCACGCGCGGGAACACAAGCACACACACAGACACACAAAGACACAGACAGCTTGAAGAAAAGCAAAGGACAGAGGGATGGAAAGATAGAAACGGAAGGAGAGAGAGAAACAGCGAGAGAGAGAGAGAGAGAGAGAGAGAGAGAGAGGGAGGAGAGCGGGCAAGGTGGAGAGGGAAGTAAAAAAAAAAAACCTATAGTGAGTCGTATTAATTCGGATCCGCG |
| *DUX4C (intron 2 spliced out)*  **1.2-kb band**  - 2/4 clones  healthy control culture-Prolif. | **114,483-113,802 (99% identity)**  TGAGCTCCTGTAGACACCAGAGTTTCAGCAAAAGGCACGACCTTTCCTAGATCCGGCGCCACTGGGGGAGCTGAAGGACGTGGAAGAGCCCGCTCTGCTGGAACCACTCCTCAGCCAGGAAGAACACCGGGCTCTGCTGGAGGAGCAGGTTGGAGCGGGGTTGGGGCGGGGTGGGGGCAGGACGGCGCCCTCTCTTTCGCGGTGAACCTCTGACTCGGTATGGAGAGGCGTGCCTTCCCTTCCAGCTGACCTGTCTAGGATCCCTGAGTTCCAGGTCCGGTGAGAGACTCCACACAGAGGAGGGCTGTCATTCTTTCCTGAGCATCCCGGGGATCCCAGGGCCCGCCCAGGTACCGGGAGGTGGACTGTCTACTGCGCATGCGCAGGTTTGCAGGCACTAGCCTAGGTTTTCCAACCAGCCCAGGCGGAGCTCTCATTCCTTTTTCCCCAGCGTTCTTCAGTCGAGTTGGCGGAGACCTCAGTCCGCGAAGCGCTGGGCCGGGGCAGAAGCCAGGCCAGTTCTCCTTTCCGTGGCTCGACTCCTCTGCCTCTTCGCTCACCAACACTTGCCAACCCCCGTCCCGCCAGCCTCCTCGCCAGCACCATGGAGCGCCTTGCAACTAAATGTAGACCCGAGACCCCGTGCAAACCAGGGTGCTGCCCTTTCCAGgcaagaggc**aag** | **113,571-112,647 (99% identity)**  **AAC**CAACAGGACCCAGCACTCCGGGAAAATGCTGGGTGCCCAGCGCGGGCTAAGTGCTGGGCCCTCCGGATCGCCAGCCTGAGTTACTTCATCCCGGAGCGATTCAGACGAATTCCGTTTCCGAAGGAATGAGCGAATTCCCCAGAGAGCAATGAGCTGAGACTCAGGTGGTTGTCCGTTTTTCATCCACATGGTTCACAGATGACATATCCCCACGTTGAGCCCTGCAACAGAGCGCGAGGCGGATAGTCCCATCCACACAGGAATCACGCTCAGGACAACTAAAGCGTGATTCTGGATTCCACGTTTCTTTGCCCTCTGCAGAGGTGCCTGTTGCTCAAGTCTCTGCCCCCGCCCCCCGAAAGTGTGACCATGTTGACTGTTTGTTTCCCGAGCTCTGTGGGGACCCAGAAACTTCCAGGAATGCGTGGAACACCAGCATCGTGTCGGTGCTCTCCTTTCCAGTTTCAAACAGGCTATATTGCAGATCCCCAATTTTGCAGGAAACAGGAATCCATCGTCAGGCCGTGATGCACGGGACGTTTCTTTTCTCTGTGGTTTCGATCTCGTTGTCTACATGAAAGATCCCAAGACAAAGGTACTGACGGACATCCAGACACACCCCACCACAATCACTAGCAAACCCACTCCCAAACACACAGACACACACGGGCGCACGCGCGGGAACACAAGCACACACACAGACACACAAAGACACAGACAGCTTGAAGAAAAGCAAAGGACAGAGGGATGGAAAGATAGAAACGGAAGGAGAGAGAGAAACAGCGAGAGAGAGAGAGAGAGAGAGAGAGAGAGAGAGGGAGGAGAGCGGGCAAGGTGGAGAGGGAAGTAAAAAAAAAAAACCTATAGTGAGTCGTATTAATTCGGATCCGCG |
| *DUX4C* (introns 1a, 1b & 2 spliced out)  **1-kb band**  7 clones from 1 FSHD culture - Diff. | **114,483-114,336 (100% identity)**  tgagcgcaacgcaattaattggagttagttcatcctttggcacccccagcttttccatttatgtttccggttcgtatgttGTGTGGAATTGTGAGCGGATAACAATTTCACCCAGGAAACAGCTATGACCATGATTACGCCAAGTTCAGAATTAACCCTCACTTAAAGGGACTAGTCCTGCAGGTTTAAACGAATTCGCCCTTGAGCTCCTGTAGACACCAGAGTTTCAGCAAAAGGCACGACCTTTCCTAGATCCGGCGCCACTGGGGGAGCTGAAGGACGTGGAAGAGCCCGCTCTGCTGGAACCACTCCTCAGCCAGGAAGAACACCGGGCTCTGCTGGAGGAG**CAG**  **114,238-114,134 (100% identity)**  **CTG**ACCTGTCTAGGATCCCTGAGTTCCAGGTCCGGTGAGAGACTCCACACAGAGGAGGGCTGTCATTCTTTCCTGAGCATCCCGGGGATCCCAGGGCCCGCC**CAG**  **114,032-113,814 (100% identity)**  **CGT**TCTTCAGTCGAGTTGGCGGAGACCTCAGTCCGCGAAGCGCTGGGCCGGGGCAGAAGCCAGGCCAGTTCTCCTTTCCGTGGCTCGACTCCTCTGCCTCTTCGCTCACCAACACTTGCCAACCCCCGTCCCGCCAGCCTCCTCGCCAGCACCATGGAGCGCCTTGCAACTAAATGTAGACCCGAGACCCCGTGCAAACCAGGGTGCTGCCCTTTC**CAG** | **113,571-113,037 (99% identity)**  **AAC**CAACAGGACCCAGCACTCCGGGAAAATGCTGGGTGCCCAGCGCGGGCTAAGTGCTGGGCCCTCCGGATCGCCAGCCTGAGTTACTTCATCCCGGAGCGATTCAGACGAATTCCGTTTCCGAAGGAATGAGCGAATTCCCCAGAGAGCAATGAGCTGAGACTCAGGTGGTTGTCCGTTTTTCATCCACATGGTTCACAGATGACATATCCCCACGTTGAGCCCTGCAACAGAGCGCGAGGCGGATAGTCCCATCCACACAGGAATCACGCTCAGGACAACTAAAGCGTGATTCTGGATTCCACGTTTCTTTGCCCTCTGCAGAGGTGCCTGTTGCTCAAGTCTCTGCCCCCGCCCCCCGAAAGTGTGACCATGTTGACTGTTTGTTTCCCGAGCTCTGTGGGGACCCAGAAACTTCCAGGAATGCGTGGAACACCAGCATCGTGTCGGTGCTCTCCTTTCCAGTTTCAAACAGGCTATATTGCAGATCCCCAATTTTGCAGGAAACAGGAATCCATCGTCAGGCCGTGATGCAAAAAAAAAAAACCTATAGTGAGTCGTATTAATTCGGATCCGCG |
| *DUX4c (partial sequence) with splicing* | | |
| *DUX4C* (introns 1a, 1c & 2 spliced out)  **1-kb band**  1/5  from 1 healthy control -Prolif. | **114,483-114,336 (100% identity)**  tgagcgcaacgcaattaattggagttagttcatcctttggcacccccagcttttccatttatgtttccggttcgtatgttGTGTGGAATTGTGAGCGGATAACAATTTCACCCAGGAAACAGCTATGACCATGATTACGCCAAGTTCAGAATTAACCCTCACTTAAAGGGACTAGTCCTGCAGGTTTAAACGAATTCGCCCTTGAGCTCCTGTAGACACCAGAGTTTCAGCAAAAGGCACGACCTTTCCTAGATCCGGCGCCACTGGGGGAGCTGAAGGACGTGGAAGAGCCCGCTCTGCTGGAACCACTCCTCAGCCAGGAAGAACACCGGGCTCTGCTGGAGGAG**CAG**  **114,238-114,205 (100% identity)**  **CTG**ACCTGTCTAGGATCCCTGAGTTCCAGGT**CCG**  **114,032-113,814 (100% identity)**  **CGT**TCTTCAGTCGAGTTGGCGGAGACCTCAGTCCGCGAAGCGCTGGGCCGGGGCAGAAGCCAGGCCAGTTCTCCTTTCCGTGGCTCGACTCCTCTGCCTCTTCGCTCACCAACACTTGCCAACCCCCGTCCCGCCAGCCTCCTCGCCAGCACCATGGAGCGCCTTGCAACTAAATGTAGACCCGAGACCCCGTGCAAACCAGGGTGCTGCCCTTTC**CAG** | **113,571-113,131 (99% identity)**  **AAC**CAACAGGACCCAGCACTCCGGGAAAATGCTGGGTGCCCAGCGCGGGCTAAGTGCTGGGCCCTCCGGATCGCCAGCCTGAGTTACTTCATCCCGGAGCGATTCAGACGAATTCCGTTTCCGAAGGAATGAGCGAATTCCCCAGAGAGCAATGAGCTGAGACTCAGGTGGTTGTCCGTTTTTCATCCACATGGTTCACAGATGACATATCCCCACGTTGAGCCCTGCAACAGAGCGCGAGGCGGATAGTCCCATCCACACAGGAATCACGCTCAGGACAACTAAAGCGTGATTCTGGATTCCACGTTTCTTTGCCCTCTGCAGAGGTGCCTGTTGCTCAAGTCTCTGCCCCCGCCCCCCGAAAGTGTGACCATGTTGACTGTTTGTTTCCCGAGCTCTGTGGGGACCCAGAAACTTCCAGGAATGCGTGGAACACCAGC |
| *DUX4c* (partial sequence) without splicing | | |
| **1.4-kb band**  - 6/21 clones from 4 healthy controls  -3/4 from 1 FSHD culture | **114,264-113,134 (94% identity)**  atggtagaggcggcttcctccagtgactttagtcctgattcagtcgtgaggatccaccagagaggtgtcatcttctgagcatccggggatccagggccgcccagtaccggagtggactgttactgcgctgcgcagtttgcagcactacctaggttttccaccagcccagcggagcttcattcctttttcccagcgttttcagtcgagtggcggagacctcagtccgcgaagcgctggccggcgcagaagccaggccagtttctccttccgtgggctggactcttttgcctttcggctcaccaacacttgccaacccctgtcccgccagcttcctcgccagcaccatggagcGCCTTGCAACTAAATGTAGACCCGAGACCCCGTGCAAACCAGGGTGCTGCCCTTTCCAGGCAAGAGGCAAGGCAGGCAGAGATGAGGACGGGAACGGAGACAGAGTGGGAGGGAAGGATGGAGCTAGGAAAGGATGGATGGACGGAGGGACCCTGGAAAGGAGAGAAAGAGGGAGGGAGGGAGAAAGGGAGGAAAAAACCAGGGGAGGAGGGGAAGAACAGACGGAAGGATGGACCGAGGGACAAAAGGAGCAAGAAACAGATAAAGGAAGGCAGACAGAAAAACGTTCTTCTGCCTCCAGAACCAACAGGACCCAGCATTCCGGGAAAATGCTGGGTGCCCAGCGCGGGTTAAGTGCTGGGCCTTCCGGATCGCCAGCCTGAGTTACTTCATCCCGGAGCGATTCAGACGAATTCCGTTTCCGAAGGAATGAGCGAATTCCCCAGAGAGCAATGAGCTGAGACTCAGGTGGTTGTCCGTTTTTCATCCACATGGTTCACAGATGACATATCCCCACGTTGAGCCCTGCAACAGAGCGCGAGGCGGATAGTCCCATCCACACAGGAATCACGCTCAGGACAATTAAAGCGTGATTTTGGATTCCACGTTTTTTTGCCCTCTGCAGAGGTGCCTGTTGCTCAAGTTTCTGCCCCCCCCCCCCGAAAGTGTGACCATGTTGACTGTTTGTTTCCCGAGCTCTGGGGGGACCCAGAAACTTCCAGGAATGCGTGGAACACCAAAAAAAAAAAAAAAAAAAAAAAAAAAAAAAACCTATAGTGAGTCGTATTAATTCGG | |

**Table S3:** **GTEx data** **on human *DUX* gene expression.**

| Gene (coordinates on GRCh38/hg38, strand)) | Genecode ID | Tissue/cell expression | Median TPM (n) |
| --- | --- | --- | --- |
| ***DUX4L9/4C*** (**chr4**: 190021407-190022665:-) | ENSG00000224807.5 | brain cerebellum | 1.044 (241) |
|  |  | testis | **0.07406** (361) |
|  |  | pituitary, adrenal gland, esophagus, thyroid | |
| ***DUX4L26*** (**chr3**: 75668931-75670185:+) | ENSG00000236138.4 | cultured fibroblasts | 2.136 |
|  |  | testis | 1.548 (361) |
|  |  | brain cerebellum | 0.504 (241) |
|  |  | skeletal muscle | 0.04496 (803) |
|  |  | and in a wide range of tissues | |
| ***DUX4*** (**chr4**: 190173774-190185942:+) | ENSG00000260596.5 | **only in testis** | **0.1288** (361) |
| ***DUX4L30*** (**chr4**: 190065233-190065914:+) | ENSG00000277162.1 | no expression | **n.a.** |
| ***DUX4L16-19,31***(**chrY**: 11306918-11308181:+/ 1314921-11316187:+/ 11321557-11322823:+/ 11332329-11333595:+/ 10171590-10172725:-) | ENSG00000258567.1  ENSG00000259154.1  ENSG00000259029.1  ENSG00000258991.1  ENSG00000231411.1 | **only in testis**  (no detection of *DUXL17* & *L31*) | 0.2180 to 0.09412 |
| ***DUX4L10-15, 20-25, 28-29*** (**chr 10:** 133743332-133744598:+/ 133746641-133747907:+/ 133749950-133751216:+/ 133753250-133754516:+/ 133756559-133757825:+/ 133759859-133761125:+/ 133683668-133684936:+/ 133680369-133681637:+/ 133677059-133678327:+/ 133673749-133675017:+/ 133670450-133671718:+/ 133667151-133668419:+/ 133664429-133665106:+/ 133740606-133741284:+) | ENSG00000278664.1  ENSG00000278664.1  ENSG00000278790.1  ENSG00000276046.1  ENSG00000276904.1  ENSG00000278641.1  ENSG00000276164.1  ENSG00000275610.1  ENSG00000277274.1  ENSG00000276964.1  ENSG00000274599.2  ENSG00000280337.1  ENSG00000280337.1  ENSG00000237635.2 | no expression | n.a. |
| ***DUX4L27*** (**chr12**: 34208415-34209675:-)  ***DUX4L52*** (**chr12**: 61600067-61600843:-) | ENSG00000258794.3 | testis | 4.031 (the highest level) |
|  |  | and in a wide range of tissues | |
|  | ENSG00000258336.2 | brain and testis | 0.000 |
| ***DUXL32*** (**chr20**: 29318824-29319579:+) ***DUX4L33*** (**chr20**: 29324092-29325049:+)  ***DUX4L34*** (**chr20**: 29410348-29411600:-)  ***DUX4L35*** (**chr20**: 29448517-29449306:-)  ***DUX4L37*** (**chr20**: 29878636-29879363:-) | ENSG00000283069.1 | brain and testis | 0.000 |
|  | ENSG00000282894.1 | no expression | n.a. |
|  | ENSG00000282935.1 | testis/brain | 0.3382 (361)/0.1555 (241) |
|  |  | a wide range of tissues but not skeletal muscle | |
|  | ENSG00000282911.1 | brain/testis | 0.1034 (215)/0.03986 (361) |
|  |  | a wide range of tissues but not skeletal muscle | |
|  | ENSG00000283020.1 | brain/testis | 1.700 (215)/0.1543 (361) |
|  |  | a wide range of tissues but not skeletal muscle | |
| ***DUX4L45-47*** (**chr16**: 34135736-34136792:-/34141256-34142006:-/ 34142456-34142849:+ | ENSG00000261440.1/  ENSG00000259987.1/ ENSG00000260207.1 | no expression | n.a. |
| ***DUX4L50*** (**chr9**: 63817748-63818462:-) | ENSG00000232815.1 | brain (cerebellar hemisphere) | **38.5** (215) |
|  |  | testis | 1.885 (361) |
|  |  | skeletal muscle | 0.2016 (803) |
|  |  | and in a wide range of tissues | |
| ***DUX4L51*** (**chr5**: 31249879-31250987:+) | ENSG00000250482.3 | artery, kidney, brain testis, etc. | 0.9345 to 0.02874 |
|  |  | skeletal muscl | 0.02191 (803) |
| ***DUXA*** (**chr19**: 57154021-57167443:-)  ***DUXAP10*** (**chr14**: 19284653-19337730:-) | ENSG00000258873.2 | in few tissues incl.:  testis/artery | the highest levels:  0.2/0.2454 |
|  |  | no detection in skeletal muscle | |
|  | 11 pseudogenes (P1 to 11) (chr 2,16,8,10,11,15,20) | Wide range of tissues | 1.106 the highest exp (P1) < 0.3 in other tissues |
|  | ENSG00000244306.10 | wide range of tissues | 0.7257 the highest exp |
|  |  | skeletal muscle | 0.01772 |
| ***DUXB*** (**chr16**: 75693929-75701459:-) | ENSG00000282757.3  (no isoform reported) | wide range of tissues  testis (the highest value)  skeletal muscle (the lowest value) | 0.8169 (361)  0.02342 (803) |
| ***PAX7*** (**chr1**: 18631006-18748866:+) | ENSG00000009709.11 | Brain | 0.1146 to 1.726 |
|  |  | Skeletal muscle | 1.461 |
| ***C1qBP*** (**chr17**: 5432777-5448830:-) | ENSG00000108561.8 | Skeletal muscle and a wide range of tissues (similar amount) | 85.10 |
| ***IGF2BP1*** (**chr17**: 5432777-5448830:-) | ENSG00000159217.9 | testis | 8 (361) |
|  |  | kidney, artery & other tissues | 0.4547 to 0.002010 |
|  |  | Skeletal muscle | 0.005720 (803) |

GTEx Analysis Release V8 (dbGaP Accession phs000424.v8.p2 on 2020-09-07) :

Samples were collected from 54 non-diseased tissue sites across nearly 1000 individuals,

primarily for molecular assays including WGS, WES, and RNA-Seq.

**Table S4. Clinical and histological features of patients and muscles, including patterns of DUX4c and DUX4 staining, their co-immunodetection with regeneration markers and their interaction with C1qBP (PLA)**

The clinical severity score (CSS) is a 10-grade clinical severity scale developed by Ricci et al. (1999). This score takes into account the extent of weakness in various body regions and considers the descending spread of symptoms from face and shoulders to pelvic and leg muscles typical of FSHD (0=unaffected to 10=severely affected). The percentage of fat in each muscle was also evaluated by magnetic resonance imaging (MRI). The histology score (HS) was based on observations of 10-μm sections stained with Hematoxylin & Eosin (H&E) and was established on the following criteria: variability in fiber size, extent of central nucleation, necrosis/regeneration, interstitial fibrosis and inflammation. Each histological characteristic was ranked from 0 to 3 (0 = normal; 1= mild; 2=moderate; and 3=severe) (qualitative data). The total HS corresponds to the attributed pathology grade and results from the sum of the scores obtained for each histological characteristic. This yields a score between 0 and 15 (0 = normal to 15= severe dystrophic changes) (Statland et al., 2015). The percentage of hypotrophic (A <30μm) and hypertrophic (H >100μm) fibers was determined where indicated for each muscle fiber type on H&E-stained sections.

CT: connective tissue surface area determined by the average percentage of the connective tissue, in blue, reported to the total tissue surface, blue & red; myofibers in red; Blue Trichrome staining, **Fig. S13B**).

Total fibers were counted on the basis of a partial or complete laminin-α2 staining around muscle cells and the central/delocalized nuclei (DN) determined by DAPI staining inside a laminin-α2 outline only in sections where transversal fibers were found. The numbers of positive fibers for either DUX4c or ‘DUX4’ were overestimated as the observed microscope fields were selected on the basis of a clear DUX4c, DUX4 or PLA signal detection. The threshold used to define positive PLA dots in clusters was an area larger than 30 µm^2^, an area size that was never observed in the healthy control sections used in parallel.

Fiber axis lengths: microfibers (5-10 µm to 10-15 µm); hypotrophic fibers (15-30 µm in at least one axis); normal-size fibers (30-100 µm)

The analyzed muscles were classified into four groups: (1) weakly affected muscles (total histology score: 1 to 6, CSS ≤ 5, MRI fat fraction <10%); (2) strongly affected muscles (total histology score: 7 to 11, CSS >5, MRI fat fraction >10%); (3) affected muscles from patient who have undergone an omopexia surgery and (4) muscles from uncharacterized patients. Additional information about patients F2 to F10 is available in Lassche et al. (Neurology 2020).

| ID (STIR) | Muscle | CSS | MRI fat % | (%)  Type 1 fibers | (%)  Type 2 fibers | HS | CT  Average ± SD | Number (fibers with DN/ total fibers)  **Pattern of DUX4c detection**  **Desmin co-detection & PLA**  (71 DN in 1,136 total fibers analyzed)  *~110 DUX4c-positive fibers* | | **DUX4 detection using 9A12**  **& PLA** (as indicated)  (755 total fibers analyzed)  *~60 DUX4 (9A12)-positive fibers* |
| --- | --- | --- | --- | --- | --- | --- | --- | --- | --- | --- |
| 1. **Patients with FSHD presenting a CSS ≤** 5 (mild inflammation indicated * in HS) | | | | | | | | | | |
| F7 (-) | VL | 2 | 1 | A0.5 / **H1.0** | A0 / **H8.7** | 4* | 8,4 ± 1,5 | 5/121 myofibers  **DUX4c staining found:**  - in 4 DN (**Fig. S6B**) of 3 fibers  -in 3 adjacent normal size fibers: either dots at the membrane or around large peripheral nuclei (**Fig. S7C**),  with desmin co-detection  or in abnormal tips | | total of 37 fibers  **staining in dots:**  - in 2 flat microfibers  - at the periphery of 3 normal size fibers in abnormal tips (containing or not a nucleus)  - inside a double lamina or around two close by nuclei in 2 adjacent fibers  (with part of the lamina missing)  *Cluster of dots often grouped in short lines at fiber peripheries*  **PLA E5-5-C1qBP**  (548 total fibers analyzed)  42x PLA dots mainly in 2 clusters of fibers at their periphery and in abnormal tips (**Fig. S13F**) |
| F13 (-) | VL | 5 | 0 | A1.7 / **H30.3** | A0 / **H15.4** | 5* | 9,8 ± 1,8 | 1/154 myofibers  **DUX4c staining found:**   - next to membrane either around DN - or in dots in the sarcoplasm - (13 stained areas in 3 clusters of 2-3 fibers near hypofiber or fiber with DN)   - in hypotrophic (11) or normal-size (5) fibers in clusters, some in co-detection with desmin or in abnormal tips including a cluster of nuclei (**Fig. S7D-E**)  **PLA DUX4c-C1qBP**  (140 myofibers analyzed)  PLA dots:  -7x at the fiber periphery  -2x in abnormal tips | | total of 67 fibers  No detection  **PLA E5-5-C1qBP**  (112 myofibers analyzed)  13x PLA dots in cluster at the fiber periphery including abnormal tips  (**Figs. 8B, S13E**) |
| F11 (-) | TA | 2 | 6 | **A4** / H0.5 | A0 / **H3.6** | 2* | 13,8 ± 6,1 | 5/153 myofibers  **DUX4c staining found:**  -in hypotrophic (4) and adjacent normal size (12 partial staining) fibers  - in 1 normal size fiber with desmin co-detection at the periphery in abnormal tips | | Total of 104 fibers  **staining in dots:**  - in 2 microfibers, both next to few dots in adjacent fibers at the site of fuzzy laminin staining  -at the periphery of 2 normal size fibers at the site of fuzzy laminin staining  and at 6 abnormal tips (next to 1 or several nuclei)  *Clusters of dots often grouped in short lines at fiber peripheries*  **PLA E5-5-C1qBP**  (196 myofibers analyzed)  14x PLA dots next to peripheral nuclei or inside clusters of nuclei (at their periphery) |
| F4 (-) | TA | 3 | 10 | A0 / **H4.6** | A0 / **H8.7** | 4 | 28,3 ± 9,1 | 7 DN/67 myofibers  **DUX4c staining found: with** co-detection of CD56 in 33/34 small CD56 -positive cells either next to a cluster of 4 (in a longitudinal section) or 7 fibers  or at a myofiber tip (**Fig. S9C**)  **PLA DUX4c-C1qBP**  (225 myofibers analyzed)  14x PLA dots:  -inside a MP or between nuclei in cluster at an abnormal tip **(Fig. 7)**  - in cluster next to large peripheral nuclei | | total of 152 fibers  **staining in dots:**  several dots around a large nucleus at the periphery in a single fiber with an abnormal shape |
| F8 (-) | TA | 4 | 1 | A1.2 / **H12.0** | A0 / **H3.6** | 2 | 12,3 ± 6,3 | *(section 1)* 5 DN /73 myofibers  next to CD56-positive cells  **DUX4c staining found:**  - with co-detection of CD56 in 10/16 CD56-positive cells  next to 3 clusters of 2-4 fibers  *(section 2)* 5 DN/208 myofibers  **DUX4c staining found:**  - at the periphery of 8 fibers in clusters: small areas around nuclei iincluding 6 areas next to a nucleus in an angular or round abnormal tip.  **PLA DUX4c-C1qBP**  (48 myofibers analyzed)  No detection | | total of 14 fibers  **staining in dots:**  - in 2 large peripheral nuclei  - Around 3 to 5 aligned nuclei of various sizes  either ‘inside’ a longitudinal fiber  or at the periphery of another fiber  - in 4 hypotrophic fibers in 2 clusters  (**Fig. S11B**) |
| 1. **Patients with FSHD presenting a CSS** > **5**  (mild inflammation: indicated * in HS) | | | | | | | | | | |
| F9 (+) | VL | 8 | 53 | **A12.6** / H 0.5 | **A42.7** / H7.0 | 10* | 19.4 ± 3.9 | *(section 1)* 1 DN /15 myofibers  **DUX4c staining found:**  - at the fiber periphery or in dots next to peripheral nuclei or tip in partial co-localization with desmin in a cluster of 6 fibers (including 1 flat hypotrophic, 1 rectangular and 1 with DN) (**Fig. 3A**)  *(section 2)* 9DN/72 myofibers  **DUX4c staining found:**  - at the fiber periphery or in dots next to peripheral nuclei or tips in 4 hypotrophic  and 5 larger fibers | | *(section 1)* total of 103 fibers  **staining in dots:**  -next to nuclei or at the periphery  6 groups of 2-3 hypotrophic fibers  and 1 group of 10 hypotrophic fibers  *(section 2)* total of 67 fibers  **staining in dots:**  -at the periphery next to peripheral nuclei in a cluster of 5 fibers (including one hypotrophic, one with DN & one with a large and fuzzy laminin staining at the site of 9A12 epitopes)  -2 hypotrophic fibers (20 µm diameter) both next to 4 fibers, with a few dots at the periphery, one with an abnormal shape and one with DN |
| F9 (-) | VL | 8 | 55 | **A 2.3** / H1.8 | A3.2 / **H11.0** | 8 | 17.9 ± 7.4 | 7DN/70  **DUX4c staining found:**  - in and around a cluster of 5 delocalized nuclei  - at the fiber periphery  or in dots next to peripheral nuclei or tips  in 3 clusters of 2-6 hypotrophic fibers | | n.d. |
| F10 (n.d.) | TA | 6 | 64 | **A20** / H0.4 | **A2.2** / H0 | 7 | 21.3 ± 9.5 | 24/159  **DUX4c staining found:**  - near DN in 1 normal-size myofiber  (**Fig. S6C**)  - at the fiber periphery or in dots next to/around peripheral nuclei or tips in co-detection with desmin in 4 clusters of 3-10 hypotrophic and normal-size myofibers  (**Figs. 3B-C, S6D-F, S7A-B**) | | total of 118 fibers  **staining in dots:**  - around nuclei, inside the sarcoplasm or at the periphery in 5 aligned microfibers next to fibers with DN  (**Fig. S11A**)  - between 5 nuclei in cluster inside a single microfiber (next to 6 fibers with DN and abnormal shape)  - 2 micro & 2 hypotrophic fibers next to a longitudinal fiber showing 9A12 staining around 3 aligned nuclei in a shrunk fiber part (**Fig. 6A**, **S11B**)  **PLA: E5-5-C1qBP**  2x PLA dots in nearby area of two adjacent muscle cells that seem to be muscle progenitors (**Fig 8A**) |
|  |  |  |  |  |  |  |  | **PLA: DUX4 (9A12)-C1qBP**  76 myofibers analyzed  10x PLA dots in cluster (**Fig. S13D**) | | |
| F2 (-) | TA | 7 | 20 | **A10.6** / H10.1 | A0 / **H3.2** | 7 | 19.8 ± 7.3 | n.d.  **PLA: DUX4c-C1qBP**  (97 myofibers analyzed)  PLA dots in cluster:  - 1 in an abnormal tip with cluster of nuclei  -1 near a large periphery nucleus | | **Fig. S6A:** cluster of hypofibers surrounded by lamina  **PLA: E5-5-C1qBP**  (32 myofibers analyzed)  1 PLA dot in a hypotrophic fiber that seems to fuse |
| F1 (-) | TA | 6 | 80 |  |  | 9 |  | 7/44  **DUX4c staining found:**  around a large nucleus  and extending as a line | | total of 93 fibers  **epitope detection in dots:**  next to nuclei in clusters |
| CO-01 | VL | n.a. | | | | | n.d | **PLA DUX4c-C1qBP**  385 myofibers analyzed | | **PLA E5-5-C1qBP** |
| CO-02 | VL |  |  |  |  |  | n.d |  | | **PLA E5-5-C1qBP** |
| CO-05 | VL |  |  |  |  |  | n.d |  | | **PLA E5-5-C1qBP** |
| CO-07 | VL |  |  |  |  |  | n.d | **PLA DUX4c-C1qBP**  385 myofibers analyzed | | **PLA E5-5-C1qBP** |
| CO-08 | VL |  |  |  |  |  | n.d | **PLA DUX4c-C1qBP**  240 myofibers analyzed | |  |
| 1. **Patients who have undergone an omopexia surgery** (mainly used for immunohistochemistry) | | | | | | | | | | |
| F-P1 (n.d.)      F-P1 | I | n.d. | n.d. | n.d. | n.d. |  |  | Strong nuclear staining (**Fig.** **S5C**)  Cluster of CD56^+^-cells (**Fig. S9E**) | | n.d. |
|  | SS | n.d. | n.d. | A* | n.d. |  |  | - Nuclear/Sarcomeric staining in normal/hypotrophic myofibers (**Figs. S5B**)  - Co-detection with dMYHC (**Fig. 4**) | | n.d. |
|  | IS | n.d. | n.d. | n.d. | n.d. |  |  | Not detected (**Fig.** **S5C**) | | E5-5 staining (**Fig. S11D**)  co-detection with MYOD  (**Figs. 6B-C, S11E**) |
|  | SPS | n.d. | n.d. | n.d. | n.d. | Primary culture, MFI 14,2% | | | n.d. | n.d. |
| F-P2 (n.d.) | SPI | n.d. | n.d. | n.d. | n.d. | Primary culture, MFI 70,2 % | | | - Strong nuclear staining  (**Fig.** **S5C**)  - co-detection with dMyHC & MYOD (**Figs. 5, S9A-B**)  - cluster of CD56+-cells (**Fig. S9D**) |  |
|  | SA | n.d. | n.d. | n.d. | n.d. |  | | |  |  |
| F-P3 | D | n.d. | n.d. | n.d. | n.d. |  | | | n.d |  |
| **(4) uncharacterized patients** | | | | | | | | | | |
| FSHD1 (92123) | Q |  |  |  |  |  | 7 ± 1,9 |  | Rare |  |
| FSHD2 (9703) | Q |  |  |  |  |  |  |  | Rare |  |
| FSHD3 (9729) | Q |  |  |  |  |  |  |  | Rare |  |
| FSHD4 (5726) | T |  |  |  |  |  | 15,7 ± 5,6 |  | Rare |  |
| FSHD5 (5720) | T |  |  |  |  |  |  |  | Rare |  |
| FSHD6 (1988A) | T |  |  |  |  |  |  |  | Rare |  |
| DMD1 (0203) | Q |  |  |  |  |  | 37,9 ± 16,7 |  | Sarcomeric staining |  |
| DMD2 (mc1) | Q |  |  |  |  |  |  |  | Sarcomeric staining |  |
| DMD3 (mc2) | Q |  |  |  |  |  |  |  | Sarcomeric staining |  |
| C1 | Q |  |  |  |  |  | 4,4 ± 2 |  | Not detected |  |
| C2 | Q |  |  |  |  |  |  |  | Not detected |  |
| C3 to C8 | Q/D |  |  |  |  |  | About 1-2% |  | Not detected |  |

VL: vastus lateralis - TA: tibialis anterior- SPI: serratus posterior inferior- I: intercostalis- SS: sub-scapularis- IS: infraspinatus - SPS: Serratus Posterior Superior- Q: Quadriceps - T: trapezius – D: deltoid.

A (a-/hypo-trophic fiber) - H (hypertrophic fiber) - *a lot of atrophic fibers positive for slow myosin (**Fig. S17A**) ; DN: delocalized nuclei; F: FSHD; C or CO: control; DMD: Duchenne Muscle Dystrophy; MFI: myogenic fusion index (number of nuclei in myotubes divided by the total number of nuclei); MP: myogenic progenitor; n.d.: not determined
